# Association between body fat distribution and asthma in adults: results from the cross-sectional and bidirectional Mendelian randomization study

**DOI:** 10.3389/fnut.2024.1432973

**Published:** 2024-07-22

**Authors:** Kang Wang, Zhujun Chen, Zhengxiao Wei, Lijun He, Liang Gong

**Affiliations:** ^1^Department of Respiratory and Critical Care Medicine, The First Affiliated Hospital of Army Medical University, Chongqing, China; ^2^Department of Clinical Laboratory, Public Health Clinical Center of Chengdu, Chengdu, China; ^3^Department of Ultrasound, The Second Affiliated Hospital of Army Medical University, Chongqing, China

**Keywords:** body fat distribution, adult asthma, cross-sectional study, Mendelian randomization, fat mass

## Abstract

**Background:**

Many studies define obesity based on body mass index (BMI) and explore its relationship with adult asthma. However, BMI only considers height and weight, ignoring other factors such as body fat, which may have a greater impact on health. We investigated the relationship between body fat distribution and adult asthma using both a cross-sectional study and bidirectional Mendelian randomization (MR) analysis.

**Methods:**

Weighted logistic regression models were used to examine the relationship between body fat distribution measurements and adult asthma in the cross-sectional study from National Health and Nutrition Examination Survey (NHANES) 2011–2018. Restricted cubic spline (RCS) curves were employed to explore the dose–response relationship between them. The inverse-variance weighted (IVW) method was used as the main method of MR analysis to explore the causal effect of exposure on outcome.

**Results:**

After adjusting for all covariates, weighted logistic regression analysis indicated that fat mass in the left arm, left leg, right arm, right leg, trunk, and total body is associated with an increased risk of developing adult asthma (*p* < 0.05). RCS curves showed that all six fat mass indicators exhibit a J-shaped relationship with adult asthma. Forward MR analysis found a causal effect of six fat mass indicators on the increased risk of adult asthma (*p* < 0.05). However, reverse MR did not reveal any causal effect of adult asthma on these six fat mass indicators (*p* > 0.05).

**Conclusion:**

Our study supports a positive correlation and a unidirectional causality between body fat distribution measurements and the risk of adult asthma. Further studies are needed to validate our findings.

## 1 Introduction

Asthma persists as a widespread chronic respiratory condition globally, impacting the quality of life of millions of people (1). The global burden of asthma is considerable, with estimates suggesting that it affects approximately 262 million people and causes around 461,000 deaths annually (2). This burden is not only reflected in healthcare costs but also includes lost productivity and decreased activity levels. Moreover, the impact of asthma on adults is particularly profound, as it can complicate the management of other comorbid conditions such as cardiovascular disease and diabetes, which are more prevalent with advancing age (3). Unlike childhood asthma, which is frequently characterized by atopy and a clear allergic component, asthma in adults can present with a broader range of triggers and is often less responsive to conventional treatments (4). Thus, recognizing risk factors for asthma in adults and implementing suitable interventions are crucial for decreasing both the morbidity and mortality associated with asthma.

Obesity represents a severe worldwide health issue that has experienced a significant rise in prevalence during recent decades (5). Obesity in adults is often evaluated using the body mass index (BMI), where a BMI of 30 kg/m^2^ or more is considered obese. This condition is not only a risk factor for traditional metabolic syndromes but also complicates respiratory conditions (6). Numerous studies have demonstrated that obesity raises the risk of developing asthma (7–9). After controlling for other variables, observational research conducted in the US discovered that obesity was an independent risk factor for asthma (8). A meta-analysis that included seven cohort studies with more than 333,102 participants showed that an increase in BMI had a dose–response effect on the incidence of asthma (9). While most research on obesity and asthma classifies participants solely based on BMI, this basic and frequently used anthropometric measure has significant limitations. Because it fails to differentiate between adipose mass and muscle mass, nor does it consider the distribution of body fat (10). Some studies (11, 12) have even found a U-shaped association between BMI and asthma, meaning that having a BMI that is too high or too low may both raise the chance of developing asthma. In certain instances, BMI might either overestimate or underestimate obesity. Therefore, new measures are required in order to correctly examine the link between obesity and asthma risk. Body fat distribution measurements have been used to predict conditions such as diabetes, cardiovascular disease, and other conditions with superior predictive value to BMI (13, 14). However, there is a dearth of evidence on the connection between fat distribution and adult asthma.

The National Health and Nutrition Examination Survey (NHANES) is a collection of surveys conducted at different times to evaluate the well-being of the U.S. population, track disease patterns and risk factors, and provide data to support the development and evaluation of health policies and programs (15). Mendelian Randomization (MR) is a method to evaluate the causal link between exposure and outcome. It uses genes strongly associated with traits as instrumental variables (IVs) to infer the causal relationship indirectly. Since genes are randomly allocated during gamete production and are minimally influenced by external environments after birth, MR can avoid confounding factors, reverse causation, and selection bias (16). To determine the impact of body fat distribution measurements (arm, leg, trunk, and total fat mass) on adult asthma risk, we first analyzed cross-sectional data from the NHANES database in this research. Afterwards, the causal association between them was evaluated using a bidirectional MR study.

## 2 Methods

### 2.1 Individuals involved in NHANES

**Figure 1:**
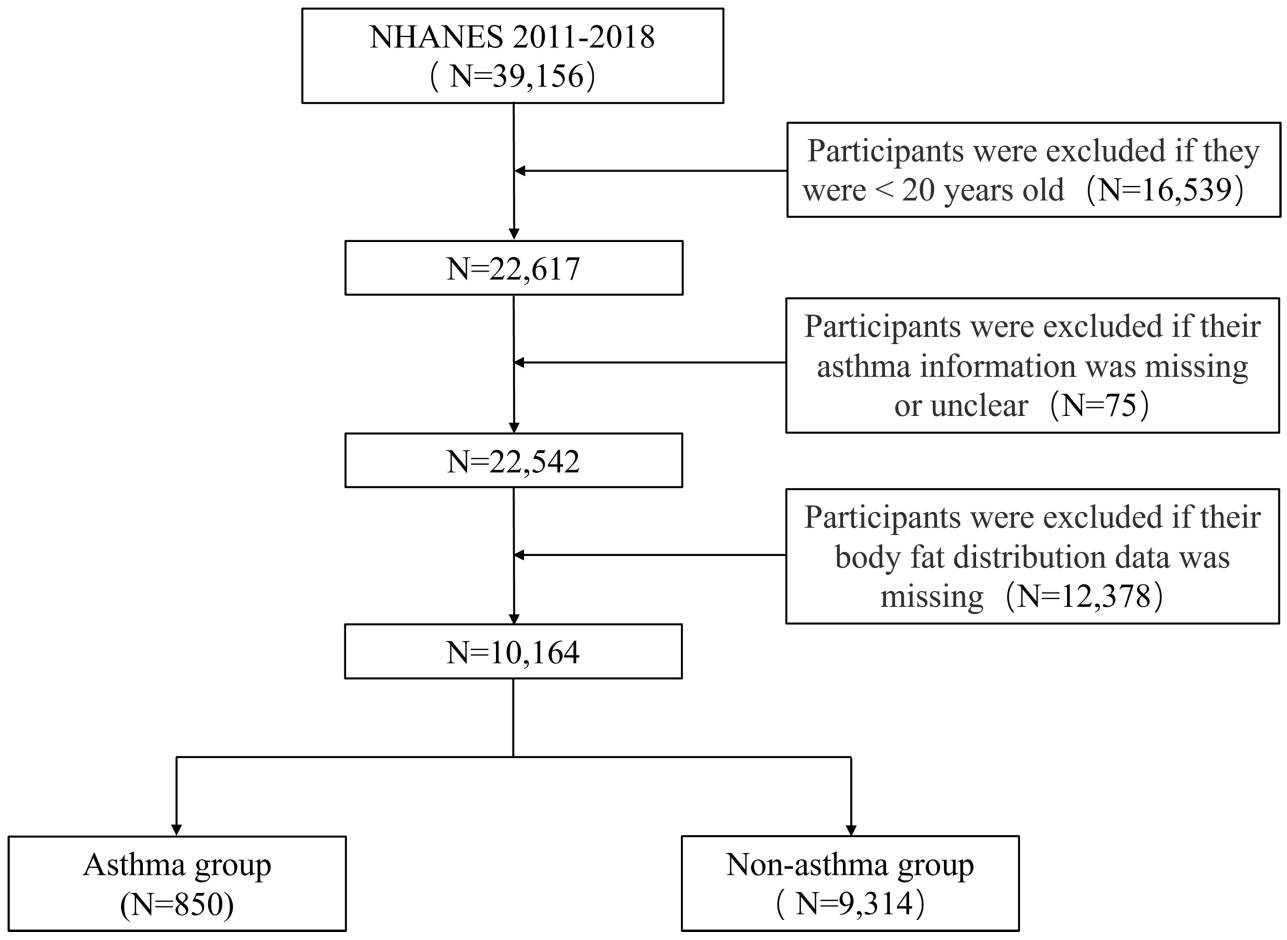
Flowchart for screening eligible participants in the cross-sectional study from NHANES 2011–2018. NHANES, National Health and Nutrition Examination Survey.

NHANES is a biennial survey that uses a random sampling technique to include people of all ages, genders, races, and socioeconomic statuses. Oral interviews, physical exams, and laboratory tests make up the inquiry, which covers a wide range of health-related subjects including chronic disorders, dietary habits, environmental exposure, and more (15). A total of 39,156 respondents participated in the NHANES surveys that were carried out between 2011 and 2018.^1^ Our study included 10,164 participants after removing people under the age of 20, people with uncertain asthma status, and people with missing body fat distribution data. A detailed description of the participant selection procedure is provided in Figure 1.

### 2.2 Variable descriptions in the NHANES

The two main questions that help establish an asthma diagnosis are as follows: “(I) Has a doctor or other health professional ever told you that you have asthma? (II) Do you still have asthma?” Participants who answered positively to both inquiries were classified as asthma patients, whereas those who did not were labeled as non-asthma patients. Multiple peer-reviewed studies have shown the accuracy of using self-reported methods to diagnose asthma (17–19). Arm, leg, trunk and total fat mass are measured using dual-energy X-ray absorptiometry (DXA), which is widely used for body composition measurements because of its quickness, convenience of use, and low levels of radiation. Data on gender, age, race, educational level, poverty-income ratio (PIR), asthma family history, smoking history, diabetes, hypertension and hypercholesterolemia were collected via a questionnaire. Three categories exist based on smoking history: non-smoker (fewer than 100 cigarettes smoked in a lifetime), former smoker (100 cigarettes smoked in a lifetime but have since quit), and current smoker (100 cigarettes smoked in a lifetime and are continuing smoking). Results for BMI were derived from physical examination data.

### 2.3 Study design of bidirectional MR

**Figure 2:**
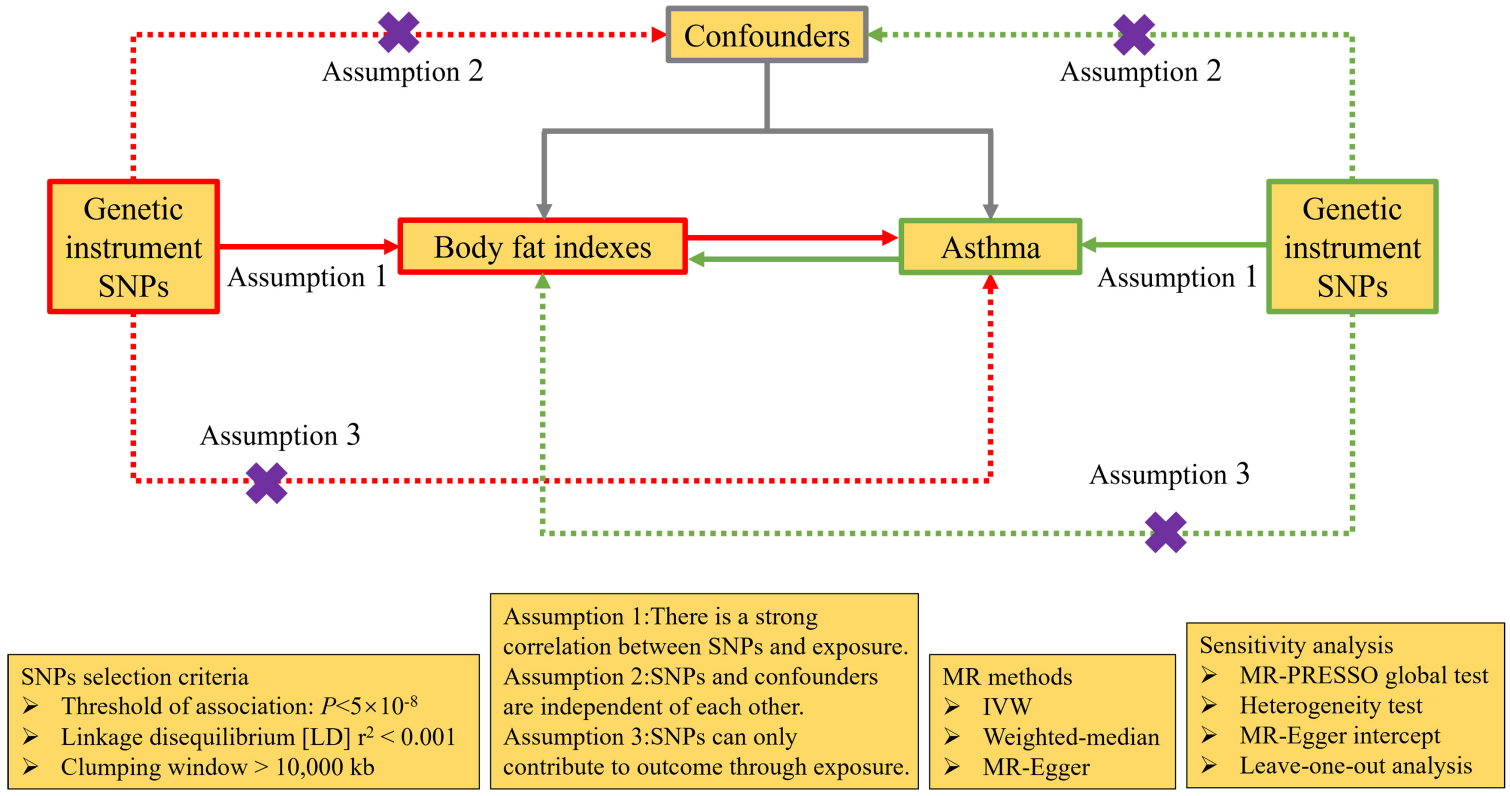
The design of bidirectional MR analysis. Purple cross indicates that this pathway cannot be allowed. MR, Mendelian randomization; SNPs, single nucleotide polymorphisms; IVW, inverse-variance weighted; MR-PRESSO, Mendelian Randomization Pleiotropy RESidual Sum and Outlier.

Based on the summary data from the Genome Wide Association research (GWAS), this research used a bidirectional MR analysis to examine the causal connection between fat distribution and adult asthma. The forward MR analysis used fat distribution measurements as the exposure and adult asthma as the outcome, while the reverse MR analysis used adult asthma as the exposure and fat distribution measurements as the outcome. IVs assessing the relationship need to fulfil three criteria: (I) strong correlation with exposure; (II) absence of associations with potential confounding factors; (III) no direct association with outcome, influencing outcome solely through exposure (Figure 2).

### 2.4 Data sources

FinnGen R10 is the source of the GWAS data on adult asthma, and the study has a total of 156,078 European population samples, with 135,449 samples belonging to the control group and 20,629 samples belonging to the case group. The genetic data of fat distribution measurements were gathered from the UK Biobank database. The cohorts of left arm, left leg, right arm, right leg, trunk, and total fat mass included 331,164, 331,275, 331,226, 331,293, 331,093, and 330,762 European population samples, respectively (Supplementary Table S1).

### 2.5 Instrumental variables selection

Single nucleotide polymorphisms (SNPs) should have a significant correlation with the corresponding exposure before being selected as independent genetic IVs. The selection criteria include a significance level of *p* < 5 × 10^−8^ and linkage disequilibrium *r*^2^ < 0.001 (10,000 kb). Next, we screened the SNPs according to the following steps:(I) using PhenoScanner to identify SNPs associated with confounding factors and outcomes, which were then excluded; (II) palindromic SNPs were excluded through the process of harmonization; (III) The MR-PRESSO test was employed to find outlier SNPs, which were then excluded. Additionally, to avoid the bias caused by weak IVs, we employed the F statistic to determine their strength and F statistic > 10 was required. *F* = *R*^2^(*N*−2)/(1−*R*^2^); *R*^2^ = (2 × β^2^ × eaf × (1−eaf))/[(2 × β^2^ × eaf × (1−eaf)) + (2 × N × SE^2^ × eaf × (1−eaf))]. *N* represents the sample size; β represents the beta value of the IVs; SE represents the standard error of β; eaf represents the effect allele frequency of the IVs (20, 21).

### 2.6 Statistical analysis

Statistical analysis was carried out using the R software, specifically version 4.3.1. Complex sampling designs and sample weights were utilized by NHANES to guarantee that the data acquired was representative of the nation as a whole. Actual frequencies and weighted percentages (%) were employed to express categorical variables, and weighted chi-square test was utilized to compare groups. Weighted *t*-test was used to compare continuous variables, which were presented as means and 95% confidence intervals (CIs). We tested the hypothesis that elevated fat mass was associated with an increased risk of asthma in adult using three weighted logistic regression models. Crude model was univariate analysis; Model 1 was adjusted for gender, age, race, educational level and PIR. Model 2 built on Model 1 and further adjusted for BMI, asthma family history, smoking history, diabetes, hypertension and hypercholesterolemia. The results of the weighted logistic regression were expressed in terms of odds ratios (ORs), indicating the risk of asthma associated with an increase of 1 kg in fat mass. To go a step further in evaluating the correlation, we fitted logistic regression models with restricted cubic splines (RCS).

The inverse-variance weighted (IVW) method is the most efficient way to find causation in MR analysis. As a result, we used it as our primary technique, turning to the IVW fixed-effects model in cases where there was no evidence of IVs heterogeneity. In addition to the IVW method, MR Egger and weighted median approaches were also employed as supplements (22). Considering multiple exposure factors, to enhance the credibility of the results, we used the Bonferroni method to adjust the MR results, deeming *p* < 0.008 (0.05/6) as statistically significant. To evaluate heterogeneity between SNPs, we used Cochran’s Q test; a *p*-value > 0.05 and an *I*^2^ < 25% suggested no substantial heterogeneity (23). Outlier SNPs could be found using the MR-PRESSO test, and a *p*-value > 0.05 for the global test meant that there were no outlier SNPs (24). An intercept near to zero (*p* > 0.05) suggested that there was no potential horizontal pleiotropy of SNPs when the MR-Egger method was employed to assess them (25). Using a Leave-one-out approach, we sought to determine if a single SNP impacted the overall exposure-outcome relationship. More than that, we used funnel plots to assess the resultant stability. R software’s “TwoSampleMR” package was used to do MR analyses. *p* < 0.05 was considered statistically significant.

## 3 Results

### 3.1 Baseline features of the participants

Ten thousand one hundred and sixty-four people in all were enrolled in this research, and their mean age was 39.10 (38.61, 39.59) years. Of these, 51.7% were men and 48.3% were women. Among people with asthma, there are 850 individuals, with a prevalence rate of 8.4%. The asthma group had larger percentages of females, Non-Hispanic Whites, and family history of asthma in comparison to the non-asthma group (*p* < 0.05). They also had higher BMI, lower PIR, and more fat mass in the left arm, left leg, right arm, right leg, trunk, and total body (*p* < 0.05). Nonetheless, the two groups did not vary in terms of age, educational level, smoking history, diabetes, hypertension, and hyperlipidemia (*p* > 0.05) (Table 1).

**Table 1:** Weighted clinical features of the participants.

Variables	Total (*n* = 10,164)	Asthma group (*n* = 850)	Non-asthma group (*n* = 9,314)	*p*
Gender, *n* (%)				<0.001
Male	5,177 (51.7)	307 (35.8)	4,870 (53.2)	
Female	4,987 (48.3)	543 (64.2)	4,444 (46.8)	
Age, years, mean (95% CI)	39.10 (38.61, 39.59)	38.44 (37.18, 39.69)	39.16 (38.66, 39.66)	0.265
Race, *n* (%)				<0.001
Mexican American	1,529 (10.6)	79 (6.3)	1,450 (11.0)	
Other Hispanic	1,065 (7.5)	80 (6.3)	985 (7.6)	
Non-Hispanic White	3,479 (60.9)	361 (66.0)	3,118 (60.4)	
Non-Hispanic Black	2,093 (11.1)	212 (13.2)	1,881 (10.9)	
Other races	1,998 (9.9)	118 (8.2)	1,880 (10.1)	
Educational level, *n* (%)				0.604
Under high school	1,876 (13.2)	144 (11.9)	1,732 (13.3)	
High school or equivalent	2,214 (21.5)	200 (22.1)	2,014 (21.5)	
College or above	6,074 (65.3)	506 (66.0)	5,568 (65.2)	
PIR, *n* (%)				0.004
<1.3	3,032 (21.9)	328 (28.7)	2,704 (21.2)	
1.3–3.5	3,363 (32.3)	239(29.9)	3,124 (32.5)	
>3.5	2,959 (39.3)	222 (35.5)	2,737 (39.6)	
Not record	810 (6.5)	61 (5.9)	749 (6.6)	
BMI, kg/m^2^, *n* (%)				0.008
<25	3,260 (31.9)	235 (31.1)	3,025 (31.9)	
25–29.9	3,255 (32.9)	229 (28.2)	3,026 (33.3)	
≥30	3,617 (35.0)	384 (40.6)	3,233 (34.5)	
Not record	32 (0.2)	2 (0.1)	30 (0.2)	
Asthma family history, *n* (%)				<0.001
Yes	2,226 (22.5)	407 (49.2)	1,819 (20.0)	
No	7,761 (75.8)	413 (48.1)	7,348 (78.3)	
Not record	177 (1.7)	30 (2.7)	147 (1.7)	
Smoking history, *n* (%)				0.131
Non-smoker	6,210 (59.2)	468 (55.2)	5,742 (59.5)	
Former smoker	1,699 (19.5)	142 (19.0)	1,557 (19.6)	
Current smoker	2,255 (21.3)	240 (25.8)	2,015 (20.9)	
Diabetes, *n* (%)				0.115
Yes	721 (5.3)	85 (7.6)	636 (5.1)	
No	9,259 (92.9)	745 (90.6)	8,514 (93.1)	
Borderline	184 (1.8)	20 (1.8)	164 (1.8)	
Hypertension, *n* (%)				0.203
Yes	2,306 (21.5)	237 (23.7)	2,069 (21.3)	
No	7,858 (78.5)	613 (76.3)	7,245 (78.7)	
Hypercholesterolemia, *n* (%)				0.425
Yes	2,422 (24.6)	225 (26.7)	2,197 (24.4)	
No	7,699 (75.1)	622 (73.0)	7,077 (75.3)	
Not record	43 (0.3)	3 (0.3)	40 (0.3)	
Left arm fat mass, kg, mean (95% CI)	1.66 (1.63, 1.69)	1.86 (1.77, 1.95)	1.64 (1.61, 1.67)	<0.001
Left leg fat mass, kg, mean (95% CI)	4.77 (4.71, 4.84)	5.31 (5.10, 5.52)	4.72 (4.66, 4.79)	<0.001
Right arm fat mass, kg, mean (95% CI)	1.67 (1.64, 1.70)	1.87 (1.78, 1.96)	1.65 (1.63, 1.68)	<0.001
Right leg fat mass, kg, mean (95% CI)	4.90 (4.83, 4.97)	5.43 (5.23, 5.64)	4.85 (4.78, 4.92)	<0.001
Trunk fat mass, kg, mean (95% CI)	13.15 (12.91, 13.40)	14.39 (13.65, 15.13)	13.04 (12.80, 13.27)	<0.001
Total fat mass, kg, mean (95% CI)	27.33 (26.91, 27.75)	30.01 (28.71, 31.30)	27.08 (26.67, 27.49)	<0.001

CI, confidence interval; PIR, poverty-income ratio; BMI, body mass index.

### 3.2 Associations between fat mass and adult asthma

**Figure 3:**
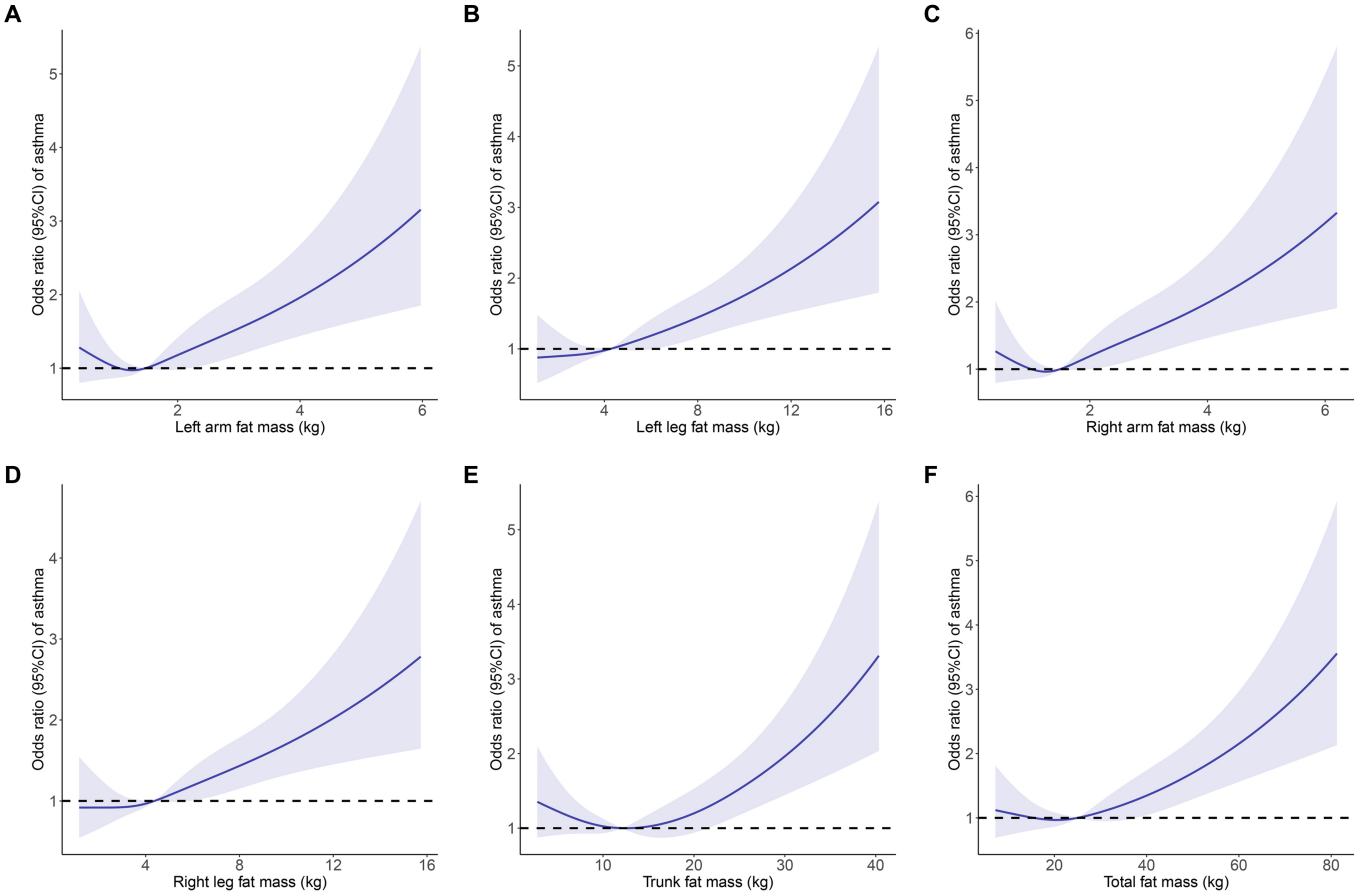
Restricted cubic spline curve (4 knots) to identify the relationship between body fat distribution measurements and adult asthma. **(A)** Left arm fat mass; **(B)** Left leg fat mass; **(C)** Right arm fat mass; **(D)** Right leg fat mass; **(E)** Trunk fat mass; **(F)** Total fat mass. Solid blue curves are multivariable-adjusted OR, with light blue area showing 95% CI. The reference line for no association is indicated by dashed black line at a OR of 1.0. The association was adjusted for gender, age, race, educational level, PIR, BMI, asthma family history, smoking history, diabetes, hypertension and hypercholesterolemia. OR, odds ratios; CI, confidence interval; PIR, poverty-income ratio; BMI, body mass index.

The link between fat mass and adult asthma was tested using weighted logistic regression. As shown in Table 2, the higher the fat mass, the greater the risk of developing asthma. The results from Model 2, which adjusted for all covariates, indicated that each kilogram increase in fat mass of the left arm, left leg, right arm, right leg, trunk, and total body was associated with a 21.9, 6.3, 21.4, 5.6, 2.6, and 1.1% increased risk of adult asthma, respectively (all *p* < 0.05). Similarly, RCS curve analysis, after adjusting for all covariates, showed that all six fat mass indicators exhibit a J-shaped relationship with adult asthma, where the risk of asthma increases as fat mass rises (Figure 3).

**Table 2:** Weighted logistic regression model analysis of the correlation between fat mass and adult asthma.

Models	Crude model		Model 1		Model 2	
	OR (95%CI)	*p*	OR (95%CI)	*p*	OR (95%CI)	*p*
Left arm fat mass	1.344 (1.209–1.494)	<0.001	1.230 (1.091–1.386)	0.001	1.219 (1.027–1.446)	0.024
Left leg fat mass	1.127 (1.086–1.170)	<0.001	1.074 (1.029–1.121)	0.002	1.063 (1.009–1.120)	0.022
Right arm fat mass	1.340 (1.206–1.490)	<0.001	1.230 (1.094–1.383)	0.001	1.214 (1.024–1.439)	0.026
Right leg fat mass	1.123 (1.083–1.164)	<0.001	1.069 (1.026–1.115)	0.002	1.056 (1.006–1.108)	0.029
Trunk fat mass	1.033 (1.017–1.049)	<0.001	1.029 (1.013–1.046)	0.001	1.026 (1.009–1.043)	0.007
Total fat mass	1.021 (1.013–1.029)	<0.001	1.015 (1.007–1.024)	<0.001	1.011 (1.005–1.019)	0.006

Crude model, not adjusted; Model 1, adjusting for gender, age, race, educational level and PIR; Model 2, adjusting for Model 1+ BMI, asthma family history, smoking history, diabetes, hypertension and hypercholesterolemia. OR, odds ratio; CI, confidence interval; PIR, poverty-income ratio; BMI, body mass index.

### 3.3 Causal effects of fat mass on adult asthma

**Figure 4:**
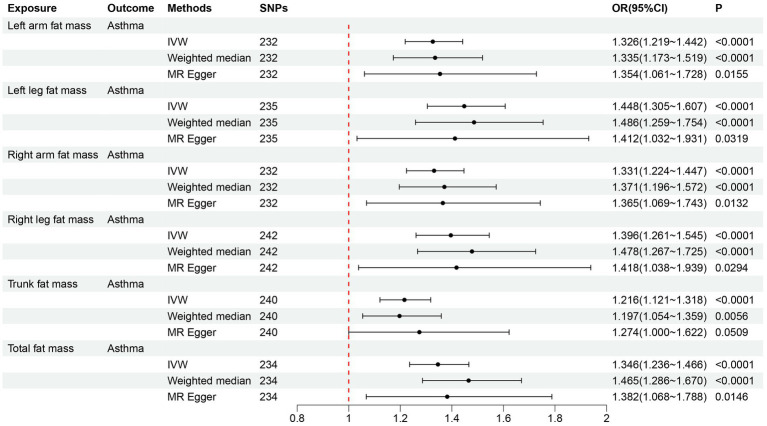
Forward MR analysis results for the causal effect of six fat mass indicators on adult asthma. MR, Mendelian randomization; SNPs, single nucleotide polymorphisms; IVW, inverse-variance weighted; OR, odds ratio; CI, confidence interval.

After filtering, the left arm, left leg, right arm, right leg, trunk, and total fat mass cohorts included 232, 235, 232, 242, 240, and 234 SNPs, respectively, for MR analysis. All SNPs had F-statistics > 10 (Supplementary Tables S2–S7). Following Bonferroni adjustment (*p* < 0.008), the results from the IVW method indicated that increases in left arm fat mass (OR = 1.326, 95% CI = 1.219–1.442, *p* < 0.001), left leg fat mass (OR = 1.448, 95% CI = 1.305–1.607, *p* < 0.001), right arm fat mass (OR = 1.331, 95% CI = 1.224–1.447, *p* < 0.001), right leg fat mass (OR = 1.396, 95% CI = 1.261–1.545, *p* < 0.001), trunk fat mass (OR = 1.216, 95% CI = 1.121–1.318, *p* < 0.001), and total fat mass (OR = 1.346, 95% CI = 1.236–1.466, *p* < 0.001) were associated with an increased risk of adult asthma (Figure 4). The estimated causal effects of each SNP on adult asthma are shown in Supplementary Figures S1–S6.

The MR-PRESSO global test *p*-values for all six fat mass indicators were larger than 0.05 after eliminating outlier SNPs, suggesting that there was no horizontal pleiotropy (Table 3). In the heterogeneity test, there was no heterogeneity among the SNPs in the six causal relationships (all *p* > 0.05, *I*^2^ < 25%) (Table 3). The MR-Egger regression intercepts for all six fat mass indicators were not statistically different from zero (all *p* > 0.05), suggesting that there was no horizontal pleiotropy of SNPs (Table 3; Supplementary Figure S7). Supplementary Figures S8–S13 showed that in the leave-one-out analysis, no SNPs significantly affected the total impact. Supplementary Figure S14 show that there was no pleiotropy since the funnel plot was symmetrical. Our MR findings were reliably confirmed by all sensitivity evaluations.

**Table 3:** Sensitivity analysis of MR.

Exposure	Outcome	MR-PRESSO			MR-Egger			Cochran Q test			Rucker’s Q’ test	
		Casual estimate	sd	Global test *p*	Intercept	se	*p*	*Q* value	*p*	*I* ^2^	*Q* value	*p*
Left arm fat mass	Asthma	0.282	0.040	0.897	−4.55E-04	0.003	0.856	204.912	0.891	12.7%	204.879	0.882
Left leg fat mass	Asthma	0.370	0.051	0.811	4.35E-04	0.003	0.867	214.565	0.814	9.1%	214.537	0.802
Right arm fat mass	Asthma	0.286	0.040	0.871	−5.57E-04	0.003	0.825	204.734	0.893	12.8%	204.686	0.884
Right leg fat mass	Asthma	0.334	0.049	0.899	−2.75E-04	0.003	0.917	212.959	0.903	13.2%	212.948	0.895
Trunk fat mass	Asthma	0.195	0.039	0.846	−0.001	0.003	0.688	215.282	0.863	11.0%	215.121	0.854
Total fat mass	Asthma	0.297	0.041	0.919	−5.52E-04	0.003	0.833	205.228	0.905	13.5%	205.183	0.897
Asthma	Left arm fat mass	0.001	0.007	0.584	−0.006	0.004	0.157	11.203	0.594	16.0%	8.922	0.710
Asthma	Left leg fat mass	−0.001	0.006	0.352	−0.004	0.004	0.266	14.651	0.330	11.3%	13.160	0.358
Asthma	Right arm fat mass	0.003	0.007	0.486	−0.007	0.004	0.147	12.346	0.500	5.3%	9.938	0.621
Asthma	Right leg fat mass	−0.001	0.007	0.324	−0.004	0.004	0.291	14.531	0.338	10.5%	13.188	0.356
Asthma	Trunk fat mass	0.008	0.009	0.100	−1.62E-04	0.005	0.976	21.286	0.095	34.2%	21.284	0.067
Asthma	Total fat mass	0.003	0.008	0.446	−0.004	0.004	0.351	12.820	0.462	1.4%	11.877	0.456

MR, Mendelian randomization; MR-PRESSO, Mendelian Randomization Pleiotropy RESidual Sum and Outlier; sd, standard deviation; se, standard error.

### 3.4 The reverse MR

**Figure 5:**
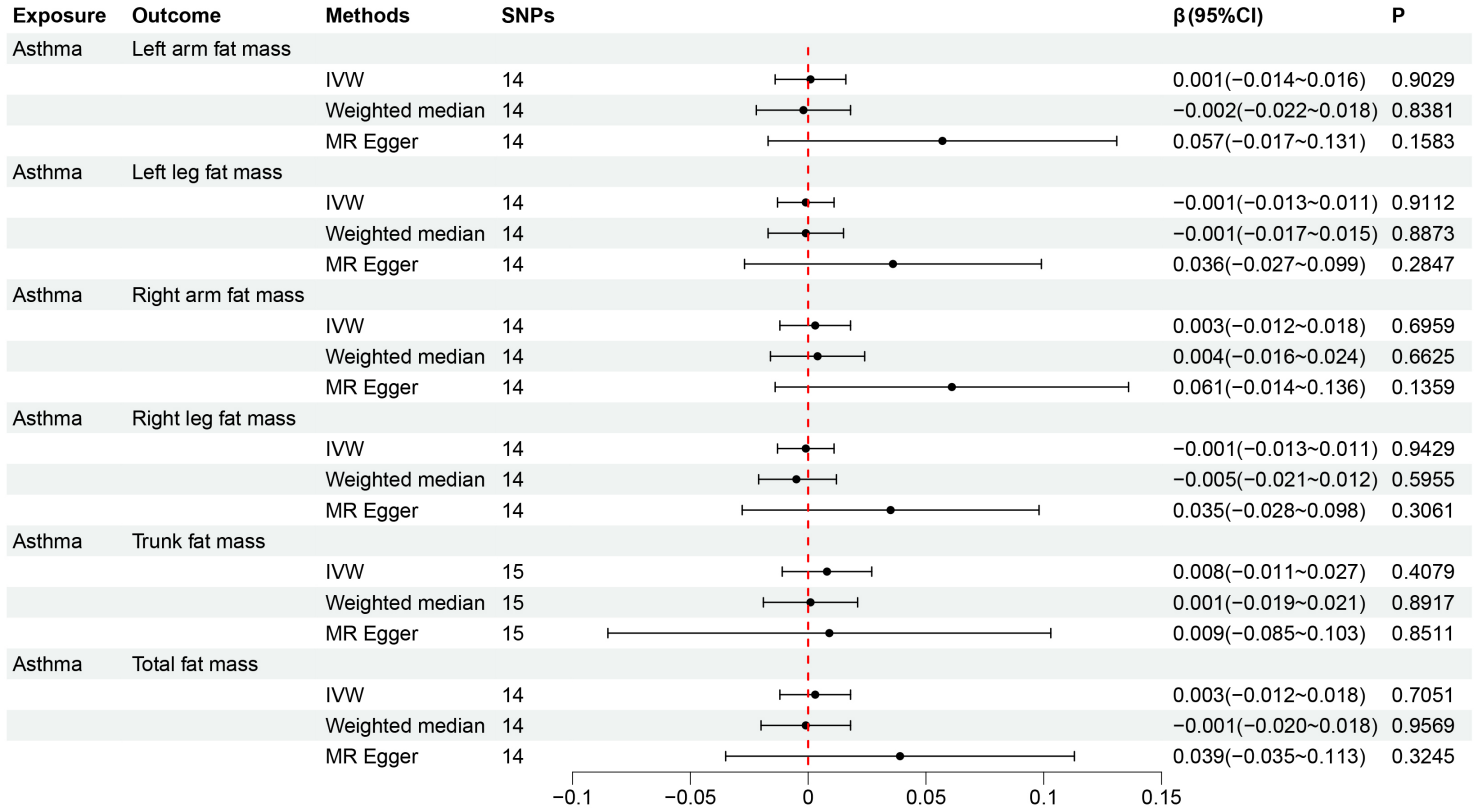
Reverse MR analysis results for the causal effect of adult asthma on six fat mass indicators. MR, Mendelian randomization; SNPs, single nucleotide polymorphisms; IVW, inverse-variance weighted; CI, confidence interval.

In the reverse MR analysis of adult asthma on the six fat mass indicators, 14 SNPs were included for each of left arm fat mass, left leg fat mass, right arm fat mass, right leg fat mass, and total fat mass, while 15 SNPs were included for trunk fat mass. All SNPs had F-statistics > 10 (Supplementary Tables S8–S13). The results of the IVW method showed no causal effect of adult asthma on six fat mass indicators (all *p* > 0.05) (Figure 5). The estimated causal effects of each SNP on six fat mass indicators are shown in Supplementary Figures S15–S20. The results of both the MR-PRESSO global test and MR-Egger regression showed that SNPs did not have horizontal pleiotropy (Table 3; Supplementary Figure S21). In analyzing the causal effect of adult asthma on trunk fat mass, the heterogeneity test had *I*^2^ > 25%, so the random effects model of IVW was used. For the other fat mass indicators, there was no heterogeneity observed (all *p* > 0.05, *I*^2^ < 25%) (Table 3). The results of the leave-one-out analysis and the funnel plot were shown in Supplementary Figures S22–S28.

## 4 Discussion

We used a bidirectional MR analysis in conjunction with cross-sectional data from the NHANES 2011–2018 to investigate the relationship between body fat distribution measures and adult asthma. The results indicated that body fat distribution measurements were associated with an increased risk of adult asthma, and exhibited a unidirectional causal relationship in MR analysis.

Numerous studies have examined the link between obesity and asthma. In order to evaluate the association between adult asthma and obesity, Brumpton et al. (7) recruited 23,245 people from the Norwegian population for their prospective research. They defined general obesity as a BMI ≥30 kg/m^2^. After 11 years of follow-up, they found that general obesity increased the risk of asthma in both adult men (OR = 1.84, *p* < 0.05) and women (OR = 1.96, *p* < 0.05). A multicenter clinical study by Liu et al. (12) included 11,868 US adults and found that both underweight (BMI < 18.5 kg/m^2^) and obesity (BMI ≥ 30 kg/m^2^) were risk factors for asthma. Although BMI is widely used and simple to measure, it has limitations. A study by Tomiyama et al. (26) included 40,420 US adults, who were stratified by BMI to investigate cardiac metabolic conditions across different groups. They found that 50% of the overweight individuals and 29% of those classified as obese were metabolically healthy, whereas over 30% of those with normal weight had unhealthy cardiac metabolism. Lahav et al. (27), after analyzing medical examination data from approximately 3,000 Israeli adult men and women, found that body fat percentage was a more reliable predictor of overall individual health and cardiac metabolic risk than BMI. Prillaman (28) pointed out that obesity should be redefined. He believed that BMI only considered height and weight, yet neglected other factors such as body fat, which might potentially have a more significant influence on one’s health. In light of this, in our study, we utilized body fat distribution measurements to evaluate the correlation between obesity and adult asthma.

Following the adjustment for 11 covariates, including age and gender, the weighted logistic regression analyses in the cross-sectional study from NHANES 2011–2018 revealed that fat mass in the left arm, left leg, right arm, right leg, trunk, and overall body was linked to an increased risk of adult asthma. Furthermore, to assess the trend of asthma risk increasing with fat mass, we employed RCS for analysis. A U-shaped connection between BMI and asthma risk was found in a study by Sun et al. (11). However, our RCS analysis revealed a J-shaped relationship between six fat mass indicators and asthma risk, indicating that below a certain threshold, changes in fat mass did not affect asthma risk, but beyond this threshold, asthma risk increased with fat mass. Indeed, this J-shaped relationship simplifies the association between fat mass and asthma compared to BMI, which requires concern both for being too high and too low. However, cross-sectional studies can only establish the correlation between body fat distribution measurements and adult asthma, and cannot determine the causal relationship or direction between the two (29). Therefore, we further used a bidirectional MR study to investigate their causal link and direction. In the forward MR analysis evaluating the causal effect of fat mass on adult asthma, with the IVW method as the primary approach, we found that all six fat mass indicators increased the risk of asthma. Additionally, sensitivity analysis supported the reliability of the MR analysis. In the reverse MR analysis, the IVW method’s findings demonstrated that none of the six fat mass indicators were causally impacted by adult asthma.

Several reasons may account for the correlation between high fat mass and a higher incidence of asthma in adults. One possible reason is that the buildup of adipose tissue in the chest wall and abdominal wall may reduce the compliance of the respiratory system, thus affecting the circulation of gasses and resulting in reduced functional residual capacity (FRC) (30). Reduced FRC weakens the mutual support between the airways and the parenchyma, which in turn causes the collapse and narrowing of the airways (31). Additionally, a longitudinal study from Korea found that fat increased airway hyperresponsiveness in adults, which is an important feature of asthma (32). Another possible reason is that fat increases the risk of asthma by promoting inflammation. Large numbers of macrophages were found in the adipose tissue of obese individuals by Weisberg et al. (33). According to Periyalil et al. (34), the majority of the macrophages in the obese asthma patients’ adipose tissue were pro-inflammatory. Leptin is a hormone released by fat cells that has pro-inflammatory and immune-regulating effects (35). Sideleva et al. (36) found that the expression of leptin was increased in obese asthma patients, suggesting it might be a significant mediator in the occurrence of airway diseases in obese individuals. Further research is needed to validate our hypotheses regarding how fat increases the risk of asthma.

Our study’s strength is that we used cross-sectional studies in conjunction with MR analysis to assess the association between body fat distribution measurements and adult asthma risk. We considered both epidemiology and genetics, and using these two methods together greatly reduced the effects of reverse causation and confounding variables. The consistency of conclusions from both approaches also makes the research findings more reliable. Furthermore, the bidirectional MR analysis we employed not only established causality but also determined the direction of the causal relationships. However, our study is subject to certain limitations. Firstly, in cross-sectional studies, asthma diagnoses are often based on survey questionnaires, which can introduce bias. Secondly, in contrast to the NHANES, which mostly surveys Americans, MR covers people all around Europe. Disparities in race might influence the results. Thirdly, the impact of body fat mass on the risk of adult asthma across different genders was not examined due to data constraints. Finally, we did not go into the specific mechanisms via which excess fat increases the risk of adult asthma.

## 5 Conclusion

In conclusion, the results from both cross-sectional studies and MR analysis support a correlation between body fat distribution and the risk of adult asthma, with higher fat mass potentially increasing the risk of adult asthma. However, reverse MR did not find evidence that asthma could increase fat mass. Our study provides a new perspective on the prevention and treatment of asthma in adults, and more research is still needed to validate our findings in the future.

## Data availability statement

The datasets presented in this study can be found in online repositories. The names of the repository/repositories and accession number(s) can be found in the article/Supplementary material.

## Ethics statement

The studies involving humans were approved by National Center for Health Statistics. The studies were conducted in accordance with the local legislation and institutional requirements. The ethics committee/institutional review board waived the requirement of written informed consent for participation from the participants or the participants’ legal guardians/next of kin because Data were obtained from public databases.

## Author contributions

KW: Conceptualization, Methodology, Writing – original draft. ZC: Data curation, Methodology, Writing – original draft. ZW: Supervision, Writing – review & editing, Software. LH: Data curation, Methodology, Writing – original draft. LG: Conceptualization, Funding acquisition, Supervision, Writing – review & editing.

## Footnotes

^1^ https://www.cdc.gov/nchs/nhanes/index.htm

## References

[1] Papi A Brightling C Pedersen SE Reddel HK. Asthma. Lancet. (2018) 391:783–800. doi: 10.1016/s0140-6736(17)33311-1 29273246

[2] GBD 2019 Chronic Respiratory Diseases Collaborators. Global burden of chronic respiratory diseases and risk factors, 1990-2019: an update from the global burden of disease study 2019. EClinicalMedicine. (2023) 59:101936. doi: 10.1016/j.eclinm.2023.101936, PMID: 37229504 PMC7614570

[3] Kaplan A Szefler SJ Halpin DMG. Impact of comorbid conditions on asthmatic adults and children. NPJ Prim Care Respir Med. (2020) 30:36. doi: 10.1038/s41533-020-00194-9, PMID: 32820164 PMC7441401

[4] Lommatzsch M Buhl R Korn S. The treatment of mild and moderate asthma in adults. Dtsch Arztebl Int. (2020) 117:434–44. doi: 10.3238/arztebl.2020.0434, PMID: 32885783 PMC7490458

[5] Blüher M. Obesity: global epidemiology and pathogenesis. Nat Rev Endocrinol. (2019) 15:288–98. doi: 10.1038/s41574-019-0176-8 30814686

[6] Murugan AT Sharma G. Obesity and respiratory diseases. Chron Respir Dis. (2008) 5:233–42. doi: 10.1177/1479972308096978 19029235

[7] Brumpton B Langhammer A Romundstad P Chen Y Mai XM. General and abdominal obesity and incident asthma in adults: the HUNT study. Eur Respir J. (2013) 41:323–9. doi: 10.1183/09031936.00012112, PMID: 22653771

[8] Ma J Xiao L Knowles SB. Obesity, insulin resistance and the prevalence of atopy and asthma in US adults. Allergy. (2010) 65:1455–63. doi: 10.1111/j.1398-9995.2010.02402.x, PMID: 20456316

[9] Beuther DA Sutherland ER. Overweight, obesity, and incident asthma: a meta-analysis of prospective epidemiologic studies. Am J Respir Crit Care Med. (2007) 175:661–6. doi: 10.1164/rccm.200611-1717OC, PMID: 17234901 PMC1899288

[10] Bray GA Beyond BMI. Beyond BMI. Nutrients. (2023) 15:2254. doi: 10.3390/nu15102254, PMID: 37242136 PMC10223432

[11] Sun Y Zhang Y Liu X Liu Y Wu F Liu X. Association between body mass index and respiratory symptoms in US adults: a national cross-sectional study. Sci Rep. (2024) 14:940. doi: 10.1038/s41598-024-51637-z, PMID: 38195711 PMC10776771

[12] Liu Y Pleasants RA Croft JB Lugogo N Ohar J Heidari K. Body mass index, respiratory conditions, asthma, and chronic obstructive pulmonary disease. Respir Med. (2015) 109:851–9. doi: 10.1016/j.rmed.2015.05.006, PMID: 26006753 PMC4487766

[13] Kang PS Neeland IJ. Body fat distribution, diabetes mellitus, and cardiovascular disease: an update. Curr Cardiol Rep. (2023) 25:1555–64. doi: 10.1007/s11886-023-01969-5, PMID: 37792133

[14] Zhang L Xia Z Li Z Zhang J Wang K Wang W. Influence of body fat tissue on outcomes in patients undergoing hepatectomy or liver transplantation. Int J Surg. (2024). doi: 10.1097/js9.0000000000001864 [Epub ahead of print]. PMC11745742 38920322

[15] Ahluwalia N Dwyer J Terry A Moshfegh A Johnson C. Update on NHANES dietary data: focus on collection, release, analytical considerations, and uses to inform public policy. Adv Nutr. (2016) 7:121–34. doi: 10.3945/an.115.009258, PMID: 26773020 PMC4717880

[16] Emdin CA Khera AV Kathiresan S. Mendelian randomization. JAMA. (2017) 318:1925–6. doi: 10.1001/jama.2017.17219 29164242

[17] Lin Z Huang J Xie S Zheng Z Tang K Li S. The association between insulin use and asthma: an epidemiological observational analysis and Mendelian randomization study. Lung. (2023) 201:189–99. doi: 10.1007/s00408-023-00611-z, PMID: 36971839

[18] Odebeatu CC Taylor T Fleming LE Osborne NJ. Phthalates and asthma in children and adults: US NHANES 2007-2012. Environ Sci Pollut Res Int. (2019) 26:28256–69. doi: 10.1007/s11356-019-06003-2, PMID: 31368075 PMC6791917

[19] Lin S Su X Chen L Cai Z. Association of dietary inflammatory index with sarcopenia in asthmatic patients: a cross-sectional study. Front Nutr. (2023) 10:1215688. doi: 10.3389/fnut.2023.1215688, PMID: 37720383 PMC10501140

[20] Burgess S Thompson SG. Avoiding bias from weak instruments in Mendelian randomization studies. Int J Epidemiol. (2011) 40:755–64. doi: 10.1093/ije/dyr036, PMID: 21414999

[21] Papadimitriou N Dimou N Tsilidis KK Banbury B Martin RM Lewis SJ. Physical activity and risks of breast and colorectal cancer: a Mendelian randomisation analysis. Nat Commun. (2020) 11:597. doi: 10.1038/s41467-020-14389-8, PMID: 32001714 PMC6992637

[22] Hartwig FP Davey Smith G Bowden J. Robust inference in summary data Mendelian randomization via the zero modal pleiotropy assumption. Int J Epidemiol. (2017) 46:1985–98. doi: 10.1093/ije/dyx102, PMID: 29040600 PMC5837715

[23] Bowden J Del Greco MF Minelli C Davey Smith G Sheehan N Thompson J. A framework for the investigation of pleiotropy in two-sample summary data Mendelian randomization. Stat Med. (2017) 36:1783–802. doi: 10.1002/sim.7221, PMID: 28114746 PMC5434863

[24] Verbanck M Chen CY Neale B Do R. Detection of widespread horizontal pleiotropy in causal relationships inferred from Mendelian randomization between complex traits and diseases. Nat Genet. (2018) 50:693–8. doi: 10.1038/s41588-018-0099-7, PMID: 29686387 PMC6083837

[25] Bowden J Davey Smith G Burgess S. Mendelian randomization with invalid instruments: effect estimation and bias detection through egger regression. Int J Epidemiol. (2015) 44:512–25. doi: 10.1093/ije/dyv080, PMID: 26050253 PMC4469799

[26] Tomiyama AJ Hunger JM Nguyen-Cuu J Wells C. Misclassification of cardiometabolic health when using body mass index categories in NHANES 2005-2012. Int J Obes. (2016) 40:883–6. doi: 10.1038/ijo.2016.17, PMID: 26841729

[27] Lahav Y Kfir A Gepner Y. The paradox of obesity with normal weight; a cross-sectional study. Front Nutr. (2023) 10:1173488. doi: 10.3389/fnut.2023.1173488, PMID: 37360304 PMC10287971

[28] Prillaman M. Why BMI is flawed - and how to redefine obesity. Nature. (2023) 622:232–3. doi: 10.1038/d41586-023-03143-x 37903933

[29] Grimes DA Schulz KF. Bias and causal associations in observational research. Lancet. (2002) 359:248–52. doi: 10.1016/s0140-6736(02)07451-2 11812579

[30] Littleton SW. Impact of obesity on respiratory function. Respirology. (2012) 17:43–9. doi: 10.1111/j.1440-1843.2011.02096.x 22040049

[31] Salome CM King GG Berend N. Physiology of obesity and effects on lung function. J Appl Physiol (1985). (2010) 108:206–11. doi: 10.1152/japplphysiol.00694.2009 19875713

[32] Shim JS Kim SS Lee SH Kim MH Cho YJ Park HW. Fat mass index and airway hyperresponsiveness in Korean adults. Postgrad Med. (2023) 135:480–5. doi: 10.1080/00325481.2023.2188000, PMID: 36879538

[33] Weisberg SP Mccann D Desai M Rosenbaum M Leibel RL Ferrante AW Jr. Obesity is associated with macrophage accumulation in adipose tissue. J Clin Invest. (2003) 112:1796–808. doi: 10.1172/jci19246 14679176 PMC296995

[34] Periyalil HA Wood LG Wright TA Karihaloo C Starkey MR Miu AS. Obese asthmatics are characterized by altered adipose tissue macrophage activation. Clin Exp Allergy. (2018) 48:641–9. doi: 10.1111/cea.13109, PMID: 29383778

[35] Otero M Lago R Gomez R Dieguez C Lago F Gómez-Reino J. Towards a pro-inflammatory and immunomodulatory emerging role of leptin. Rheumatology (Oxford). (2006) 45:944–50. doi: 10.1093/rheumatology/kel157, PMID: 16720637

[36] Sideleva O Suratt BT Black KE Tharp WG Pratley RE Forgione P. Obesity and asthma: an inflammatory disease of adipose tissue not the airway. Am J Respir Crit Care Med. (2012) 186:598–605. doi: 10.1164/rccm.201203-0573OC 22837379 PMC3480522

