# Supplementary material for: Association between body fat distribution and asthma in adults: results from the cross-sectional and bidirectional Mendelian randomization study
Source: Front Nutr. 2024 Jul 22;11:1432973. doi: 10.3389/fnut.2024.1432973 (PMC11299241; doi:10.3389/fnut.2024.1432973)
Supplement: Supplementary file 1 [file Table_1.docx]

**Supplementary Table 1: Data sources of exposure and outcome included in our study.**

| Trait | Population | Sample size | Link |
| --- | --- | --- | --- |
| Asthma | European | 156,078 | https://gwas.mrcieu.ac.uk/datasets/finn-b-J10_ASTHMA/ |
| Left arm fat mass | European | 331,164 | https://gwas.mrcieu.ac.uk/datasets/ukb-a-287/ |
| Left leg fat mass | European | 331,275 | https://gwas.mrcieu.ac.uk/datasets/ukb-a-279/ |
| Right arm fat mass | European | 331,226 | https://gwas.mrcieu.ac.uk/datasets/ukb-a-283/ |
| Right leg fat mass | European | 331,293 | https://gwas.mrcieu.ac.uk/datasets/ukb-a-275/ |
| Trunk fat mass | European | 331,093 | https://gwas.mrcieu.ac.uk/datasets/ukb-a-291/ |
| Total fat mass | European | 330,762 | https://gwas.mrcieu.ac.uk/datasets/ukb-a-265/ |

**Supplementary Table 2: Characteristics of 232 SNPs used as instrumental variables for the effect of Left arm fat mass on asthma.**

|  | SNP | effect_allele.exposure | other_allele.exposure | EAF.exposure | effect | SE | *P*-value | F-Statistic |
| --- | --- | --- | --- | --- | --- | --- | --- | --- |
| 1 | rs10100245 | A | G | 0.56644 | 0.02084 | 0.00240 | 4.00E-18 | 75.33 |
| 2 | rs10132436 | C | A | 0.27626 | -0.01493 | 0.00268 | 2.60E-08 | 30.98 |
| 3 | rs1013293 | A | G | 0.43334 | -0.01954 | 0.00241 | 5.37E-16 | 65.66 |
| 4 | rs10187101 | T | C | 0.35982 | -0.01530 | 0.00248 | 6.96E-10 | 38.03 |
| 5 | rs10237317 | G | A | 0.41697 | 0.01587 | 0.00242 | 5.90E-11 | 42.86 |
| 6 | rs10259620 | G | A | 0.78717 | -0.01698 | 0.00293 | 6.61E-09 | 33.65 |
| 7 | rs10269774 | A | G | 0.32354 | 0.01563 | 0.00255 | 8.23E-10 | 37.71 |
| 8 | rs10404726 | T | C | 0.46678 | -0.01837 | 0.00238 | 1.34E-14 | 59.33 |
| 9 | rs10756798 | T | C | 0.64503 | -0.01781 | 0.00249 | 8.26E-13 | 51.22 |
| 10 | rs10760724 | C | T | 0.47394 | 0.01532 | 0.00238 | 1.27E-10 | 41.36 |
| 11 | rs10787738 | T | C | 0.25330 | 0.01890 | 0.00277 | 9.68E-12 | 46.39 |
| 12 | rs10820852 | A | C | 0.27434 | -0.01622 | 0.00267 | 1.29E-09 | 36.84 |
| 13 | rs10938398 | A | G | 0.43334 | 0.02804 | 0.00241 | 2.56E-31 | 135.53 |
| 14 | rs10947793 | G | A | 0.37360 | -0.01435 | 0.00247 | 6.67E-09 | 33.63 |
| 15 | rs10954772 | C | T | 0.68571 | -0.01730 | 0.00258 | 2.02E-11 | 44.95 |
| 16 | rs11030119 | A | G | 0.30881 | 0.03229 | 0.00258 | 5.15E-36 | 157.02 |
| 17 | rs11042030 | C | T | 0.27401 | -0.01861 | 0.00267 | 3.26E-12 | 48.53 |
| 18 | rs11135450 | G | A | 0.66888 | 0.01455 | 0.00254 | 9.76E-09 | 32.89 |
| 19 | rs11150745 | G | A | 0.31949 | -0.01944 | 0.00256 | 2.85E-14 | 57.84 |
| 20 | rs11152135 | C | G | 0.76339 | 0.01600 | 0.00281 | 1.26E-08 | 32.40 |
| 21 | rs11165643 | T | C | 0.59231 | 0.02009 | 0.00242 | 1.09E-16 | 68.80 |
| 22 | rs11205617 | A | G | 0.31713 | 0.02046 | 0.00255 | 1.05E-15 | 64.34 |
| 23 | rs112094960 | C | A | 0.21491 | 0.01588 | 0.00291 | 5.00E-08 | 29.72 |
| 24 | rs1127100 | C | T | 0.64824 | 0.01624 | 0.00250 | 8.06E-11 | 42.25 |
| 25 | rs113182412 | A | G | 0.16592 | -0.01808 | 0.00325 | 2.75E-08 | 30.88 |
| 26 | rs113230003 | A | G | 0.26019 | -0.01968 | 0.00273 | 6.08E-13 | 51.83 |
| 27 | rs113603865 | T | C | 0.21243 | 0.02034 | 0.00293 | 3.80E-12 | 48.23 |
| 28 | rs113866544 | C | T | 0.06768 | 0.03423 | 0.00474 | 5.33E-13 | 52.08 |
| 29 | rs11515071 | T | C | 0.36514 | -0.02355 | 0.00248 | 2.46E-21 | 89.95 |
| 30 | rs11538 | G | A | 0.17213 | 0.01731 | 0.00315 | 3.95E-08 | 30.18 |
| 31 | rs11642015 | T | C | 0.40236 | 0.06484 | 0.00243 | 2.75E-157 | 714.73 |
| 32 | rs11742930 | T | C | 0.56643 | 0.01503 | 0.00241 | 4.17E-10 | 39.04 |
| 33 | rs11766945 | A | G | 0.20101 | -0.02073 | 0.00299 | 4.07E-12 | 48.09 |
| 34 | rs11782341 | G | A | 0.18851 | 0.01817 | 0.00306 | 3.03E-09 | 35.17 |
| 35 | rs11786089 | G | A | 0.45685 | 0.01370 | 0.00239 | 1.04E-08 | 32.77 |
| 36 | rs1182143 | C | T | 0.29630 | -0.01976 | 0.00261 | 3.46E-14 | 57.46 |
| 37 | rs11882409 | A | C | 0.28636 | 0.01881 | 0.00268 | 2.08E-12 | 49.41 |
| 38 | rs11920781 | C | T | 0.36517 | 0.01651 | 0.00247 | 2.56E-11 | 44.49 |
| 39 | rs12001437 | C | T | 0.36664 | 0.01378 | 0.00247 | 2.37E-08 | 31.17 |
| 40 | rs12072739 | G | A | 0.22460 | 0.01747 | 0.00286 | 9.38E-10 | 37.45 |
| 41 | rs12121667 | A | G | 0.61755 | 0.01392 | 0.00245 | 1.33E-08 | 32.29 |
| 42 | rs12144626 | C | T | 0.58182 | -0.01715 | 0.00242 | 1.42E-12 | 50.16 |
| 43 | rs12254441 | T | C | 0.37284 | -0.01520 | 0.00255 | 2.39E-09 | 35.63 |
| 44 | rs12339822 | G | A | 0.54320 | 0.01607 | 0.00240 | 2.10E-11 | 44.88 |
| 45 | rs12367809 | T | C | 0.36828 | 0.02886 | 0.00248 | 2.36E-31 | 135.70 |
| 46 | rs12506689 | A | G | 0.36614 | 0.01654 | 0.00247 | 2.26E-11 | 44.74 |
| 47 | rs12578952 | G | A | 0.25170 | -0.01727 | 0.00274 | 3.01E-10 | 39.67 |
| 48 | rs12619178 | T | C | 0.40292 | -0.01714 | 0.00243 | 1.60E-12 | 49.93 |
| 49 | rs12619626 | G | A | 0.24874 | -0.01502 | 0.00275 | 4.91E-08 | 29.76 |
| 50 | rs12692738 | C | T | 0.22531 | 0.01960 | 0.00287 | 8.38E-12 | 46.68 |
| 51 | rs1286138 | G | T | 0.67344 | 0.01555 | 0.00254 | 9.39E-10 | 37.45 |
| 52 | rs12877270 | A | G | 0.43845 | 0.01559 | 0.00242 | 1.12E-10 | 41.60 |
| 53 | rs12885458 | G | T | 0.50998 | -0.01479 | 0.00238 | 5.28E-10 | 38.57 |
| 54 | rs12887636 | G | T | 0.34740 | -0.01550 | 0.00252 | 7.28E-10 | 37.95 |
| 55 | rs1296328 | C | A | 0.55991 | -0.01731 | 0.00241 | 6.73E-13 | 51.62 |
| 56 | rs12992672 | A | G | 0.82864 | 0.04589 | 0.00315 | 5.70E-48 | 211.82 |
| 57 | rs13062093 | G | T | 0.36644 | 0.01615 | 0.00247 | 5.95E-11 | 42.84 |
| 58 | rs13076052 | G | C | 0.27704 | 0.01462 | 0.00268 | 4.69E-08 | 29.84 |
| 59 | rs13135092 | G | A | 0.08323 | 0.04200 | 0.00434 | 3.42E-22 | 93.85 |
| 60 | rs13174863 | G | A | 0.14821 | 0.02592 | 0.00337 | 1.47E-14 | 59.14 |
| 61 | rs1335055 | A | G | 0.65578 | -0.01525 | 0.00253 | 1.66E-09 | 36.34 |
| 62 | rs13427822 | G | A | 0.27205 | -0.01649 | 0.00270 | 1.08E-09 | 37.17 |
| 63 | rs1406778 | C | T | 0.19754 | 0.02317 | 0.00299 | 8.62E-15 | 60.19 |
| 64 | rs1441264 | A | G | 0.59187 | 0.01921 | 0.00247 | 8.03E-15 | 60.33 |
| 65 | rs1446585 | G | A | 0.22801 | -0.01694 | 0.00285 | 2.77E-09 | 35.34 |
| 66 | rs1459189 | T | C | 0.52003 | -0.01377 | 0.00240 | 1.03E-08 | 32.78 |
| 67 | rs1470749 | T | G | 0.51060 | -0.01370 | 0.00238 | 9.04E-09 | 33.04 |
| 68 | rs1471338 | G | A | 0.29080 | 0.01447 | 0.00263 | 3.54E-08 | 30.39 |
| 69 | rs147730268 | T | G | 0.09053 | -0.04210 | 0.00429 | 9.65E-23 | 96.36 |
| 70 | rs1503526 | C | T | 0.47918 | 0.01683 | 0.00238 | 1.62E-12 | 49.90 |
| 71 | rs1568488 | C | G | 0.59563 | 0.01589 | 0.00245 | 8.20E-11 | 42.21 |
| 72 | rs1609303 | A | T | 0.63122 | 0.01703 | 0.00248 | 6.18E-12 | 47.27 |
| 73 | rs17024393 | C | T | 0.02580 | 0.06893 | 0.00753 | 5.66E-20 | 83.75 |
| 74 | rs17149254 | C | T | 0.80856 | -0.02058 | 0.00309 | 2.60E-11 | 44.46 |
| 75 | rs17311369 | T | C | 0.31951 | -0.01563 | 0.00257 | 1.14E-09 | 37.07 |
| 76 | rs17627049 | A | C | 0.27425 | 0.01652 | 0.00267 | 5.89E-10 | 38.36 |
| 77 | rs17716502 | T | C | 0.20724 | -0.02425 | 0.00296 | 2.43E-16 | 67.22 |
| 78 | rs17770336 | T | C | 0.32382 | 0.02324 | 0.00254 | 5.64E-20 | 83.75 |
| 79 | rs1782508 | G | C | 0.65633 | -0.01584 | 0.00250 | 2.43E-10 | 40.09 |
| 80 | rs1840126 | C | A | 0.56206 | -0.01310 | 0.00240 | 4.74E-08 | 29.82 |
| 81 | rs1881505 | C | T | 0.94210 | -0.02815 | 0.00515 | 4.47E-08 | 29.94 |
| 82 | rs1899689 | T | C | 0.38732 | 0.01503 | 0.00244 | 7.84E-10 | 37.80 |
| 83 | rs1928496 | T | C | 0.74239 | 0.01644 | 0.00272 | 1.47E-09 | 36.57 |
| 84 | rs1964599 | T | C | 0.34371 | -0.01589 | 0.00256 | 5.37E-10 | 38.54 |
| 85 | rs2016469 | A | G | 0.37370 | 0.01455 | 0.00248 | 4.45E-09 | 34.42 |
| 86 | rs2098870 | A | T | 0.30721 | 0.01449 | 0.00258 | 1.93E-08 | 31.57 |
| 87 | rs2102278 | G | A | 0.32208 | 0.01582 | 0.00255 | 5.72E-10 | 38.42 |
| 88 | rs2172131 | C | T | 0.58064 | -0.01665 | 0.00241 | 5.18E-12 | 47.62 |
| 89 | rs2239647 | C | A | 0.55179 | -0.01933 | 0.00239 | 7.00E-16 | 65.14 |
| 90 | rs2289379 | T | C | 0.39497 | -0.01703 | 0.00244 | 3.15E-12 | 48.60 |
| 91 | rs2292238 | C | A | 0.40867 | -0.01494 | 0.00243 | 7.28E-10 | 37.95 |
| 92 | rs2306593 | T | C | 0.48852 | -0.01757 | 0.00238 | 1.73E-13 | 54.30 |
| 93 | rs2307111 | C | T | 0.39318 | -0.02788 | 0.00244 | 2.96E-30 | 130.67 |
| 94 | rs2318543 | G | A | 0.78250 | -0.01837 | 0.00289 | 2.21E-10 | 40.28 |
| 95 | rs2370982 | T | C | 0.21534 | 0.02648 | 0.00292 | 1.15E-19 | 82.34 |
| 96 | rs2374947 | G | A | 0.76098 | 0.01665 | 0.00280 | 2.87E-09 | 35.27 |
| 97 | rs2425024 | C | A | 0.32374 | 0.01583 | 0.00254 | 4.74E-10 | 38.78 |
| 98 | rs2439823 | G | A | 0.54771 | 0.02007 | 0.00240 | 5.61E-17 | 70.12 |
| 99 | rs2444253 | G | C | 0.39255 | -0.01332 | 0.00244 | 4.83E-08 | 29.79 |
| 100 | rs245775 | G | A | 0.72883 | 0.02093 | 0.00268 | 5.60E-15 | 61.04 |
| 101 | rs2470619 | G | A | 0.46523 | -0.01349 | 0.00239 | 1.63E-08 | 31.90 |
| 102 | rs2479958 | G | A | 0.51515 | -0.01613 | 0.00241 | 2.10E-11 | 44.88 |
| 103 | rs2494196 | A | C | 0.28766 | 0.01793 | 0.00263 | 9.16E-12 | 46.50 |
| 104 | rs2499468 | A | C | 0.65274 | 0.01558 | 0.00250 | 4.91E-10 | 38.72 |
| 105 | rs252749 | A | G | 0.24657 | -0.01921 | 0.00276 | 3.16E-12 | 48.59 |
| 106 | rs2678204 | G | T | 0.34181 | 0.02510 | 0.00251 | 1.66E-23 | 99.85 |
| 107 | rs273505 | C | T | 0.41991 | 0.02031 | 0.00241 | 3.78E-17 | 70.90 |
| 108 | rs28366156 | C | T | 0.13136 | -0.02734 | 0.00352 | 8.53E-15 | 60.21 |
| 109 | rs28414958 | A | G | 0.06987 | 0.02637 | 0.00468 | 1.77E-08 | 31.73 |
| 110 | rs28457808 | G | C | 0.16319 | -0.01977 | 0.00323 | 9.02E-10 | 37.53 |
| 111 | rs28489620 | A | G | 0.29098 | -0.01661 | 0.00264 | 3.11E-10 | 39.61 |
| 112 | rs2861685 | C | T | 0.41137 | -0.01617 | 0.00242 | 2.15E-11 | 44.83 |
| 113 | rs286818 | A | T | 0.17025 | -0.02286 | 0.00318 | 6.11E-13 | 51.81 |
| 114 | rs3134404 | T | C | 0.27839 | 0.01517 | 0.00267 | 1.28E-08 | 32.36 |
| 115 | rs34045288 | T | C | 0.33590 | 0.02388 | 0.00252 | 2.85E-21 | 89.66 |
| 116 | rs34045894 | A | G | 0.15739 | 0.01816 | 0.00327 | 2.72E-08 | 30.90 |
| 117 | rs34292685 | T | C | 0.16209 | -0.02003 | 0.00322 | 5.18E-10 | 38.61 |
| 118 | rs34517439 | A | C | 0.12577 | 0.04120 | 0.00365 | 1.56E-29 | 127.37 |
| 119 | rs34769775 | T | C | 0.29786 | -0.01709 | 0.00260 | 4.89E-11 | 43.22 |
| 120 | rs35537311 | T | C | 0.38988 | -0.01367 | 0.00244 | 2.20E-08 | 31.31 |
| 121 | rs35851183 | G | A | 0.35778 | 0.01613 | 0.00249 | 8.65E-11 | 42.11 |
| 122 | rs3729793 | G | C | 0.09388 | 0.02238 | 0.00409 | 4.48E-08 | 29.93 |
| 123 | rs3770821 | C | T | 0.33132 | 0.01486 | 0.00254 | 5.22E-09 | 34.11 |
| 124 | rs3802858 | C | T | 0.42749 | -0.01318 | 0.00241 | 4.40E-08 | 29.97 |
| 125 | rs3803286 | G | A | 0.66638 | -0.01878 | 0.00252 | 9.53E-14 | 55.47 |
| 126 | rs3810291 | A | G | 0.67748 | 0.02589 | 0.00254 | 2.59E-24 | 103.52 |
| 127 | rs3826408 | T | C | 0.45721 | 0.01457 | 0.00239 | 1.06E-09 | 37.21 |
| 128 | rs4072917 | A | G | 0.47542 | 0.01408 | 0.00239 | 3.96E-09 | 34.64 |
| 129 | rs4073582 | A | G | 0.35979 | -0.01648 | 0.00248 | 3.09E-11 | 44.12 |
| 130 | rs4246657 | T | C | 0.34091 | 0.01639 | 0.00251 | 6.73E-11 | 42.60 |
| 131 | rs4482463 | A | C | 0.92418 | -0.03310 | 0.00451 | 2.05E-13 | 53.96 |
| 132 | rs4502882 | T | C | 0.65689 | -0.01590 | 0.00251 | 2.21E-10 | 40.27 |
| 133 | rs4503172 | T | C | 0.60782 | -0.01362 | 0.00244 | 2.34E-08 | 31.19 |
| 134 | rs4648450 | A | C | 0.46613 | -0.01350 | 0.00240 | 1.86E-08 | 31.63 |
| 135 | rs4657796 | C | T | 0.37656 | -0.01484 | 0.00246 | 1.66E-09 | 36.34 |
| 136 | rs4673617 | T | C | 0.27164 | 0.01779 | 0.00268 | 3.46E-11 | 43.90 |
| 137 | rs4725984 | C | T | 0.64145 | 0.01663 | 0.00250 | 2.79E-11 | 44.32 |
| 138 | rs4740383 | A | G | 0.40351 | 0.01338 | 0.00243 | 3.79E-08 | 30.25 |
| 139 | rs4757142 | A | G | 0.61014 | 0.01609 | 0.00246 | 5.60E-11 | 42.96 |
| 140 | rs4776970 | T | A | 0.35707 | -0.02417 | 0.00248 | 2.19E-22 | 94.74 |
| 141 | rs4777541 | T | C | 0.76504 | 0.01944 | 0.00282 | 5.47E-12 | 47.51 |
| 142 | rs4886039 | G | A | 0.17143 | -0.02052 | 0.00316 | 8.21E-11 | 42.21 |
| 143 | rs4921301 | T | C | 0.20982 | -0.01722 | 0.00296 | 5.75E-09 | 33.92 |
| 144 | rs4958702 | C | T | 0.57368 | -0.01579 | 0.00240 | 5.19E-11 | 43.11 |
| 145 | rs4962424 | A | T | 0.32715 | 0.01411 | 0.00254 | 2.68E-08 | 30.93 |
| 146 | rs541577 | G | A | 0.62126 | -0.01439 | 0.00246 | 5.20E-09 | 34.12 |
| 147 | rs543874 | G | A | 0.20781 | 0.04526 | 0.00293 | 8.26E-54 | 238.61 |
| 148 | rs55637757 | T | C | 0.13344 | -0.02110 | 0.00356 | 3.03E-09 | 35.17 |
| 149 | rs55655049 | T | C | 0.23647 | -0.01530 | 0.00281 | 4.90E-08 | 29.76 |
| 150 | rs55726687 | A | G | 0.21114 | 0.02264 | 0.00292 | 8.04E-15 | 60.33 |
| 151 | rs55966114 | T | C | 0.19304 | 0.01794 | 0.00302 | 2.92E-09 | 35.24 |
| 152 | rs56803094 | G | A | 0.22711 | -0.01868 | 0.00285 | 5.36E-11 | 43.04 |
| 153 | rs57636386 | C | T | 0.08342 | -0.04106 | 0.00431 | 1.72E-21 | 90.66 |
| 154 | rs58084604 | T | C | 0.23441 | 0.05049 | 0.00281 | 2.32E-72 | 323.81 |
| 155 | rs6029180 | G | A | 0.32723 | 0.01407 | 0.00256 | 4.07E-08 | 30.12 |
| 156 | rs6050446 | G | A | 0.96680 | 0.04308 | 0.00670 | 1.25E-10 | 41.40 |
| 157 | rs60764613 | T | G | 0.14486 | 0.02205 | 0.00339 | 8.24E-11 | 42.20 |
| 158 | rs61649432 | T | C | 0.30959 | 0.01408 | 0.00257 | 4.51E-08 | 29.92 |
| 159 | rs61813324 | T | C | 0.13488 | 0.02586 | 0.00353 | 2.42E-13 | 53.64 |
| 160 | rs61871615 | T | C | 0.09052 | -0.02903 | 0.00433 | 2.07E-11 | 44.91 |
| 161 | rs62037365 | G | C | 0.40213 | 0.03097 | 0.00243 | 2.85E-37 | 162.78 |
| 162 | rs62104483 | A | G | 0.32951 | 0.02346 | 0.00253 | 2.12E-20 | 85.69 |
| 163 | rs62106258 | C | T | 0.04847 | -0.08618 | 0.00554 | 1.71E-54 | 241.74 |
| 164 | rs62246314 | A | G | 0.10128 | 0.02447 | 0.00394 | 5.52E-10 | 38.49 |
| 165 | rs62261725 | G | A | 0.32744 | -0.02140 | 0.00254 | 3.65E-17 | 70.97 |
| 166 | rs62396185 | C | G | 0.25646 | -0.02938 | 0.00273 | 5.18E-27 | 115.85 |
| 167 | rs62477684 | T | C | 0.41964 | -0.02009 | 0.00241 | 8.17E-17 | 69.38 |
| 168 | rs6445198 | T | G | 0.41665 | -0.01483 | 0.00242 | 8.76E-10 | 37.58 |
| 169 | rs649648 | G | A | 0.83645 | -0.01809 | 0.00322 | 1.89E-08 | 31.60 |
| 170 | rs6601527 | A | C | 0.58782 | -0.01817 | 0.00242 | 6.43E-14 | 56.24 |
| 171 | rs6656785 | G | A | 0.39244 | 0.01582 | 0.00244 | 8.48E-11 | 42.15 |
| 172 | rs66679256 | T | C | 0.44614 | 0.01900 | 0.00240 | 2.39E-15 | 62.72 |
| 173 | rs668799 | T | C | 0.27972 | 0.01719 | 0.00265 | 9.05E-11 | 42.02 |
| 174 | rs6699744 | T | A | 0.61402 | 0.02165 | 0.00245 | 1.13E-18 | 77.82 |
| 175 | rs6739755 | G | A | 0.60298 | -0.02058 | 0.00244 | 3.14E-17 | 71.26 |
| 176 | rs67602344 | A | C | 0.19295 | -0.01858 | 0.00302 | 7.99E-10 | 37.76 |
| 177 | rs67807996 | A | G | 0.40199 | 0.01747 | 0.00251 | 3.30E-12 | 48.51 |
| 178 | rs67844506 | G | A | 0.18324 | -0.02422 | 0.00308 | 3.76E-15 | 61.83 |
| 179 | rs6802087 | T | C | 0.49082 | -0.01348 | 0.00238 | 1.57E-08 | 31.96 |
| 180 | rs6861649 | C | T | 0.60749 | 0.01423 | 0.00245 | 6.06E-09 | 33.82 |
| 181 | rs705145 | A | C | 0.34390 | 0.01477 | 0.00250 | 3.68E-09 | 34.79 |
| 182 | rs7094644 | A | G | 0.67418 | 0.01503 | 0.00259 | 6.26E-09 | 33.75 |
| 183 | rs7116641 | G | T | 0.31680 | 0.02452 | 0.00256 | 1.10E-21 | 91.54 |
| 184 | rs7124681 | A | C | 0.40806 | 0.02582 | 0.00242 | 1.24E-26 | 114.12 |
| 185 | rs713586 | C | T | 0.48951 | 0.02988 | 0.00238 | 3.15E-36 | 158.00 |
| 186 | rs7201895 | A | G | 0.35598 | -0.01737 | 0.00250 | 3.77E-12 | 48.24 |
| 187 | rs72892910 | T | G | 0.17044 | 0.03686 | 0.00317 | 3.64E-31 | 134.83 |
| 188 | rs72917533 | C | T | 0.18627 | -0.01756 | 0.00307 | 1.01E-08 | 32.82 |
| 189 | rs73213484 | T | A | 0.13906 | -0.02209 | 0.00344 | 1.38E-10 | 41.19 |
| 190 | rs7442885 | G | C | 0.21010 | -0.02383 | 0.00293 | 4.10E-16 | 66.20 |
| 191 | rs745249 | T | C | 0.28394 | 0.01531 | 0.00265 | 7.65E-09 | 33.36 |
| 192 | rs7498044 | A | G | 0.21866 | -0.01616 | 0.00290 | 2.64E-08 | 30.96 |
| 193 | rs750090 | C | T | 0.35615 | -0.01562 | 0.00251 | 5.01E-10 | 38.67 |
| 194 | rs756717 | A | G | 0.39886 | -0.01566 | 0.00246 | 1.93E-10 | 40.54 |
| 195 | rs76040172 | A | G | 0.05444 | -0.04056 | 0.00526 | 1.26E-14 | 59.44 |
| 196 | rs764729 | C | A | 0.72893 | 0.01537 | 0.00268 | 1.00E-08 | 32.84 |
| 197 | rs7649970 | T | C | 0.12087 | 0.02361 | 0.00364 | 9.23E-11 | 41.98 |
| 198 | rs765876 | G | A | 0.48844 | -0.01351 | 0.00238 | 1.36E-08 | 32.25 |
| 199 | rs76895963 | G | T | 0.01928 | 0.07487 | 0.00924 | 5.20E-16 | 65.73 |
| 200 | rs770082 | A | G | 0.43479 | 0.01835 | 0.00241 | 2.82E-14 | 57.86 |
| 201 | rs7707394 | A | G | 0.35432 | -0.01844 | 0.00248 | 1.14E-13 | 55.12 |
| 202 | rs7723426 | C | T | 0.67502 | 0.01420 | 0.00254 | 2.31E-08 | 31.22 |
| 203 | rs7755574 | T | G | 0.28115 | 0.01556 | 0.00265 | 4.25E-09 | 34.51 |
| 204 | rs7811825 | A | G | 0.13306 | 0.02038 | 0.00351 | 6.19E-09 | 33.77 |
| 205 | rs7845090 | A | G | 0.71112 | -0.02158 | 0.00264 | 3.05E-16 | 66.78 |
| 206 | rs78784145 | A | G | 0.04896 | -0.03405 | 0.00556 | 9.45E-10 | 37.44 |
| 207 | rs79113395 | A | G | 0.26545 | -0.01755 | 0.00271 | 9.04E-11 | 42.02 |
| 208 | rs7930275 | T | C | 0.22125 | 0.01936 | 0.00291 | 2.96E-11 | 44.21 |
| 209 | rs79535757 | T | A | 0.00820 | -0.07420 | 0.01348 | 3.68E-08 | 30.31 |
| 210 | rs7959830 | T | G | 0.41060 | -0.01457 | 0.00242 | 1.76E-09 | 36.22 |
| 211 | rs7987928 | A | G | 0.79963 | -0.01787 | 0.00297 | 1.91E-09 | 36.07 |
| 212 | rs79921461 | C | T | 0.14720 | -0.01868 | 0.00336 | 2.75E-08 | 30.88 |
| 213 | rs8087074 | T | G | 0.26112 | 0.01718 | 0.00272 | 2.76E-10 | 39.84 |
| 214 | rs811715 | A | G | 0.47594 | -0.01466 | 0.00238 | 7.97E-10 | 37.77 |
| 215 | rs815163 | C | T | 0.56160 | -0.01664 | 0.00240 | 3.90E-12 | 48.18 |
| 216 | rs8192675 | C | T | 0.28738 | 0.01759 | 0.00263 | 2.10E-11 | 44.88 |
| 217 | rs846545 | C | T | 0.42032 | -0.01506 | 0.00242 | 4.56E-10 | 38.86 |
| 218 | rs854917 | T | C | 0.73801 | -0.01697 | 0.00272 | 4.47E-10 | 38.90 |
| 219 | rs862320 | T | C | 0.41015 | -0.02032 | 0.00242 | 5.09E-17 | 70.31 |
| 220 | rs869400 | G | T | 0.81583 | 0.02419 | 0.00308 | 3.88E-15 | 61.77 |
| 221 | rs879620 | T | C | 0.61528 | 0.02605 | 0.00246 | 2.74E-26 | 112.55 |
| 222 | rs9289630 | C | G | 0.39076 | 0.01930 | 0.00245 | 3.30E-15 | 62.08 |
| 223 | rs9320823 | C | T | 0.60190 | 0.01868 | 0.00244 | 1.71E-14 | 58.85 |
| 224 | rs935166 | A | G | 0.50694 | -0.01586 | 0.00238 | 2.69E-11 | 44.39 |
| 225 | rs9375702 | T | C | 0.69033 | -0.01632 | 0.00257 | 2.31E-10 | 40.19 |
| 226 | rs9378684 | T | C | 0.20073 | 0.01641 | 0.00300 | 4.38E-08 | 29.97 |
| 227 | rs9496567 | A | G | 0.24238 | 0.01542 | 0.00278 | 2.97E-08 | 30.73 |
| 228 | rs9549099 | C | T | 0.26409 | 0.01574 | 0.00271 | 6.02E-09 | 33.83 |
| 229 | rs9688977 | C | T | 0.14649 | 0.02394 | 0.00338 | 1.34E-12 | 50.28 |
| 230 | rs9835772 | T | A | 0.24276 | 0.01677 | 0.00277 | 1.49E-09 | 36.56 |
| 231 | rs9847186 | A | G | 0.42774 | -0.01484 | 0.00241 | 7.52E-10 | 37.88 |
| 232 | rs9861443 | C | A | 0.71366 | 0.01532 | 0.00265 | 7.39E-09 | 33.43 |

SNPs, single nucleotide polymorphisms; EAF, effect allele frequency; SE, standard error.

**Supplementary Table 3: Characteristics of 235 SNPs used as instrumental variables for the effect of Left leg fat mass on asthma.**

|  | SNP | effect_allele.exposure | other_allele.exposure | EAF.exposure | effect | SE | *P*-value | F-Statistic |
| --- | --- | --- | --- | --- | --- | --- | --- | --- |
| 1 | rs10038105 | G | T | 0.72896 | 0.01485 | 0.00216 | 5.93E-12 | 47.36 |
| 2 | rs10100245 | A | G | 0.56644 | 0.01533 | 0.00194 | 2.39E-15 | 62.72 |
| 3 | rs10128597 | A | G | 0.27402 | -0.01461 | 0.00217 | 1.48E-11 | 45.56 |
| 4 | rs10187101 | T | C | 0.35982 | -0.01205 | 0.00200 | 1.74E-09 | 36.24 |
| 5 | rs10209821 | T | C | 0.34109 | 0.01454 | 0.00202 | 6.68E-13 | 51.64 |
| 6 | rs10215731 | C | T | 0.26496 | 0.01211 | 0.00218 | 2.88E-08 | 30.79 |
| 7 | rs10237306 | T | G | 0.38022 | 0.01243 | 0.00198 | 3.17E-10 | 39.57 |
| 8 | rs10269774 | A | G | 0.32354 | 0.01207 | 0.00205 | 4.11E-09 | 34.57 |
| 9 | rs10423928 | A | T | 0.19349 | -0.02537 | 0.00242 | 1.17E-25 | 109.67 |
| 10 | rs10499014 | G | C | 0.26787 | -0.01465 | 0.00218 | 1.91E-11 | 45.06 |
| 11 | rs10756714 | G | A | 0.44479 | -0.01805 | 0.00193 | 1.07E-20 | 87.04 |
| 12 | rs10756798 | T | C | 0.64503 | -0.01452 | 0.00201 | 4.74E-13 | 52.31 |
| 13 | rs10787738 | T | C | 0.25330 | 0.01670 | 0.00224 | 8.53E-14 | 55.68 |
| 14 | rs10827289 | T | C | 0.58303 | -0.01188 | 0.00195 | 1.15E-09 | 37.06 |
| 15 | rs10835186 | A | G | 0.74176 | 0.01225 | 0.00220 | 2.51E-08 | 31.05 |
| 16 | rs10883553 | A | C | 0.44653 | 0.01094 | 0.00193 | 1.53E-08 | 32.01 |
| 17 | rs10938397 | G | A | 0.43408 | 0.02208 | 0.00194 | 5.34E-30 | 129.50 |
| 18 | rs10954772 | C | T | 0.68571 | -0.01504 | 0.00208 | 4.94E-13 | 52.23 |
| 19 | rs10995427 | A | G | 0.36083 | -0.01225 | 0.00201 | 1.10E-09 | 37.14 |
| 20 | rs1106761 | A | G | 0.38602 | 0.01238 | 0.00200 | 5.47E-10 | 38.50 |
| 21 | rs11100870 | G | T | 0.54958 | -0.01198 | 0.00193 | 5.22E-10 | 38.60 |
| 22 | rs11150745 | G | A | 0.31949 | -0.01632 | 0.00206 | 2.38E-15 | 62.73 |
| 23 | rs111955609 | A | G | 0.08815 | 0.02063 | 0.00340 | 1.28E-09 | 36.85 |
| 24 | rs11223641 | C | T | 0.14462 | -0.01573 | 0.00274 | 9.92E-09 | 32.86 |
| 25 | rs1127100 | C | T | 0.64824 | 0.01447 | 0.00201 | 6.81E-13 | 51.60 |
| 26 | rs1128249 | T | G | 0.39343 | 0.01337 | 0.00196 | 9.81E-12 | 46.37 |
| 27 | rs113230003 | A | G | 0.26019 | -0.01453 | 0.00220 | 4.41E-11 | 43.43 |
| 28 | rs113866544 | C | T | 0.06768 | 0.02862 | 0.00383 | 7.25E-14 | 56.00 |
| 29 | rs115584674 | G | A | 0.05946 | -0.02287 | 0.00405 | 1.66E-08 | 31.85 |
| 30 | rs11611780 | C | G | 0.25432 | 0.01310 | 0.00220 | 2.80E-09 | 35.32 |
| 31 | rs11642015 | T | C | 0.40236 | 0.05126 | 0.00196 | 9.53E-121 | 686.96 |
| 32 | rs11656758 | G | A | 0.33962 | 0.01174 | 0.00203 | 7.22E-09 | 33.48 |
| 33 | rs11668971 | C | T | 0.48114 | -0.01471 | 0.00192 | 1.73E-14 | 58.82 |
| 34 | rs11695700 | T | G | 0.41767 | 0.01113 | 0.00195 | 1.21E-08 | 32.48 |
| 35 | rs11742930 | T | C | 0.56643 | 0.01257 | 0.00194 | 9.49E-11 | 41.93 |
| 36 | rs117632017 | A | G | 0.03914 | 0.02813 | 0.00515 | 4.74E-08 | 29.82 |
| 37 | rs11824092 | C | T | 0.63745 | 0.01248 | 0.00201 | 5.11E-10 | 38.64 |
| 38 | rs11882409 | A | C | 0.28636 | 0.01261 | 0.00216 | 5.12E-09 | 34.15 |
| 39 | rs12001634 | A | T | 0.33642 | -0.01247 | 0.00203 | 8.72E-10 | 37.59 |
| 40 | rs12049202 | T | C | 0.19865 | 0.02164 | 0.00240 | 2.26E-19 | 81.01 |
| 41 | rs1209058 | T | G | 0.73801 | -0.01218 | 0.00219 | 2.81E-08 | 30.83 |
| 42 | rs12140153 | T | G | 0.09676 | -0.02628 | 0.00333 | 2.95E-15 | 62.31 |
| 43 | rs12144626 | C | T | 0.58182 | -0.01170 | 0.00195 | 2.10E-09 | 35.88 |
| 44 | rs12273545 | T | C | 0.05580 | 0.02317 | 0.00418 | 3.02E-08 | 30.70 |
| 45 | rs1229984 | C | T | 0.97755 | 0.03966 | 0.00648 | 9.53E-10 | 37.42 |
| 46 | rs12330631 | T | C | 0.36711 | -0.01216 | 0.00200 | 1.08E-09 | 37.17 |
| 47 | rs12339822 | G | A | 0.54320 | 0.01269 | 0.00193 | 5.36E-11 | 43.04 |
| 48 | rs12439200 | A | G | 0.23852 | -0.01661 | 0.00226 | 2.00E-13 | 54.00 |
| 49 | rs12477385 | T | G | 0.22730 | -0.01541 | 0.00230 | 2.00E-11 | 44.97 |
| 50 | rs12479357 | G | A | 0.63108 | 0.01506 | 0.00200 | 4.53E-14 | 56.93 |
| 51 | rs12578952 | G | A | 0.25170 | -0.01511 | 0.00221 | 8.44E-12 | 46.66 |
| 52 | rs12583872 | C | A | 0.30973 | -0.01190 | 0.00208 | 9.91E-09 | 32.86 |
| 53 | rs12619178 | T | C | 0.40292 | -0.01483 | 0.00196 | 3.49E-14 | 57.44 |
| 54 | rs12679106 | T | G | 0.71134 | -0.01969 | 0.00213 | 2.35E-20 | 85.48 |
| 55 | rs12680342 | G | T | 0.22910 | -0.01429 | 0.00228 | 3.64E-10 | 39.30 |
| 56 | rs12714415 | C | T | 0.16687 | -0.03808 | 0.00264 | 4.67E-47 | 207.63 |
| 57 | rs12756213 | A | G | 0.61810 | 0.01157 | 0.00198 | 4.79E-09 | 34.27 |
| 58 | rs1286065 | T | C | 0.70270 | 0.01173 | 0.00210 | 2.38E-08 | 31.16 |
| 59 | rs12877270 | A | G | 0.43845 | 0.01168 | 0.00195 | 2.07E-09 | 35.91 |
| 60 | rs12890931 | G | T | 0.36346 | 0.01168 | 0.00201 | 5.98E-09 | 33.84 |
| 61 | rs12891263 | A | G | 0.14317 | -0.01541 | 0.00274 | 1.93E-08 | 31.56 |
| 62 | rs1296328 | C | A | 0.55991 | -0.01441 | 0.00194 | 1.18E-13 | 55.04 |
| 63 | rs13062093 | G | T | 0.36644 | 0.01455 | 0.00199 | 2.66E-13 | 53.45 |
| 64 | rs13135092 | G | A | 0.08323 | 0.03130 | 0.00350 | 3.55E-19 | 80.11 |
| 65 | rs13174863 | G | A | 0.14821 | 0.01936 | 0.00272 | 1.05E-12 | 50.75 |
| 66 | rs13333747 | C | T | 0.18196 | -0.01847 | 0.00250 | 1.46E-13 | 54.63 |
| 67 | rs1335055 | A | G | 0.65578 | -0.01263 | 0.00204 | 5.86E-10 | 38.37 |
| 68 | rs13427822 | G | A | 0.27205 | -0.01472 | 0.00218 | 1.49E-11 | 45.54 |
| 69 | rs1412239 | G | C | 0.32454 | 0.01769 | 0.00205 | 5.54E-18 | 74.69 |
| 70 | rs1441264 | A | G | 0.59187 | 0.01475 | 0.00199 | 1.40E-13 | 54.72 |
| 71 | rs1446585 | G | A | 0.22801 | -0.01462 | 0.00230 | 1.99E-10 | 40.48 |
| 72 | rs1458156 | T | C | 0.48826 | 0.01254 | 0.00192 | 6.85E-11 | 42.56 |
| 73 | rs1470749 | T | G | 0.51060 | -0.01098 | 0.00192 | 1.12E-08 | 32.62 |
| 74 | rs147730268 | T | G | 0.09053 | -0.03275 | 0.00346 | 2.80E-21 | 89.69 |
| 75 | rs1491905 | C | T | 0.50632 | -0.01190 | 0.00192 | 5.74E-10 | 38.41 |
| 76 | rs1503527 | T | C | 0.47972 | 0.01327 | 0.00192 | 4.88E-12 | 47.74 |
| 77 | rs1524277 | T | C | 0.52347 | -0.01062 | 0.00192 | 3.28E-08 | 30.53 |
| 78 | rs1538742 | A | C | 0.56959 | -0.01318 | 0.00195 | 1.23E-11 | 45.93 |
| 79 | rs17024393 | C | T | 0.02580 | 0.04813 | 0.00607 | 2.32E-15 | 62.78 |
| 80 | rs17149254 | C | T | 0.80856 | -0.01685 | 0.00249 | 1.29E-11 | 45.83 |
| 81 | rs17522122 | T | G | 0.47032 | 0.01400 | 0.00193 | 4.03E-13 | 52.63 |
| 82 | rs1782508 | G | C | 0.65633 | -0.01191 | 0.00202 | 3.56E-09 | 34.85 |
| 83 | rs1788808 | G | A | 0.49734 | -0.01753 | 0.00192 | 7.61E-20 | 83.16 |
| 84 | rs1928496 | T | C | 0.74239 | 0.01450 | 0.00219 | 3.80E-11 | 43.71 |
| 85 | rs1945391 | T | A | 0.38782 | 0.01076 | 0.00197 | 4.56E-08 | 29.90 |
| 86 | rs2016469 | A | G | 0.37370 | 0.01217 | 0.00200 | 1.14E-09 | 37.07 |
| 87 | rs208477 | T | C | 0.18643 | 0.01424 | 0.00246 | 7.52E-09 | 33.40 |
| 88 | rs2102278 | G | A | 0.32208 | 0.01190 | 0.00206 | 7.38E-09 | 33.43 |
| 89 | rs2111455 | T | C | 0.67820 | 0.01125 | 0.00206 | 4.51E-08 | 29.92 |
| 90 | rs2155869 | C | T | 0.81916 | -0.01434 | 0.00249 | 8.05E-09 | 33.27 |
| 91 | rs2172131 | C | T | 0.58064 | -0.01405 | 0.00195 | 5.15E-13 | 52.15 |
| 92 | rs2181375 | G | A | 0.59863 | 0.01463 | 0.00196 | 7.55E-14 | 55.92 |
| 93 | rs2292238 | C | A | 0.40867 | -0.01226 | 0.00196 | 3.69E-10 | 39.27 |
| 94 | rs2307111 | C | T | 0.39318 | -0.02296 | 0.00197 | 1.81E-31 | 136.22 |
| 95 | rs2318543 | G | A | 0.78250 | -0.01360 | 0.00233 | 5.76E-09 | 33.92 |
| 96 | rs2370982 | T | C | 0.21534 | 0.02255 | 0.00235 | 9.58E-22 | 91.81 |
| 97 | rs2374947 | G | A | 0.76098 | 0.01263 | 0.00226 | 2.31E-08 | 31.22 |
| 98 | rs2398861 | G | A | 0.25660 | 0.01566 | 0.00221 | 1.27E-12 | 50.37 |
| 99 | rs2409742 | T | C | 0.48505 | -0.01278 | 0.00192 | 3.08E-11 | 44.13 |
| 100 | rs241459 | C | A | 0.68028 | -0.01743 | 0.00205 | 2.13E-17 | 72.03 |
| 101 | rs2418507 | T | C | 0.57421 | 0.01248 | 0.00195 | 1.44E-10 | 41.11 |
| 102 | rs2439823 | G | A | 0.54771 | 0.01605 | 0.00193 | 1.04E-16 | 68.89 |
| 103 | rs2455386 | G | A | 0.67961 | -0.01134 | 0.00207 | 4.13E-08 | 30.09 |
| 104 | rs252749 | A | G | 0.24657 | -0.01417 | 0.00222 | 1.82E-10 | 40.66 |
| 105 | rs2606228 | C | A | 0.64277 | -0.01145 | 0.00203 | 1.59E-08 | 31.94 |
| 106 | rs2678204 | G | T | 0.34181 | 0.02021 | 0.00203 | 1.98E-23 | 99.50 |
| 107 | rs2737251 | T | C | 0.33800 | -0.01596 | 0.00203 | 3.72E-15 | 61.85 |
| 108 | rs28366156 | C | T | 0.13136 | -0.02302 | 0.00284 | 5.46E-16 | 65.63 |
| 109 | rs28457808 | G | C | 0.16319 | -0.01661 | 0.00260 | 1.75E-10 | 40.73 |
| 110 | rs2861685 | C | T | 0.41137 | -0.01383 | 0.00195 | 1.27E-12 | 50.39 |
| 111 | rs286818 | A | T | 0.17025 | -0.01909 | 0.00256 | 9.10E-14 | 55.56 |
| 112 | rs28726372 | C | T | 0.30781 | 0.01186 | 0.00208 | 1.20E-08 | 32.49 |
| 113 | rs290483 | T | G | 0.59921 | -0.01080 | 0.00196 | 3.79E-08 | 30.26 |
| 114 | rs2917705 | A | G | 0.13939 | -0.01586 | 0.00278 | 1.10E-08 | 32.65 |
| 115 | rs2954021 | G | A | 0.50596 | 0.01170 | 0.00192 | 1.13E-09 | 37.09 |
| 116 | rs301806 | T | C | 0.58423 | 0.01167 | 0.00195 | 2.06E-09 | 35.92 |
| 117 | rs34045288 | T | C | 0.33590 | 0.01968 | 0.00203 | 3.72E-22 | 93.69 |
| 118 | rs34769775 | T | C | 0.29786 | -0.01533 | 0.00210 | 2.57E-13 | 53.51 |
| 119 | rs34966008 | T | C | 0.40815 | -0.01553 | 0.00195 | 1.79E-15 | 63.29 |
| 120 | rs35025195 | A | G | 0.16613 | -0.01991 | 0.00258 | 1.21E-14 | 59.52 |
| 121 | rs35154326 | G | A | 0.27246 | -0.01329 | 0.00217 | 9.36E-10 | 37.45 |
| 122 | rs35626515 | A | C | 0.40797 | 0.02169 | 0.00195 | 1.29E-28 | 123.18 |
| 123 | rs35851183 | G | A | 0.35778 | 0.01254 | 0.00200 | 3.92E-10 | 39.16 |
| 124 | rs35972789 | A | C | 0.03777 | -0.03015 | 0.00503 | 1.99E-09 | 35.98 |
| 125 | rs3749472 | G | A | 0.46158 | 0.01052 | 0.00193 | 4.80E-08 | 29.80 |
| 126 | rs3784699 | C | T | 0.60302 | 0.01928 | 0.00196 | 9.64E-23 | 96.36 |
| 127 | rs3803286 | G | A | 0.66638 | -0.01728 | 0.00203 | 1.93E-17 | 72.22 |
| 128 | rs3897102 | T | C | 0.41225 | 0.01237 | 0.00197 | 3.07E-10 | 39.63 |
| 129 | rs4073582 | A | G | 0.35979 | -0.01603 | 0.00200 | 1.15E-15 | 64.16 |
| 130 | rs444102 | C | G | 0.17110 | 0.01586 | 0.00255 | 4.97E-10 | 38.69 |
| 131 | rs4467770 | A | G | 0.73067 | 0.01438 | 0.00217 | 3.49E-11 | 43.88 |
| 132 | rs4482463 | A | C | 0.92418 | -0.02480 | 0.00363 | 8.78E-12 | 46.59 |
| 133 | rs4502882 | T | C | 0.65689 | -0.01346 | 0.00202 | 2.74E-11 | 44.36 |
| 134 | rs4503172 | T | C | 0.60782 | -0.01212 | 0.00197 | 7.27E-10 | 37.95 |
| 135 | rs4657796 | C | T | 0.37656 | -0.01185 | 0.00199 | 2.36E-09 | 35.66 |
| 136 | rs4673617 | T | C | 0.27164 | 0.01376 | 0.00216 | 2.04E-10 | 40.43 |
| 137 | rs4678440 | T | A | 0.14956 | -0.01478 | 0.00269 | 3.85E-08 | 30.23 |
| 138 | rs4682711 | C | T | 0.40868 | -0.01180 | 0.00195 | 1.43E-09 | 36.63 |
| 139 | rs4718964 | T | G | 0.41219 | 0.01416 | 0.00195 | 4.45E-13 | 52.44 |
| 140 | rs4911382 | T | C | 0.58424 | 0.01246 | 0.00195 | 1.73E-10 | 40.75 |
| 141 | rs4958702 | C | T | 0.57368 | -0.01275 | 0.00194 | 4.88E-11 | 43.23 |
| 142 | rs4981693 | A | G | 0.77273 | 0.01726 | 0.00229 | 5.33E-14 | 56.61 |
| 143 | rs525101 | C | T | 0.37196 | 0.01377 | 0.00199 | 4.47E-12 | 47.91 |
| 144 | rs541577 | G | A | 0.62126 | -0.01382 | 0.00199 | 3.56E-12 | 48.35 |
| 145 | rs543874 | G | A | 0.20781 | 0.03298 | 0.00236 | 3.04E-44 | 194.73 |
| 146 | rs55707359 | G | T | 0.01503 | 0.04351 | 0.00795 | 4.40E-08 | 29.97 |
| 147 | rs55714539 | C | A | 0.34269 | 0.01773 | 0.00204 | 2.95E-18 | 75.93 |
| 148 | rs55726687 | A | G | 0.21114 | 0.01822 | 0.00235 | 9.21E-15 | 60.06 |
| 149 | rs55745410 | G | A | 0.35429 | -0.01147 | 0.00202 | 1.32E-08 | 32.30 |
| 150 | rs56398417 | T | C | 0.31293 | -0.01345 | 0.00207 | 8.29E-11 | 42.19 |
| 151 | rs56803094 | G | A | 0.22711 | -0.01645 | 0.00230 | 7.81E-13 | 51.33 |
| 152 | rs57636386 | C | T | 0.08342 | -0.03151 | 0.00348 | 1.28E-19 | 82.12 |
| 153 | rs57947134 | G | C | 0.15832 | 0.01446 | 0.00264 | 4.09E-08 | 30.11 |
| 154 | rs58862095 | T | C | 0.41976 | -0.01559 | 0.00195 | 1.27E-15 | 63.97 |
| 155 | rs58989684 | T | A | 0.32971 | 0.01712 | 0.00204 | 5.56E-17 | 70.14 |
| 156 | rs59066241 | G | T | 0.11827 | 0.01801 | 0.00299 | 1.64E-09 | 36.36 |
| 157 | rs59104534 | T | C | 0.30111 | 0.01262 | 0.00210 | 1.91E-09 | 36.06 |
| 158 | rs59227842 | G | A | 0.31243 | 0.01897 | 0.00209 | 1.16E-19 | 82.32 |
| 159 | rs6050446 | G | A | 0.96680 | 0.03640 | 0.00540 | 1.58E-11 | 45.44 |
| 160 | rs60764613 | T | G | 0.14486 | 0.01603 | 0.00274 | 4.71E-09 | 34.31 |
| 161 | rs61765651 | T | C | 0.19241 | -0.02649 | 0.00243 | 1.35E-27 | 118.52 |
| 162 | rs61813324 | T | C | 0.13488 | 0.01838 | 0.00285 | 1.09E-10 | 41.66 |
| 163 | rs61826865 | A | T | 0.10894 | 0.01778 | 0.00309 | 8.32E-09 | 33.20 |
| 164 | rs61888762 | G | C | 0.32201 | 0.02491 | 0.00205 | 7.02E-34 | 147.25 |
| 165 | rs61903695 | G | A | 0.25600 | 0.01200 | 0.00220 | 4.80E-08 | 29.80 |
| 166 | rs62246314 | A | G | 0.10128 | 0.01822 | 0.00318 | 1.02E-08 | 32.80 |
| 167 | rs62261725 | G | A | 0.32744 | -0.01912 | 0.00205 | 1.03E-20 | 87.12 |
| 168 | rs62346281 | T | C | 0.51584 | 0.01051 | 0.00193 | 4.82E-08 | 29.79 |
| 169 | rs62396185 | C | G | 0.25646 | -0.02623 | 0.00220 | 9.67E-33 | 142.04 |
| 170 | rs62407569 | G | A | 0.27621 | 0.01479 | 0.00215 | 5.98E-12 | 47.34 |
| 171 | rs62444907 | T | C | 0.15303 | -0.01686 | 0.00267 | 2.87E-10 | 39.77 |
| 172 | rs6536575 | C | T | 0.51850 | 0.01071 | 0.00192 | 2.49E-08 | 31.07 |
| 173 | rs6561937 | A | T | 0.75176 | -0.01531 | 0.00223 | 6.43E-12 | 47.20 |
| 174 | rs6567160 | C | T | 0.23406 | 0.03778 | 0.00226 | 1.91E-62 | 278.26 |
| 175 | rs66679256 | T | C | 0.44614 | 0.01410 | 0.00193 | 3.21E-13 | 53.08 |
| 176 | rs6739755 | G | A | 0.60298 | -0.01601 | 0.00197 | 3.91E-16 | 66.29 |
| 177 | rs67602344 | A | C | 0.19295 | -0.01752 | 0.00244 | 6.76E-13 | 51.62 |
| 178 | rs67844506 | G | A | 0.18324 | -0.02089 | 0.00248 | 4.06E-17 | 70.75 |
| 179 | rs6861649 | C | T | 0.60749 | 0.01372 | 0.00197 | 3.50E-12 | 48.39 |
| 180 | rs6875585 | C | A | 0.67000 | 0.01217 | 0.00205 | 2.74E-09 | 35.36 |
| 181 | rs6898357 | G | A | 0.68048 | -0.01184 | 0.00206 | 8.52E-09 | 33.15 |
| 182 | rs7027304 | T | C | 0.65245 | 0.01124 | 0.00202 | 2.81E-08 | 30.83 |
| 183 | rs704061 | C | T | 0.45270 | 0.01413 | 0.00193 | 2.39E-13 | 53.66 |
| 184 | rs7124681 | A | C | 0.40806 | 0.01877 | 0.00195 | 6.26E-22 | 92.66 |
| 185 | rs7132908 | A | G | 0.38392 | 0.02236 | 0.00198 | 1.08E-29 | 128.10 |
| 186 | rs713586 | C | T | 0.48951 | 0.02254 | 0.00192 | 6.79E-32 | 138.17 |
| 187 | rs7193783 | C | A | 0.64917 | -0.01312 | 0.00202 | 8.70E-11 | 42.10 |
| 188 | rs7206608 | G | C | 0.32260 | 0.01175 | 0.00205 | 1.07E-08 | 32.71 |
| 189 | rs7259070 | C | T | 0.59901 | 0.01594 | 0.00198 | 7.23E-16 | 65.08 |
| 190 | rs72740701 | G | A | 0.28206 | -0.01188 | 0.00214 | 2.78E-08 | 30.86 |
| 191 | rs72820274 | A | G | 0.41876 | 0.01195 | 0.00195 | 8.75E-10 | 37.59 |
| 192 | rs72892910 | T | G | 0.17044 | 0.02971 | 0.00256 | 3.96E-31 | 134.67 |
| 193 | rs72917544 | A | G | 0.18562 | -0.01412 | 0.00248 | 1.26E-08 | 32.40 |
| 194 | rs73035223 | G | A | 0.14854 | 0.01630 | 0.00271 | 1.71E-09 | 36.29 |
| 195 | rs73213484 | T | A | 0.13906 | -0.01792 | 0.00278 | 1.07E-10 | 41.69 |
| 196 | rs7442885 | G | C | 0.21010 | -0.02167 | 0.00236 | 4.57E-20 | 84.17 |
| 197 | rs7460093 | A | G | 0.53551 | 0.01078 | 0.00194 | 2.82E-08 | 30.83 |
| 198 | rs7498044 | A | G | 0.21866 | -0.01312 | 0.00234 | 2.13E-08 | 31.37 |
| 199 | rs750090 | C | T | 0.35615 | -0.01195 | 0.00203 | 3.60E-09 | 34.83 |
| 200 | rs75557510 | G | A | 0.06150 | -0.02890 | 0.00408 | 1.37E-12 | 50.23 |
| 201 | rs75641275 | C | A | 0.14395 | 0.01637 | 0.00273 | 2.13E-09 | 35.85 |
| 202 | rs76040172 | A | G | 0.05444 | -0.03210 | 0.00424 | 3.90E-14 | 57.22 |
| 203 | rs7613261 | T | A | 0.20456 | 0.01950 | 0.00238 | 2.88E-16 | 66.89 |
| 204 | rs7649970 | T | C | 0.12087 | 0.02248 | 0.00294 | 1.97E-14 | 58.56 |
| 205 | rs76895963 | G | T | 0.01928 | 0.05756 | 0.00745 | 1.09E-14 | 59.73 |
| 206 | rs7707394 | A | G | 0.35432 | -0.01604 | 0.00200 | 1.18E-15 | 64.12 |
| 207 | rs77165542 | T | C | 0.03575 | -0.06829 | 0.00525 | 1.19E-38 | 169.10 |
| 208 | rs7774 | A | C | 0.30914 | 0.01233 | 0.00209 | 3.61E-09 | 34.82 |
| 209 | rs778094 | A | G | 0.57757 | -0.01130 | 0.00195 | 6.54E-09 | 33.67 |
| 210 | rs78565420 | T | C | 0.05419 | 0.02569 | 0.00436 | 3.80E-09 | 34.72 |
| 211 | rs7893571 | T | G | 0.66342 | 0.01265 | 0.00204 | 5.09E-10 | 38.64 |
| 212 | rs79113395 | A | G | 0.26545 | -0.01448 | 0.00218 | 3.31E-11 | 43.99 |
| 213 | rs7925100 | A | G | 0.39634 | 0.01209 | 0.00196 | 7.16E-10 | 37.98 |
| 214 | rs7933085 | G | A | 0.50920 | 0.01194 | 0.00193 | 6.08E-10 | 38.30 |
| 215 | rs79704991 | T | G | 0.12585 | 0.01788 | 0.00290 | 6.91E-10 | 38.05 |
| 216 | rs7987928 | A | G | 0.79963 | -0.01488 | 0.00240 | 5.57E-10 | 38.47 |
| 217 | rs799449 | T | C | 0.55848 | 0.01472 | 0.00194 | 3.25E-14 | 57.58 |
| 218 | rs8078135 | T | C | 0.48847 | -0.01200 | 0.00193 | 4.78E-10 | 38.77 |
| 219 | rs8087074 | T | G | 0.26112 | 0.01252 | 0.00220 | 1.19E-08 | 32.50 |
| 220 | rs815163 | C | T | 0.56160 | -0.01338 | 0.00193 | 4.46E-12 | 47.92 |
| 221 | rs8192675 | C | T | 0.28738 | 0.01348 | 0.00212 | 1.92E-10 | 40.54 |
| 222 | rs869400 | G | T | 0.81583 | 0.01833 | 0.00248 | 1.52E-13 | 54.55 |
| 223 | rs879620 | T | C | 0.61528 | 0.02071 | 0.00198 | 1.34E-25 | 109.40 |
| 224 | rs882378 | C | A | 0.30937 | 0.01180 | 0.00208 | 1.46E-08 | 32.11 |
| 225 | rs9289630 | C | G | 0.39076 | 0.01579 | 0.00198 | 1.32E-15 | 63.88 |
| 226 | rs9320823 | C | T | 0.60190 | 0.01702 | 0.00196 | 4.42E-18 | 75.13 |
| 227 | rs935166 | A | G | 0.50694 | -0.01189 | 0.00192 | 5.89E-10 | 38.36 |
| 228 | rs9375702 | T | C | 0.69033 | -0.01329 | 0.00208 | 1.53E-10 | 40.99 |
| 229 | rs9378684 | T | C | 0.20073 | 0.01354 | 0.00242 | 2.11E-08 | 31.39 |
| 230 | rs9569933 | T | C | 0.18945 | -0.01485 | 0.00246 | 1.48E-09 | 36.56 |
| 231 | rs9584855 | G | T | 0.29010 | -0.01169 | 0.00211 | 3.16E-08 | 30.61 |
| 232 | rs9610311 | C | T | 0.30652 | 0.01184 | 0.00214 | 3.07E-08 | 30.67 |
| 233 | rs9835772 | T | A | 0.24276 | 0.01390 | 0.00224 | 5.16E-10 | 38.62 |
| 234 | rs9847186 | A | G | 0.42774 | -0.01119 | 0.00194 | 8.55E-09 | 33.15 |
| 235 | rs9863890 | G | A | 0.40860 | -0.01172 | 0.00195 | 1.86E-09 | 36.11 |

SNPs, single nucleotide polymorphisms; EAF, effect allele frequency; SE, standard error.

**Supplementary Table 4: Characteristics of 232 SNPs used as instrumental variables for the effect of Right arm fat mass on asthma.**

|  | SNP | effect_allele.exposure | other_allele.exposure | EAF.exposure | effect | SE | *P*-value | F-Statistic |
| --- | --- | --- | --- | --- | --- | --- | --- | --- |
| 1 | rs10100245 | A | G | 0.56644 | 0.02028 | 0.00240 | 2.97E-17 | 71.37 |
| 2 | rs10187101 | T | C | 0.35982 | -0.01515 | 0.00248 | 1.02E-09 | 37.29 |
| 3 | rs10236214 | T | C | 0.64140 | 0.01650 | 0.00250 | 3.87E-11 | 43.68 |
| 4 | rs10237306 | T | G | 0.38022 | 0.01555 | 0.00245 | 2.23E-10 | 40.26 |
| 5 | rs10237317 | G | A | 0.41697 | 0.01668 | 0.00242 | 5.96E-12 | 47.35 |
| 6 | rs10259620 | G | A | 0.78717 | -0.01768 | 0.00293 | 1.53E-09 | 36.50 |
| 7 | rs10269774 | A | G | 0.32354 | 0.01654 | 0.00255 | 8.16E-11 | 42.22 |
| 8 | rs10404726 | T | C | 0.46678 | -0.01831 | 0.00238 | 1.60E-14 | 58.98 |
| 9 | rs10423928 | A | T | 0.19349 | -0.03012 | 0.00300 | 1.14E-23 | 100.60 |
| 10 | rs10492229 | T | C | 0.23830 | 0.01561 | 0.00279 | 2.32E-08 | 31.21 |
| 11 | rs10499014 | G | C | 0.26787 | -0.01513 | 0.00271 | 2.26E-08 | 31.26 |
| 12 | rs10745787 | A | G | 0.66813 | -0.01415 | 0.00252 | 2.06E-08 | 31.44 |
| 13 | rs10756798 | T | C | 0.64503 | -0.01787 | 0.00249 | 6.94E-13 | 51.57 |
| 14 | rs10760724 | C | T | 0.47394 | 0.01569 | 0.00238 | 4.44E-11 | 43.41 |
| 15 | rs10787738 | T | C | 0.25330 | 0.01902 | 0.00277 | 7.09E-12 | 47.00 |
| 16 | rs10873302 | T | C | 0.29343 | 0.01677 | 0.00263 | 1.97E-10 | 40.49 |
| 17 | rs10938398 | A | G | 0.43334 | 0.02789 | 0.00241 | 5.09E-31 | 134.17 |
| 18 | rs10947793 | G | A | 0.37360 | -0.01441 | 0.00247 | 5.62E-09 | 33.96 |
| 19 | rs10954772 | C | T | 0.68571 | -0.01673 | 0.00258 | 8.89E-11 | 42.05 |
| 20 | rs10995427 | A | G | 0.36083 | -0.01362 | 0.00249 | 4.66E-08 | 29.85 |
| 21 | rs11030119 | A | G | 0.30881 | 0.03222 | 0.00258 | 7.05E-36 | 156.40 |
| 22 | rs11042030 | C | T | 0.27401 | -0.01905 | 0.00267 | 9.85E-13 | 50.88 |
| 23 | rs11150745 | G | A | 0.31949 | -0.01984 | 0.00256 | 8.20E-15 | 60.29 |
| 24 | rs11152135 | C | G | 0.76339 | 0.01647 | 0.00281 | 4.67E-09 | 34.32 |
| 25 | rs11165643 | T | C | 0.59231 | 0.02046 | 0.00242 | 2.95E-17 | 71.39 |
| 26 | rs11205277 | G | A | 0.43972 | 0.01688 | 0.00239 | 1.82E-12 | 49.67 |
| 27 | rs11205617 | A | G | 0.31713 | 0.02048 | 0.00255 | 9.84E-16 | 64.47 |
| 28 | rs11245480 | G | C | 0.32474 | 0.01405 | 0.00254 | 3.20E-08 | 30.58 |
| 29 | rs112566467 | T | C | 0.21334 | 0.02096 | 0.00293 | 7.91E-13 | 51.31 |
| 30 | rs1127100 | C | T | 0.64824 | 0.01608 | 0.00250 | 1.22E-10 | 41.43 |
| 31 | rs113182412 | A | G | 0.16592 | -0.01917 | 0.00325 | 3.85E-09 | 34.70 |
| 32 | rs113230003 | A | G | 0.26019 | -0.01968 | 0.00273 | 5.97E-13 | 51.86 |
| 33 | rs113866544 | C | T | 0.06768 | 0.03538 | 0.00474 | 8.65E-14 | 55.66 |
| 34 | rs11474838 | G | T | 0.43273 | 0.01525 | 0.00245 | 4.80E-10 | 38.76 |
| 35 | rs11515071 | T | C | 0.36514 | -0.02303 | 0.00248 | 1.71E-20 | 86.11 |
| 36 | rs11538 | G | A | 0.17213 | 0.01809 | 0.00315 | 9.34E-09 | 32.98 |
| 37 | rs11609659 | C | T | 0.24589 | -0.01759 | 0.00278 | 2.55E-10 | 40.00 |
| 38 | rs11642015 | T | C | 0.40236 | 0.06438 | 0.00242 | 9.53E-121 | 705.18 |
| 39 | rs117632017 | A | G | 0.03914 | 0.03815 | 0.00639 | 2.32E-09 | 35.68 |
| 40 | rs11766945 | A | G | 0.20101 | -0.01962 | 0.00299 | 5.18E-11 | 43.11 |
| 41 | rs11782341 | G | A | 0.18851 | 0.01900 | 0.00306 | 5.64E-10 | 38.45 |
| 42 | rs11786089 | G | A | 0.45685 | 0.01370 | 0.00239 | 1.04E-08 | 32.77 |
| 43 | rs1182143 | C | T | 0.29630 | -0.01915 | 0.00261 | 2.00E-13 | 54.01 |
| 44 | rs11882409 | A | C | 0.28636 | 0.01921 | 0.00268 | 6.97E-13 | 51.56 |
| 45 | rs11920781 | C | T | 0.36517 | 0.01673 | 0.00247 | 1.38E-11 | 45.71 |
| 46 | rs12001634 | A | T | 0.33642 | -0.01510 | 0.00252 | 2.08E-09 | 35.90 |
| 47 | rs12072739 | G | A | 0.22460 | 0.01725 | 0.00285 | 1.50E-09 | 36.54 |
| 48 | rs12128526 | A | G | 0.45998 | 0.01347 | 0.00239 | 1.76E-08 | 31.75 |
| 49 | rs12140153 | T | G | 0.09676 | -0.03415 | 0.00413 | 1.31E-16 | 68.45 |
| 50 | rs12254441 | T | C | 0.37284 | -0.01489 | 0.00255 | 5.05E-09 | 34.17 |
| 51 | rs12316080 | T | C | 0.33432 | 0.01576 | 0.00252 | 3.93E-10 | 39.15 |
| 52 | rs12339822 | G | A | 0.54320 | 0.01603 | 0.00240 | 2.34E-11 | 44.66 |
| 53 | rs12367809 | T | C | 0.36828 | 0.02877 | 0.00248 | 3.47E-31 | 134.93 |
| 54 | rs12442480 | G | A | 0.28408 | -0.01510 | 0.00264 | 1.08E-08 | 32.69 |
| 55 | rs12578952 | G | A | 0.25170 | -0.01789 | 0.00274 | 6.74E-11 | 42.60 |
| 56 | rs12692738 | C | T | 0.22531 | 0.01996 | 0.00287 | 3.43E-12 | 48.43 |
| 57 | rs12756213 | A | G | 0.61810 | 0.01354 | 0.00245 | 3.23E-08 | 30.57 |
| 58 | rs1286065 | T | C | 0.70270 | 0.01501 | 0.00261 | 8.31E-09 | 33.20 |
| 59 | rs12877270 | A | G | 0.43845 | 0.01487 | 0.00242 | 7.67E-10 | 37.84 |
| 60 | rs12885458 | G | T | 0.50998 | -0.01420 | 0.00238 | 2.51E-09 | 35.54 |
| 61 | rs12887636 | G | T | 0.34740 | -0.01565 | 0.00252 | 4.99E-10 | 38.68 |
| 62 | rs1293395 | T | G | 0.07636 | -0.02476 | 0.00449 | 3.54E-08 | 30.39 |
| 63 | rs12992672 | A | G | 0.82864 | 0.04748 | 0.00315 | 3.07E-51 | 226.82 |
| 64 | rs13062093 | G | T | 0.36644 | 0.01603 | 0.00247 | 8.21E-11 | 42.21 |
| 65 | rs13076052 | G | C | 0.27704 | 0.01469 | 0.00267 | 3.95E-08 | 30.18 |
| 66 | rs13135092 | G | A | 0.08323 | 0.04293 | 0.00433 | 3.98E-23 | 98.12 |
| 67 | rs13174863 | G | A | 0.14821 | 0.02677 | 0.00337 | 1.95E-15 | 63.12 |
| 68 | rs1335055 | A | G | 0.65578 | -0.01464 | 0.00253 | 7.09E-09 | 33.51 |
| 69 | rs13427822 | G | A | 0.27205 | -0.01631 | 0.00270 | 1.61E-09 | 36.40 |
| 70 | rs1406778 | C | T | 0.19754 | 0.02296 | 0.00299 | 1.48E-14 | 59.14 |
| 71 | rs1441264 | A | G | 0.59187 | 0.01945 | 0.00247 | 3.72E-15 | 61.85 |
| 72 | rs1446585 | G | A | 0.22801 | -0.01721 | 0.00285 | 1.54E-09 | 36.48 |
| 73 | rs1449630 | G | A | 0.57036 | 0.01338 | 0.00242 | 3.06E-08 | 30.67 |
| 74 | rs1470749 | T | G | 0.51060 | -0.01390 | 0.00238 | 5.40E-09 | 34.04 |
| 75 | rs1471338 | G | A | 0.29080 | 0.01463 | 0.00262 | 2.50E-08 | 31.06 |
| 76 | rs147730268 | T | G | 0.09053 | -0.04290 | 0.00429 | 1.43E-23 | 100.14 |
| 77 | rs1503526 | C | T | 0.47918 | 0.01646 | 0.00238 | 4.76E-12 | 47.79 |
| 78 | rs1516725 | C | T | 0.86378 | 0.02797 | 0.00348 | 8.68E-16 | 64.72 |
| 79 | rs1524445 | T | C | 0.42041 | -0.01824 | 0.00243 | 5.96E-14 | 56.39 |
| 80 | rs1568488 | C | G | 0.59563 | 0.01546 | 0.00245 | 2.59E-10 | 39.97 |
| 81 | rs17024393 | C | T | 0.02580 | 0.06926 | 0.00753 | 3.69E-20 | 84.59 |
| 82 | rs17149254 | C | T | 0.80856 | -0.02127 | 0.00309 | 5.52E-12 | 47.49 |
| 83 | rs1724551 | A | G | 0.51213 | -0.01584 | 0.00240 | 4.14E-11 | 43.55 |
| 84 | rs17627049 | A | C | 0.27425 | 0.01644 | 0.00267 | 7.07E-10 | 38.00 |
| 85 | rs17716502 | T | C | 0.20724 | -0.02541 | 0.00296 | 8.58E-18 | 73.82 |
| 86 | rs17770336 | T | C | 0.32382 | 0.02365 | 0.00254 | 1.22E-20 | 86.78 |
| 87 | rs1782508 | G | C | 0.65633 | -0.01551 | 0.00250 | 5.61E-10 | 38.46 |
| 88 | rs1840126 | C | A | 0.56206 | -0.01341 | 0.00240 | 2.25E-08 | 31.27 |
| 89 | rs1901241 | G | A | 0.16014 | 0.02007 | 0.00326 | 7.38E-10 | 37.92 |
| 90 | rs1927626 | T | C | 0.93631 | 0.02691 | 0.00488 | 3.54E-08 | 30.39 |
| 91 | rs1928496 | T | C | 0.74239 | 0.01671 | 0.00272 | 7.88E-10 | 37.79 |
| 92 | rs1979440 | C | T | 0.40199 | -0.01410 | 0.00243 | 6.42E-09 | 33.70 |
| 93 | rs2016469 | A | G | 0.37370 | 0.01535 | 0.00248 | 6.01E-10 | 38.32 |
| 94 | rs2102278 | G | A | 0.32208 | 0.01623 | 0.00255 | 2.02E-10 | 40.45 |
| 95 | rs2239647 | C | A | 0.55179 | -0.01895 | 0.00239 | 2.43E-15 | 62.69 |
| 96 | rs2289379 | T | C | 0.39497 | -0.01699 | 0.00244 | 3.50E-12 | 48.39 |
| 97 | rs2292238 | C | A | 0.40867 | -0.01538 | 0.00243 | 2.29E-10 | 40.20 |
| 98 | rs2307111 | C | T | 0.39318 | -0.02791 | 0.00244 | 2.56E-30 | 130.96 |
| 99 | rs2318543 | G | A | 0.78250 | -0.01805 | 0.00289 | 4.44E-10 | 38.91 |
| 100 | rs2404324 | G | A | 0.15487 | -0.02026 | 0.00329 | 7.34E-10 | 37.93 |
| 101 | rs2425024 | C | A | 0.32374 | 0.01654 | 0.00254 | 7.64E-11 | 42.35 |
| 102 | rs2439823 | G | A | 0.54771 | 0.01983 | 0.00240 | 1.28E-16 | 68.48 |
| 103 | rs245775 | G | A | 0.72883 | 0.02131 | 0.00268 | 1.78E-15 | 63.30 |
| 104 | rs2479958 | G | A | 0.51515 | -0.01520 | 0.00241 | 2.70E-10 | 39.88 |
| 105 | rs2494196 | A | C | 0.28766 | 0.01817 | 0.00263 | 4.79E-12 | 47.77 |
| 106 | rs2499468 | A | C | 0.65274 | 0.01591 | 0.00250 | 2.10E-10 | 40.37 |
| 107 | rs252749 | A | G | 0.24657 | -0.01931 | 0.00276 | 2.43E-12 | 49.10 |
| 108 | rs2678204 | G | T | 0.34181 | 0.02563 | 0.00251 | 1.91E-24 | 104.13 |
| 109 | rs273505 | C | T | 0.41991 | 0.02008 | 0.00241 | 8.30E-17 | 69.34 |
| 110 | rs28366156 | C | T | 0.13136 | -0.02839 | 0.00352 | 7.66E-16 | 64.96 |
| 111 | rs2861685 | C | T | 0.41137 | -0.01636 | 0.00241 | 1.26E-11 | 45.88 |
| 112 | rs286818 | A | T | 0.17025 | -0.02281 | 0.00317 | 6.64E-13 | 51.65 |
| 113 | rs2903738 | T | A | 0.22048 | -0.01603 | 0.00287 | 2.37E-08 | 31.16 |
| 114 | rs2954021 | G | A | 0.50596 | 0.01654 | 0.00238 | 3.74E-12 | 48.26 |
| 115 | rs34045288 | T | C | 0.33590 | 0.02299 | 0.00252 | 7.68E-20 | 83.14 |
| 116 | rs34045894 | A | G | 0.15739 | 0.01936 | 0.00327 | 3.10E-09 | 35.12 |
| 117 | rs34292685 | T | C | 0.16209 | -0.01990 | 0.00322 | 6.65E-10 | 38.12 |
| 118 | rs34517439 | A | C | 0.12577 | 0.04070 | 0.00365 | 7.01E-29 | 124.39 |
| 119 | rs34769775 | T | C | 0.29786 | -0.01746 | 0.00260 | 1.80E-11 | 45.18 |
| 120 | rs34966008 | T | C | 0.40815 | -0.01792 | 0.00242 | 1.30E-13 | 54.86 |
| 121 | rs35537311 | T | C | 0.38988 | -0.01381 | 0.00244 | 1.54E-08 | 32.00 |
| 122 | rs35679975 | C | T | 0.19222 | -0.02395 | 0.00303 | 2.68E-15 | 62.49 |
| 123 | rs35851183 | G | A | 0.35778 | 0.01530 | 0.00248 | 7.33E-10 | 37.93 |
| 124 | rs3729793 | G | C | 0.09388 | 0.02240 | 0.00409 | 4.33E-08 | 30.00 |
| 125 | rs3770821 | C | T | 0.33132 | 0.01566 | 0.00254 | 7.48E-10 | 37.89 |
| 126 | rs3803286 | G | A | 0.66638 | -0.01878 | 0.00252 | 9.37E-14 | 55.50 |
| 127 | rs3826408 | T | C | 0.45721 | 0.01451 | 0.00239 | 1.24E-09 | 36.91 |
| 128 | rs4474229 | A | G | 0.37234 | -0.01541 | 0.00246 | 3.69E-10 | 39.27 |
| 129 | rs4482463 | A | C | 0.92418 | -0.03280 | 0.00450 | 3.27E-13 | 53.04 |
| 130 | rs4502882 | T | C | 0.65689 | -0.01582 | 0.00251 | 2.75E-10 | 39.85 |
| 131 | rs4549080 | T | C | 0.34101 | 0.01577 | 0.00251 | 3.34E-10 | 39.47 |
| 132 | rs4673617 | T | C | 0.27164 | 0.01711 | 0.00268 | 1.82E-10 | 40.65 |
| 133 | rs4740383 | A | G | 0.40351 | 0.01345 | 0.00243 | 3.23E-08 | 30.57 |
| 134 | rs4757144 | A | G | 0.59305 | 0.01554 | 0.00242 | 1.39E-10 | 41.17 |
| 135 | rs4776970 | T | A | 0.35707 | -0.02420 | 0.00248 | 1.87E-22 | 95.05 |
| 136 | rs4777541 | T | C | 0.76504 | 0.01948 | 0.00282 | 4.83E-12 | 47.76 |
| 137 | rs4832298 | T | C | 0.68603 | -0.01400 | 0.00256 | 4.76E-08 | 29.81 |
| 138 | rs4856721 | A | G | 0.53865 | 0.01408 | 0.00239 | 3.57E-09 | 34.84 |
| 139 | rs4864201 | C | T | 0.65330 | -0.01542 | 0.00250 | 6.61E-10 | 38.13 |
| 140 | rs4886039 | G | A | 0.17143 | -0.01982 | 0.00316 | 3.50E-10 | 39.38 |
| 141 | rs4921301 | T | C | 0.20982 | -0.01729 | 0.00296 | 4.90E-09 | 34.23 |
| 142 | rs4926726 | A | G | 0.54323 | -0.01489 | 0.00239 | 4.59E-10 | 38.85 |
| 143 | rs4958702 | C | T | 0.57368 | -0.01590 | 0.00240 | 3.73E-11 | 43.76 |
| 144 | rs541577 | G | A | 0.62126 | -0.01496 | 0.00246 | 1.27E-09 | 36.86 |
| 145 | rs543874 | G | A | 0.20781 | 0.04526 | 0.00293 | 7.81E-54 | 238.72 |
| 146 | rs55637757 | T | C | 0.13344 | -0.02153 | 0.00356 | 1.43E-09 | 36.63 |
| 147 | rs55655049 | T | C | 0.23647 | -0.01573 | 0.00280 | 2.03E-08 | 31.46 |
| 148 | rs55726687 | A | G | 0.21114 | 0.02313 | 0.00291 | 2.08E-15 | 62.99 |
| 149 | rs56803094 | G | A | 0.22711 | -0.01906 | 0.00285 | 2.13E-11 | 44.85 |
| 150 | rs57636386 | C | T | 0.08342 | -0.04055 | 0.00431 | 5.21E-21 | 88.46 |
| 151 | rs58084604 | T | C | 0.23441 | 0.05040 | 0.00280 | 3.79E-72 | 322.83 |
| 152 | rs59104534 | T | C | 0.30111 | 0.01435 | 0.00260 | 3.60E-08 | 30.35 |
| 153 | rs60764613 | T | G | 0.14486 | 0.02336 | 0.00339 | 5.85E-12 | 47.38 |
| 154 | rs61813324 | T | C | 0.13488 | 0.02540 | 0.00353 | 6.18E-13 | 51.79 |
| 155 | rs61826865 | A | T | 0.10894 | 0.02098 | 0.00383 | 4.17E-08 | 30.07 |
| 156 | rs61871615 | T | C | 0.09052 | -0.02868 | 0.00433 | 3.57E-11 | 43.84 |
| 157 | rs62037365 | G | C | 0.40213 | 0.03107 | 0.00243 | 1.62E-37 | 163.90 |
| 158 | rs62104483 | A | G | 0.32951 | 0.02320 | 0.00253 | 5.49E-20 | 83.81 |
| 159 | rs62106258 | C | T | 0.04847 | -0.08628 | 0.00554 | 1.20E-54 | 242.46 |
| 160 | rs62178054 | C | T | 0.37189 | -0.01721 | 0.00247 | 3.55E-12 | 48.36 |
| 161 | rs62246314 | A | G | 0.10128 | 0.02526 | 0.00394 | 1.51E-10 | 41.02 |
| 162 | rs62261725 | G | A | 0.32744 | -0.02129 | 0.00254 | 5.18E-17 | 70.27 |
| 163 | rs62343137 | C | G | 0.37069 | 0.01752 | 0.00246 | 1.18E-12 | 50.52 |
| 164 | rs62396185 | C | G | 0.25646 | -0.02876 | 0.00273 | 5.85E-26 | 111.04 |
| 165 | rs62477684 | T | C | 0.41964 | -0.02060 | 0.00241 | 1.30E-17 | 73.00 |
| 166 | rs649648 | G | A | 0.83645 | -0.01845 | 0.00322 | 9.89E-09 | 32.86 |
| 167 | rs6542924 | A | C | 0.68221 | -0.01742 | 0.00257 | 1.29E-11 | 45.82 |
| 168 | rs6601527 | A | C | 0.58782 | -0.01870 | 0.00242 | 1.16E-14 | 59.61 |
| 169 | rs6656785 | G | A | 0.39244 | 0.01512 | 0.00244 | 5.49E-10 | 38.50 |
| 170 | rs66679256 | T | C | 0.44614 | 0.01906 | 0.00240 | 1.93E-15 | 63.15 |
| 171 | rs668799 | T | C | 0.27972 | 0.01728 | 0.00265 | 7.28E-11 | 42.44 |
| 172 | rs6699744 | T | A | 0.61402 | 0.02134 | 0.00245 | 3.45E-18 | 75.62 |
| 173 | rs6739755 | G | A | 0.60298 | -0.01999 | 0.00244 | 2.38E-16 | 67.26 |
| 174 | rs67602344 | A | C | 0.19295 | -0.01789 | 0.00302 | 3.26E-09 | 35.02 |
| 175 | rs6861649 | C | T | 0.60749 | 0.01442 | 0.00245 | 3.75E-09 | 34.75 |
| 176 | rs7030732 | A | C | 0.60789 | -0.01415 | 0.00244 | 6.25E-09 | 33.76 |
| 177 | rs704061 | C | T | 0.45270 | 0.01876 | 0.00239 | 4.28E-15 | 61.57 |
| 178 | rs7094644 | A | G | 0.67418 | 0.01537 | 0.00259 | 2.80E-09 | 35.32 |
| 179 | rs7116641 | G | T | 0.31680 | 0.02439 | 0.00256 | 1.72E-21 | 90.66 |
| 180 | rs7124681 | A | C | 0.40806 | 0.02556 | 0.00242 | 3.96E-26 | 111.81 |
| 181 | rs713586 | C | T | 0.48951 | 0.02982 | 0.00238 | 4.18E-36 | 157.44 |
| 182 | rs7141420 | T | C | 0.51408 | 0.02156 | 0.00240 | 2.34E-19 | 80.94 |
| 183 | rs7201895 | A | G | 0.35598 | -0.01705 | 0.00250 | 9.32E-12 | 46.47 |
| 184 | rs7259070 | C | T | 0.59901 | 0.02042 | 0.00245 | 7.75E-17 | 69.48 |
| 185 | rs72740701 | G | A | 0.28206 | -0.01455 | 0.00265 | 4.04E-08 | 30.13 |
| 186 | rs72892910 | T | G | 0.17044 | 0.03649 | 0.00317 | 1.35E-30 | 132.23 |
| 187 | rs72917533 | C | T | 0.18627 | -0.01742 | 0.00306 | 1.30E-08 | 32.33 |
| 188 | rs73213484 | T | A | 0.13906 | -0.02249 | 0.00344 | 6.25E-11 | 42.74 |
| 189 | rs7442885 | G | C | 0.21010 | -0.02386 | 0.00293 | 3.73E-16 | 66.38 |
| 190 | rs745249 | T | C | 0.28394 | 0.01506 | 0.00265 | 1.33E-08 | 32.29 |
| 191 | rs750090 | C | T | 0.35615 | -0.01570 | 0.00251 | 4.01E-10 | 39.11 |
| 192 | rs756717 | A | G | 0.39886 | -0.01538 | 0.00246 | 3.89E-10 | 39.17 |
| 193 | rs7584391 | A | G | 0.18555 | -0.01722 | 0.00306 | 1.83E-08 | 31.67 |
| 194 | rs76040172 | A | G | 0.05444 | -0.03948 | 0.00526 | 6.09E-14 | 56.35 |
| 195 | rs7649970 | T | C | 0.12087 | 0.02413 | 0.00364 | 3.49E-11 | 43.88 |
| 196 | rs765876 | G | A | 0.48844 | -0.01333 | 0.00238 | 2.06E-08 | 31.44 |
| 197 | rs76895963 | G | T | 0.01928 | 0.07135 | 0.00923 | 1.10E-14 | 59.72 |
| 198 | rs7707394 | A | G | 0.35432 | -0.01906 | 0.00248 | 1.65E-14 | 58.91 |
| 199 | rs7811825 | A | G | 0.13306 | 0.02135 | 0.00351 | 1.14E-09 | 37.07 |
| 200 | rs7845090 | A | G | 0.71112 | -0.02262 | 0.00264 | 1.05E-17 | 73.42 |
| 201 | rs78719460 | A | G | 0.31094 | 0.01420 | 0.00257 | 3.37E-08 | 30.48 |
| 202 | rs79113395 | A | G | 0.26545 | -0.01720 | 0.00271 | 2.07E-10 | 40.41 |
| 203 | rs7915723 | A | C | 0.55876 | -0.01480 | 0.00240 | 6.96E-10 | 38.03 |
| 204 | rs7917983 | C | T | 0.46559 | -0.01304 | 0.00238 | 4.43E-08 | 29.95 |
| 205 | rs7925100 | A | G | 0.39634 | 0.01372 | 0.00243 | 1.68E-08 | 31.83 |
| 206 | rs7930275 | T | C | 0.22125 | 0.01958 | 0.00291 | 1.73E-11 | 45.26 |
| 207 | rs79535757 | T | A | 0.00820 | -0.07476 | 0.01347 | 2.89E-08 | 30.78 |
| 208 | rs7987928 | A | G | 0.79963 | -0.01779 | 0.00297 | 2.21E-09 | 35.78 |
| 209 | rs801738 | G | C | 0.35841 | -0.01651 | 0.00248 | 2.85E-11 | 44.28 |
| 210 | rs8026927 | A | C | 0.36361 | -0.01385 | 0.00249 | 2.73E-08 | 30.89 |
| 211 | rs8087074 | T | G | 0.26112 | 0.01757 | 0.00272 | 1.07E-10 | 41.69 |
| 212 | rs815163 | C | T | 0.56160 | -0.01656 | 0.00240 | 4.97E-12 | 47.70 |
| 213 | rs8192675 | C | T | 0.28738 | 0.01770 | 0.00263 | 1.57E-11 | 45.45 |
| 214 | rs845084 | A | G | 0.25755 | 0.01833 | 0.00273 | 1.92E-11 | 45.06 |
| 215 | rs846545 | C | T | 0.42032 | -0.01472 | 0.00242 | 1.09E-09 | 37.15 |
| 216 | rs854917 | T | C | 0.73801 | -0.01662 | 0.00272 | 9.94E-10 | 37.34 |
| 217 | rs862320 | T | C | 0.41015 | -0.02003 | 0.00242 | 1.38E-16 | 68.34 |
| 218 | rs879620 | T | C | 0.61528 | 0.02651 | 0.00245 | 3.52E-27 | 116.62 |
| 219 | rs9289630 | C | G | 0.39076 | 0.01972 | 0.00245 | 8.06E-16 | 64.86 |
| 220 | rs9291822 | T | C | 0.51546 | -0.01387 | 0.00240 | 7.82E-09 | 33.32 |
| 221 | rs9320823 | C | T | 0.60190 | 0.01799 | 0.00243 | 1.46E-13 | 54.62 |
| 222 | rs935166 | A | G | 0.50694 | -0.01604 | 0.00238 | 1.60E-11 | 45.42 |
| 223 | rs9375702 | T | C | 0.69033 | -0.01607 | 0.00257 | 4.29E-10 | 38.98 |
| 224 | rs947088 | T | G | 0.71836 | 0.01476 | 0.00265 | 2.68E-08 | 30.92 |
| 225 | rs9549099 | C | T | 0.26409 | 0.01551 | 0.00271 | 9.96E-09 | 32.85 |
| 226 | rs9688977 | C | T | 0.14649 | 0.02334 | 0.00337 | 4.67E-12 | 47.83 |
| 227 | rs9835772 | T | A | 0.24276 | 0.01637 | 0.00277 | 3.60E-09 | 34.83 |
| 228 | rs9843007 | T | A | 0.37540 | -0.01458 | 0.00246 | 2.90E-09 | 35.25 |
| 229 | rs9847186 | A | G | 0.42774 | -0.01521 | 0.00241 | 2.82E-10 | 39.80 |
| 230 | rs9861443 | C | A | 0.71366 | 0.01577 | 0.00265 | 2.62E-09 | 35.45 |
| 231 | rs9883422 | G | A | 0.27873 | -0.01463 | 0.00267 | 4.17E-08 | 30.07 |
| 232 | rs9968060 | T | C | 0.64661 | 0.01550 | 0.00253 | 9.16E-10 | 37.50 |

SNPs, single nucleotide polymorphisms; EAF, effect allele frequency; SE, standard error.

**Supplementary Table 5: Characteristics of 242 SNPs used as instrumental variables for the effect of Right leg fat mass on asthma.**

|  | SNP | effect_allele.exposure | other_allele.exposure | EAF.exposure | effect | SE | *P*-value | F-Statistic |
| --- | --- | --- | --- | --- | --- | --- | --- | --- |
| 1 | rs10038105 | G | T | 0.72896 | 0.01527 | 0.00218 | 2.66E-12 | 48.93 |
| 2 | rs10100245 | A | G | 0.56644 | 0.01506 | 0.00196 | 1.51E-14 | 59.09 |
| 3 | rs10128597 | A | G | 0.27402 | -0.01450 | 0.00219 | 3.56E-11 | 43.85 |
| 4 | rs10209821 | T | C | 0.34109 | 0.01500 | 0.00205 | 2.36E-13 | 53.69 |
| 5 | rs10215731 | C | T | 0.26496 | 0.01207 | 0.00221 | 4.57E-08 | 29.89 |
| 6 | rs10237306 | T | G | 0.38022 | 0.01223 | 0.00200 | 9.48E-10 | 37.43 |
| 7 | rs10269774 | A | G | 0.32354 | 0.01167 | 0.00208 | 1.93E-08 | 31.56 |
| 8 | rs10423928 | A | T | 0.19349 | -0.02568 | 0.00245 | 1.08E-25 | 109.83 |
| 9 | rs10490530 | G | A | 0.13916 | 0.01579 | 0.00281 | 1.91E-08 | 31.58 |
| 10 | rs10499014 | G | C | 0.26787 | -0.01484 | 0.00221 | 1.78E-11 | 45.20 |
| 11 | rs10756714 | G | A | 0.44479 | -0.01808 | 0.00196 | 2.46E-20 | 85.39 |
| 12 | rs10756798 | T | C | 0.64503 | -0.01478 | 0.00203 | 3.34E-13 | 53.00 |
| 13 | rs10787738 | T | C | 0.25330 | 0.01660 | 0.00226 | 2.21E-13 | 53.81 |
| 14 | rs10827289 | T | C | 0.58303 | -0.01196 | 0.00197 | 1.39E-09 | 36.69 |
| 15 | rs10835186 | A | G | 0.74176 | 0.01285 | 0.00222 | 7.59E-09 | 33.38 |
| 16 | rs10883553 | A | C | 0.44653 | 0.01148 | 0.00196 | 4.27E-09 | 34.50 |
| 17 | rs10938397 | G | A | 0.43408 | 0.02206 | 0.00196 | 2.71E-29 | 126.27 |
| 18 | rs10954772 | C | T | 0.68571 | -0.01439 | 0.00210 | 8.17E-12 | 46.73 |
| 19 | rs10995427 | A | G | 0.36083 | -0.01217 | 0.00203 | 2.16E-09 | 35.83 |
| 20 | rs11100870 | G | T | 0.54958 | -0.01212 | 0.00195 | 5.10E-10 | 38.64 |
| 21 | rs11150745 | G | A | 0.31949 | -0.01702 | 0.00208 | 3.29E-16 | 66.63 |
| 22 | rs111955609 | A | G | 0.08815 | 0.02017 | 0.00344 | 4.48E-09 | 34.40 |
| 23 | rs11223641 | C | T | 0.14462 | -0.01571 | 0.00278 | 1.50E-08 | 32.05 |
| 24 | rs11242730 | T | G | 0.24003 | 0.01264 | 0.00228 | 2.91E-08 | 30.77 |
| 25 | rs1127100 | C | T | 0.64824 | 0.01507 | 0.00204 | 1.45E-13 | 54.64 |
| 26 | rs113230003 | A | G | 0.26019 | -0.01430 | 0.00223 | 1.44E-10 | 41.11 |
| 27 | rs113866544 | C | T | 0.06768 | 0.02823 | 0.00387 | 2.99E-13 | 53.22 |
| 28 | rs115584674 | G | A | 0.05946 | -0.02370 | 0.00410 | 7.45E-09 | 33.42 |
| 29 | rs11611780 | C | G | 0.25432 | 0.01261 | 0.00223 | 1.58E-08 | 31.95 |
| 30 | rs116399833 | A | C | 0.22905 | 0.01294 | 0.00236 | 4.08E-08 | 30.11 |
| 31 | rs11642015 | T | C | 0.40236 | 0.05195 | 0.00198 | 9.53E-121 | 689.60 |
| 32 | rs11656758 | G | A | 0.33962 | 0.01199 | 0.00205 | 5.12E-09 | 34.15 |
| 33 | rs11668971 | C | T | 0.48114 | -0.01505 | 0.00194 | 8.81E-15 | 60.15 |
| 34 | rs11695700 | T | G | 0.41767 | 0.01126 | 0.00198 | 1.21E-08 | 32.47 |
| 35 | rs117342986 | T | C | 0.02672 | 0.03453 | 0.00632 | 4.55E-08 | 29.90 |
| 36 | rs11742930 | T | C | 0.56643 | 0.01267 | 0.00196 | 1.07E-10 | 41.69 |
| 37 | rs11782341 | G | A | 0.18851 | 0.01381 | 0.00250 | 3.32E-08 | 30.51 |
| 38 | rs11824092 | C | T | 0.63745 | 0.01257 | 0.00203 | 6.03E-10 | 38.31 |
| 39 | rs12001634 | A | T | 0.33642 | -0.01217 | 0.00206 | 3.24E-09 | 35.03 |
| 40 | rs12034227 | C | A | 0.33733 | -0.01247 | 0.00206 | 1.36E-09 | 36.73 |
| 41 | rs12049202 | T | C | 0.19865 | 0.02170 | 0.00243 | 4.49E-19 | 79.65 |
| 42 | rs12140153 | T | G | 0.09676 | -0.02723 | 0.00337 | 6.18E-16 | 65.38 |
| 43 | rs12144626 | C | T | 0.58182 | -0.01210 | 0.00198 | 9.16E-10 | 37.50 |
| 44 | rs1225004 | C | T | 0.27798 | 0.01779 | 0.00217 | 2.42E-16 | 67.23 |
| 45 | rs12254441 | T | C | 0.37284 | -0.01250 | 0.00208 | 1.77E-09 | 36.21 |
| 46 | rs12273545 | T | C | 0.05580 | 0.02383 | 0.00423 | 1.78E-08 | 31.72 |
| 47 | rs1229984 | C | T | 0.97755 | 0.04248 | 0.00656 | 9.37E-11 | 41.95 |
| 48 | rs12339822 | G | A | 0.54320 | 0.01310 | 0.00196 | 2.22E-11 | 44.77 |
| 49 | rs12439200 | A | G | 0.23852 | -0.01690 | 0.00229 | 1.46E-13 | 54.63 |
| 50 | rs12475388 | A | G | 0.48675 | -0.01147 | 0.00195 | 4.21E-09 | 34.53 |
| 51 | rs12477385 | T | G | 0.22730 | -0.01509 | 0.00232 | 8.31E-11 | 42.19 |
| 52 | rs12479357 | G | A | 0.63108 | 0.01537 | 0.00202 | 2.75E-14 | 57.91 |
| 53 | rs12578952 | G | A | 0.25170 | -0.01512 | 0.00224 | 1.41E-11 | 45.66 |
| 54 | rs12583517 | T | G | 0.23022 | -0.01624 | 0.00231 | 1.97E-12 | 49.52 |
| 55 | rs12583872 | C | A | 0.30973 | -0.01151 | 0.00210 | 4.13E-08 | 30.09 |
| 56 | rs12610925 | G | A | 0.35177 | 0.01308 | 0.00204 | 1.37E-10 | 41.21 |
| 57 | rs12619178 | T | C | 0.40292 | -0.01492 | 0.00198 | 4.80E-14 | 56.81 |
| 58 | rs12679106 | T | G | 0.71134 | -0.01967 | 0.00215 | 6.67E-20 | 83.42 |
| 59 | rs12680342 | G | T | 0.22910 | -0.01422 | 0.00231 | 7.09E-10 | 38.00 |
| 60 | rs12681792 | A | C | 0.19381 | 0.01380 | 0.00246 | 2.12E-08 | 31.38 |
| 61 | rs12714415 | C | T | 0.16687 | -0.03802 | 0.00267 | 7.07E-46 | 202.21 |
| 62 | rs12756213 | A | G | 0.61810 | 0.01215 | 0.00200 | 1.20E-09 | 36.97 |
| 63 | rs1286065 | T | C | 0.70270 | 0.01164 | 0.00213 | 4.35E-08 | 29.99 |
| 64 | rs12877270 | A | G | 0.43845 | 0.01231 | 0.00197 | 4.34E-10 | 38.96 |
| 65 | rs12890931 | G | T | 0.36346 | 0.01205 | 0.00203 | 2.97E-09 | 35.21 |
| 66 | rs12891263 | A | G | 0.14317 | -0.01575 | 0.00278 | 1.39E-08 | 32.20 |
| 67 | rs1296328 | C | A | 0.55991 | -0.01525 | 0.00196 | 8.49E-15 | 60.22 |
| 68 | rs13062093 | G | T | 0.36644 | 0.01494 | 0.00201 | 1.16E-13 | 55.09 |
| 69 | rs13135092 | G | A | 0.08323 | 0.03085 | 0.00354 | 2.75E-18 | 76.07 |
| 70 | rs13333747 | C | T | 0.18196 | -0.01801 | 0.00253 | 1.05E-12 | 50.75 |
| 71 | rs1335055 | A | G | 0.65578 | -0.01210 | 0.00206 | 4.50E-09 | 34.40 |
| 72 | rs13410783 | G | A | 0.36835 | 0.01270 | 0.00202 | 3.01E-10 | 39.67 |
| 73 | rs13427822 | G | A | 0.27205 | -0.01501 | 0.00221 | 1.01E-11 | 46.32 |
| 74 | rs1379828 | T | C | 0.79816 | -0.01525 | 0.00242 | 3.16E-10 | 39.57 |
| 75 | rs1412239 | G | C | 0.32454 | 0.01823 | 0.00207 | 1.34E-18 | 77.49 |
| 76 | rs1441264 | A | G | 0.59187 | 0.01477 | 0.00202 | 2.44E-13 | 53.62 |
| 77 | rs1446585 | G | A | 0.22801 | -0.01453 | 0.00232 | 4.12E-10 | 39.06 |
| 78 | rs1458156 | T | C | 0.48826 | 0.01244 | 0.00194 | 1.55E-10 | 40.97 |
| 79 | rs147730268 | T | G | 0.09053 | -0.03303 | 0.00350 | 3.68E-21 | 89.15 |
| 80 | rs1491905 | C | T | 0.50632 | -0.01204 | 0.00194 | 5.55E-10 | 38.48 |
| 81 | rs1503527 | T | C | 0.47972 | 0.01345 | 0.00194 | 4.39E-12 | 47.94 |
| 82 | rs1524445 | T | C | 0.42041 | -0.01426 | 0.00198 | 6.19E-13 | 51.79 |
| 83 | rs1538742 | A | C | 0.56959 | -0.01342 | 0.00197 | 9.14E-12 | 46.51 |
| 84 | rs1566085 | T | G | 0.54544 | -0.01196 | 0.00196 | 1.15E-09 | 37.05 |
| 85 | rs16916303 | G | A | 0.11923 | -0.01661 | 0.00302 | 3.83E-08 | 30.24 |
| 86 | rs16996657 | C | T | 0.12816 | 0.01619 | 0.00291 | 2.62E-08 | 30.97 |
| 87 | rs17024393 | C | T | 0.02580 | 0.04898 | 0.00614 | 1.57E-15 | 63.55 |
| 88 | rs17149254 | C | T | 0.80856 | -0.01702 | 0.00252 | 1.41E-11 | 45.66 |
| 89 | rs17522122 | T | G | 0.47032 | 0.01402 | 0.00195 | 6.66E-13 | 51.65 |
| 90 | rs17627049 | A | C | 0.27425 | 0.01483 | 0.00218 | 9.18E-12 | 46.50 |
| 91 | rs17639996 | A | G | 0.15138 | -0.01691 | 0.00272 | 5.04E-10 | 38.66 |
| 92 | rs17739093 | C | T | 0.03469 | -0.02987 | 0.00530 | 1.77E-08 | 31.73 |
| 93 | rs1782508 | G | C | 0.65633 | -0.01238 | 0.00204 | 1.31E-09 | 36.79 |
| 94 | rs1788808 | G | A | 0.49734 | -0.01793 | 0.00194 | 2.98E-20 | 85.01 |
| 95 | rs1840126 | C | A | 0.56206 | -0.01078 | 0.00196 | 3.65E-08 | 30.33 |
| 96 | rs1928496 | T | C | 0.74239 | 0.01475 | 0.00222 | 2.89E-11 | 44.25 |
| 97 | rs1964675 | T | C | 0.71158 | 0.01253 | 0.00215 | 5.37E-09 | 34.05 |
| 98 | rs2016469 | A | G | 0.37370 | 0.01239 | 0.00202 | 9.16E-10 | 37.50 |
| 99 | rs208477 | T | C | 0.18643 | 0.01458 | 0.00249 | 4.89E-09 | 34.23 |
| 100 | rs2102278 | G | A | 0.32208 | 0.01206 | 0.00208 | 7.03E-09 | 33.53 |
| 101 | rs2155869 | C | T | 0.81916 | -0.01436 | 0.00252 | 1.14E-08 | 32.59 |
| 102 | rs2172131 | C | T | 0.58064 | -0.01431 | 0.00197 | 3.59E-13 | 52.86 |
| 103 | rs2181375 | G | A | 0.59863 | 0.01474 | 0.00198 | 9.42E-14 | 55.49 |
| 104 | rs2292238 | C | A | 0.40867 | -0.01224 | 0.00198 | 6.09E-10 | 38.29 |
| 105 | rs2307111 | C | T | 0.39318 | -0.02322 | 0.00199 | 1.81E-31 | 136.22 |
| 106 | rs2318543 | G | A | 0.78250 | -0.01457 | 0.00236 | 6.78E-10 | 38.08 |
| 107 | rs2370982 | T | C | 0.21534 | 0.02317 | 0.00238 | 2.18E-22 | 94.74 |
| 108 | rs2398861 | G | A | 0.25660 | 0.01567 | 0.00223 | 2.20E-12 | 49.30 |
| 109 | rs241459 | C | A | 0.68028 | -0.01794 | 0.00208 | 5.90E-18 | 74.56 |
| 110 | rs2418507 | T | C | 0.57421 | 0.01274 | 0.00197 | 9.91E-11 | 41.84 |
| 111 | rs2439823 | G | A | 0.54771 | 0.01590 | 0.00196 | 4.33E-16 | 66.08 |
| 112 | rs2455386 | G | A | 0.67961 | -0.01144 | 0.00209 | 4.40E-08 | 29.97 |
| 113 | rs252749 | A | G | 0.24657 | -0.01481 | 0.00225 | 4.50E-11 | 43.39 |
| 114 | rs2606228 | C | A | 0.64277 | -0.01175 | 0.00205 | 9.97E-09 | 32.85 |
| 115 | rs2678204 | G | T | 0.34181 | 0.02041 | 0.00205 | 2.26E-23 | 99.24 |
| 116 | rs2737251 | T | C | 0.33800 | -0.01596 | 0.00205 | 7.58E-15 | 60.45 |
| 117 | rs27741 | A | G | 0.41894 | 0.02152 | 0.00197 | 9.09E-28 | 119.30 |
| 118 | rs2812757 | A | G | 0.23500 | 0.01255 | 0.00230 | 4.74E-08 | 29.82 |
| 119 | rs28366156 | C | T | 0.13136 | -0.02315 | 0.00287 | 7.93E-16 | 64.89 |
| 120 | rs28414958 | A | G | 0.06987 | 0.02209 | 0.00382 | 7.33E-09 | 33.45 |
| 121 | rs28457808 | G | C | 0.16319 | -0.01652 | 0.00263 | 3.47E-10 | 39.39 |
| 122 | rs2861685 | C | T | 0.41137 | -0.01373 | 0.00197 | 3.24E-12 | 48.54 |
| 123 | rs286818 | A | T | 0.17025 | -0.01938 | 0.00259 | 7.22E-14 | 56.01 |
| 124 | rs28726372 | C | T | 0.30781 | 0.01202 | 0.00210 | 1.11E-08 | 32.65 |
| 125 | rs2917705 | A | G | 0.13939 | -0.01626 | 0.00281 | 6.96E-09 | 33.55 |
| 126 | rs2954021 | G | A | 0.50596 | 0.01224 | 0.00194 | 3.00E-10 | 39.68 |
| 127 | rs329118 | T | C | 0.41819 | -0.01102 | 0.00197 | 2.20E-08 | 31.31 |
| 128 | rs34045288 | T | C | 0.33590 | 0.01974 | 0.00206 | 8.48E-22 | 92.06 |
| 129 | rs34769775 | T | C | 0.29786 | -0.01559 | 0.00212 | 1.93E-13 | 54.08 |
| 130 | rs34966008 | T | C | 0.40815 | -0.01561 | 0.00197 | 2.65E-15 | 62.52 |
| 131 | rs35025195 | A | G | 0.16613 | -0.01994 | 0.00261 | 2.22E-14 | 58.34 |
| 132 | rs35154326 | G | A | 0.27246 | -0.01358 | 0.00220 | 6.38E-10 | 38.20 |
| 133 | rs3749472 | G | A | 0.46158 | 0.01101 | 0.00195 | 1.60E-08 | 31.93 |
| 134 | rs3784699 | C | T | 0.60302 | 0.01939 | 0.00199 | 1.62E-22 | 95.33 |
| 135 | rs3803286 | G | A | 0.66638 | -0.01722 | 0.00206 | 5.75E-17 | 70.07 |
| 136 | rs3897102 | T | C | 0.41225 | 0.01244 | 0.00199 | 3.96E-10 | 39.13 |
| 137 | rs444102 | C | G | 0.17110 | 0.01556 | 0.00258 | 1.62E-09 | 36.38 |
| 138 | rs4467770 | A | G | 0.73067 | 0.01526 | 0.00220 | 3.72E-12 | 48.27 |
| 139 | rs4471907 | A | G | 0.52876 | 0.01153 | 0.00195 | 3.14E-09 | 35.10 |
| 140 | rs4482463 | A | C | 0.92418 | -0.02619 | 0.00368 | 1.02E-12 | 50.80 |
| 141 | rs4502882 | T | C | 0.65689 | -0.01339 | 0.00204 | 5.85E-11 | 42.87 |
| 142 | rs4503172 | T | C | 0.60782 | -0.01240 | 0.00199 | 4.57E-10 | 38.85 |
| 143 | rs4648450 | A | C | 0.46613 | -0.01132 | 0.00196 | 7.49E-09 | 33.40 |
| 144 | rs4657796 | C | T | 0.37656 | -0.01175 | 0.00201 | 4.95E-09 | 34.21 |
| 145 | rs4673617 | T | C | 0.27164 | 0.01363 | 0.00219 | 4.79E-10 | 38.76 |
| 146 | rs4682711 | C | T | 0.40868 | -0.01189 | 0.00197 | 1.67E-09 | 36.33 |
| 147 | rs4718964 | T | G | 0.41219 | 0.01389 | 0.00198 | 2.17E-12 | 49.33 |
| 148 | rs4958702 | C | T | 0.57368 | -0.01315 | 0.00196 | 2.02E-11 | 44.95 |
| 149 | rs4964630 | A | G | 0.76078 | 0.01309 | 0.00229 | 1.04E-08 | 32.78 |
| 150 | rs4981693 | A | G | 0.77273 | 0.01735 | 0.00232 | 7.51E-14 | 55.93 |
| 151 | rs525101 | C | T | 0.37196 | 0.01426 | 0.00201 | 1.41E-12 | 50.18 |
| 152 | rs541577 | G | A | 0.62126 | -0.01383 | 0.00201 | 5.98E-12 | 47.34 |
| 153 | rs543874 | G | A | 0.20781 | 0.03263 | 0.00239 | 2.10E-42 | 186.29 |
| 154 | rs55637757 | T | C | 0.13344 | -0.01684 | 0.00290 | 6.52E-09 | 33.68 |
| 155 | rs55714539 | C | A | 0.34269 | 0.01832 | 0.00206 | 5.59E-19 | 79.22 |
| 156 | rs55726687 | A | G | 0.21114 | 0.01801 | 0.00238 | 3.65E-14 | 57.35 |
| 157 | rs55745410 | G | A | 0.35429 | -0.01142 | 0.00204 | 2.24E-08 | 31.28 |
| 158 | rs55766788 | T | C | 0.11518 | 0.01732 | 0.00304 | 1.28E-08 | 32.36 |
| 159 | rs56803094 | G | A | 0.22711 | -0.01591 | 0.00232 | 7.41E-12 | 46.92 |
| 160 | rs57636386 | C | T | 0.08342 | -0.03107 | 0.00352 | 1.02E-18 | 78.03 |
| 161 | rs58084604 | T | C | 0.23441 | 0.03827 | 0.00229 | 9.90E-63 | 279.57 |
| 162 | rs58862095 | T | C | 0.41976 | -0.01580 | 0.00197 | 1.16E-15 | 64.15 |
| 163 | rs59066241 | G | T | 0.11827 | 0.01789 | 0.00302 | 3.21E-09 | 35.06 |
| 164 | rs59104534 | T | C | 0.30111 | 0.01302 | 0.00213 | 8.96E-10 | 37.54 |
| 165 | rs59227842 | G | A | 0.31243 | 0.01923 | 0.00212 | 9.92E-20 | 82.64 |
| 166 | rs59815219 | T | C | 0.49604 | 0.01071 | 0.00194 | 3.54E-08 | 30.39 |
| 167 | rs6050446 | G | A | 0.96680 | 0.03560 | 0.00546 | 7.10E-11 | 42.49 |
| 168 | rs60764613 | T | G | 0.14486 | 0.01630 | 0.00277 | 3.92E-09 | 34.67 |
| 169 | rs6096886 | G | A | 0.19005 | -0.02089 | 0.00248 | 3.22E-17 | 71.21 |
| 170 | rs6142050 | A | G | 0.58008 | 0.01251 | 0.00197 | 2.21E-10 | 40.27 |
| 171 | rs61765650 | G | A | 0.19246 | -0.02577 | 0.00246 | 1.19E-25 | 109.63 |
| 172 | rs61813324 | T | C | 0.13488 | 0.01863 | 0.00288 | 9.87E-11 | 41.85 |
| 173 | rs61826865 | A | T | 0.10894 | 0.01861 | 0.00312 | 2.49E-09 | 35.55 |
| 174 | rs61888762 | G | C | 0.32201 | 0.02491 | 0.00208 | 3.83E-33 | 143.88 |
| 175 | rs62104477 | T | G | 0.32956 | 0.01728 | 0.00207 | 7.10E-17 | 69.65 |
| 176 | rs62147189 | G | T | 0.62200 | -0.01113 | 0.00203 | 4.33E-08 | 29.99 |
| 177 | rs62246314 | A | G | 0.10128 | 0.01906 | 0.00322 | 3.19E-09 | 35.06 |
| 178 | rs62261725 | G | A | 0.32744 | -0.01958 | 0.00207 | 3.48E-21 | 89.26 |
| 179 | rs62396185 | C | G | 0.25646 | -0.02624 | 0.00223 | 4.82E-32 | 138.85 |
| 180 | rs62444907 | T | C | 0.15303 | -0.01714 | 0.00270 | 2.37E-10 | 40.14 |
| 181 | rs6534626 | T | C | 0.40676 | 0.01216 | 0.00198 | 7.57E-10 | 37.87 |
| 182 | rs6601527 | A | C | 0.58782 | -0.01325 | 0.00198 | 2.07E-11 | 44.90 |
| 183 | rs66679256 | T | C | 0.44614 | 0.01410 | 0.00196 | 5.76E-13 | 51.93 |
| 184 | rs66954327 | G | A | 0.33615 | 0.01201 | 0.00206 | 5.73E-09 | 33.93 |
| 185 | rs6717858 | C | T | 0.40340 | 0.01406 | 0.00198 | 1.30E-12 | 50.33 |
| 186 | rs6739755 | G | A | 0.60298 | -0.01647 | 0.00199 | 1.22E-16 | 68.58 |
| 187 | rs6754640 | A | G | 0.38416 | -0.01184 | 0.00201 | 3.62E-09 | 34.82 |
| 188 | rs67602344 | A | C | 0.19295 | -0.01743 | 0.00247 | 1.60E-12 | 49.93 |
| 189 | rs6861649 | C | T | 0.60749 | 0.01374 | 0.00200 | 5.79E-12 | 47.40 |
| 190 | rs6875585 | C | A | 0.67000 | 0.01238 | 0.00207 | 2.27E-09 | 35.73 |
| 191 | rs6898357 | G | A | 0.68048 | -0.01180 | 0.00208 | 1.41E-08 | 32.17 |
| 192 | rs7027304 | T | C | 0.65245 | 0.01225 | 0.00205 | 2.18E-09 | 35.81 |
| 193 | rs704061 | C | T | 0.45270 | 0.01375 | 0.00195 | 1.86E-12 | 49.63 |
| 194 | rs7094644 | A | G | 0.67418 | 0.01348 | 0.00211 | 1.65E-10 | 40.84 |
| 195 | rs7124681 | A | C | 0.40806 | 0.01839 | 0.00197 | 1.11E-20 | 86.97 |
| 196 | rs7132908 | A | G | 0.38392 | 0.02184 | 0.00200 | 8.51E-28 | 119.43 |
| 197 | rs713586 | C | T | 0.48951 | 0.02285 | 0.00194 | 4.93E-32 | 138.81 |
| 198 | rs7193783 | C | A | 0.64917 | -0.01374 | 0.00204 | 1.81E-11 | 45.17 |
| 199 | rs724016 | G | A | 0.44721 | 0.01552 | 0.00195 | 1.91E-15 | 63.16 |
| 200 | rs7259070 | C | T | 0.59901 | 0.01605 | 0.00200 | 1.00E-15 | 64.43 |
| 201 | rs72820274 | A | G | 0.41876 | 0.01236 | 0.00197 | 3.62E-10 | 39.31 |
| 202 | rs72892910 | T | G | 0.17044 | 0.02974 | 0.00259 | 1.63E-30 | 131.86 |
| 203 | rs72917533 | C | T | 0.18627 | -0.01451 | 0.00250 | 6.59E-09 | 33.65 |
| 204 | rs73035223 | G | A | 0.14854 | 0.01676 | 0.00274 | 9.18E-10 | 37.49 |
| 205 | rs73121277 | C | T | 0.27953 | 0.01202 | 0.00217 | 2.97E-08 | 30.73 |
| 206 | rs73213484 | T | A | 0.13906 | -0.01837 | 0.00281 | 5.97E-11 | 42.83 |
| 207 | rs7442885 | G | C | 0.21010 | -0.02135 | 0.00239 | 4.07E-19 | 79.84 |
| 208 | rs7498044 | A | G | 0.21866 | -0.01349 | 0.00237 | 1.25E-08 | 32.40 |
| 209 | rs750090 | C | T | 0.35615 | -0.01204 | 0.00205 | 4.21E-09 | 34.53 |
| 210 | rs75152244 | G | A | 0.13681 | 0.01569 | 0.00285 | 3.81E-08 | 30.25 |
| 211 | rs75557510 | G | A | 0.06150 | -0.02972 | 0.00413 | 5.87E-13 | 51.89 |
| 212 | rs75641275 | C | A | 0.14395 | 0.01703 | 0.00277 | 7.46E-10 | 37.90 |
| 213 | rs76040172 | A | G | 0.05444 | -0.03259 | 0.00429 | 3.09E-14 | 57.68 |
| 214 | rs7649970 | T | C | 0.12087 | 0.02329 | 0.00297 | 4.63E-15 | 61.42 |
| 215 | rs76895963 | G | T | 0.01928 | 0.05691 | 0.00753 | 4.23E-14 | 57.06 |
| 216 | rs7707394 | A | G | 0.35432 | -0.01585 | 0.00203 | 5.11E-15 | 61.22 |
| 217 | rs77165542 | T | C | 0.03575 | -0.06842 | 0.00531 | 5.99E-38 | 165.88 |
| 218 | rs7728095 | G | A | 0.38802 | 0.01382 | 0.00201 | 5.94E-12 | 47.35 |
| 219 | rs7774 | A | C | 0.30914 | 0.01291 | 0.00211 | 1.02E-09 | 37.28 |
| 220 | rs78565420 | T | C | 0.05419 | 0.02499 | 0.00441 | 1.46E-08 | 32.11 |
| 221 | rs79113395 | A | G | 0.26545 | -0.01408 | 0.00221 | 1.84E-10 | 40.63 |
| 222 | rs7925100 | A | G | 0.39634 | 0.01205 | 0.00198 | 1.27E-09 | 36.87 |
| 223 | rs7933085 | G | A | 0.50920 | 0.01239 | 0.00195 | 2.19E-10 | 40.30 |
| 224 | rs799449 | T | C | 0.55848 | 0.01459 | 0.00196 | 1.04E-13 | 55.30 |
| 225 | rs801738 | G | C | 0.35841 | -0.01643 | 0.00202 | 4.82E-16 | 65.88 |
| 226 | rs8078135 | T | C | 0.48847 | -0.01221 | 0.00195 | 3.80E-10 | 39.21 |
| 227 | rs815163 | C | T | 0.56160 | -0.01269 | 0.00196 | 8.71E-11 | 42.09 |
| 228 | rs8192675 | C | T | 0.28738 | 0.01327 | 0.00214 | 5.78E-10 | 38.40 |
| 229 | rs869400 | G | T | 0.81583 | 0.01748 | 0.00251 | 3.41E-12 | 48.44 |
| 230 | rs879620 | T | C | 0.61528 | 0.02062 | 0.00200 | 7.57E-25 | 105.96 |
| 231 | rs882378 | C | A | 0.30937 | 0.01222 | 0.00211 | 6.76E-09 | 33.60 |
| 232 | rs9320823 | C | T | 0.60190 | 0.01723 | 0.00199 | 4.13E-18 | 75.26 |
| 233 | rs9375702 | T | C | 0.69033 | -0.01346 | 0.00210 | 1.47E-10 | 41.07 |
| 234 | rs9387640 | T | C | 0.36365 | -0.01120 | 0.00202 | 2.84E-08 | 30.81 |
| 235 | rs9584855 | G | T | 0.29010 | -0.01253 | 0.00214 | 4.60E-09 | 34.35 |
| 236 | rs9610311 | C | T | 0.30652 | 0.01251 | 0.00216 | 7.16E-09 | 33.49 |
| 237 | rs9688977 | C | T | 0.14649 | 0.01729 | 0.00275 | 3.39E-10 | 39.44 |
| 238 | rs9835772 | T | A | 0.24276 | 0.01438 | 0.00226 | 2.11E-10 | 40.37 |
| 239 | rs9839081 | A | G | 0.32639 | -0.01182 | 0.00211 | 2.01E-08 | 31.49 |
| 240 | rs9847186 | A | G | 0.42774 | -0.01100 | 0.00197 | 2.26E-08 | 31.26 |
| 241 | rs9861443 | C | A | 0.71366 | 0.01404 | 0.00216 | 8.22E-11 | 42.21 |
| 242 | rs9863890 | G | A | 0.40860 | -0.01181 | 0.00197 | 2.14E-09 | 35.84 |

SNPs, single nucleotide polymorphisms; EAF, effect allele frequency; SE, standard error.

**Supplementary Table 6: Characteristics of 240 SNPs used as instrumental variables for the effect of Trunk fat mass on asthma.**

|  | SNP | effect_allele.exposure | other_allele.exposure | EAF.exposure | effect | SE | *P*-value | F-Statistic |
| --- | --- | --- | --- | --- | --- | --- | --- | --- |
| 1 | rs10100245 | A | G | 0.56644 | 0.02037 | 0.00247 | 1.46E-16 | 68.23 |
| 2 | rs1013293 | A | G | 0.43334 | -0.01984 | 0.00248 | 1.17E-15 | 64.13 |
| 3 | rs10172196 | A | G | 0.30484 | 0.02044 | 0.00266 | 1.69E-14 | 58.87 |
| 4 | rs1017529 | A | C | 0.17856 | 0.01870 | 0.00326 | 9.61E-09 | 32.92 |
| 5 | rs10237306 | T | G | 0.38022 | 0.01716 | 0.00252 | 9.41E-12 | 46.45 |
| 6 | rs10264581 | A | G | 0.93856 | -0.02975 | 0.00509 | 5.11E-09 | 34.15 |
| 7 | rs10269774 | A | G | 0.32354 | 0.02071 | 0.00262 | 2.41E-15 | 62.70 |
| 8 | rs10423928 | A | T | 0.19349 | -0.02994 | 0.00309 | 2.92E-22 | 94.17 |
| 9 | rs10499014 | G | C | 0.26787 | -0.01525 | 0.00278 | 4.09E-08 | 30.11 |
| 10 | rs10756714 | G | A | 0.44479 | -0.02349 | 0.00246 | 1.56E-21 | 90.86 |
| 11 | rs10756798 | T | C | 0.64503 | -0.01821 | 0.00256 | 1.07E-12 | 50.72 |
| 12 | rs10830566 | G | A | 0.27819 | -0.01551 | 0.00273 | 1.34E-08 | 32.27 |
| 13 | rs10863753 | G | A | 0.38303 | -0.01416 | 0.00251 | 1.74E-08 | 31.77 |
| 14 | rs10915846 | A | G | 0.27152 | -0.01589 | 0.00275 | 7.84E-09 | 33.32 |
| 15 | rs10938398 | A | G | 0.43334 | 0.02599 | 0.00247 | 8.35E-26 | 110.33 |
| 16 | rs10999460 | T | C | 0.26595 | 0.01854 | 0.00278 | 2.50E-11 | 44.54 |
| 17 | rs11023199 | G | A | 0.35352 | -0.01442 | 0.00256 | 1.84E-08 | 31.66 |
| 18 | rs11030119 | A | G | 0.30881 | 0.03029 | 0.00265 | 2.60E-30 | 130.93 |
| 19 | rs11042030 | C | T | 0.27401 | -0.01941 | 0.00274 | 1.50E-12 | 50.06 |
| 20 | rs11043764 | T | A | 0.27404 | 0.01532 | 0.00275 | 2.65E-08 | 30.95 |
| 21 | rs11135450 | G | A | 0.66888 | 0.01607 | 0.00261 | 7.09E-10 | 38.00 |
| 22 | rs11150745 | G | A | 0.31949 | -0.01885 | 0.00263 | 7.10E-13 | 51.52 |
| 23 | rs11205303 | C | T | 0.40913 | 0.02996 | 0.00249 | 2.19E-33 | 144.99 |
| 24 | rs112646560 | T | C | 0.21753 | 0.01910 | 0.00299 | 1.65E-10 | 40.85 |
| 25 | rs11538 | G | A | 0.17213 | 0.01770 | 0.00324 | 4.52E-08 | 29.91 |
| 26 | rs11603783 | C | T | 0.24665 | 0.01789 | 0.00286 | 3.75E-10 | 39.24 |
| 27 | rs11642015 | T | C | 0.40236 | 0.05825 | 0.00249 | 9.53E-121 | 546.40 |
| 28 | rs11742930 | T | C | 0.56643 | 0.01597 | 0.00247 | 1.04E-10 | 41.76 |
| 29 | rs117632017 | A | G | 0.03914 | 0.03879 | 0.00656 | 3.41E-09 | 34.93 |
| 30 | rs11766945 | A | G | 0.20101 | -0.01733 | 0.00307 | 1.69E-08 | 31.83 |
| 31 | rs11779446 | G | A | 0.16052 | -0.02263 | 0.00334 | 1.22E-11 | 45.94 |
| 32 | rs11782341 | G | A | 0.18851 | 0.01866 | 0.00315 | 3.11E-09 | 35.11 |
| 33 | rs1182199 | A | C | 0.30507 | -0.02817 | 0.00266 | 2.81E-26 | 112.50 |
| 34 | rs11856579 | A | G | 0.26819 | -0.01616 | 0.00276 | 4.74E-09 | 34.30 |
| 35 | rs11917068 | C | T | 0.27913 | -0.01584 | 0.00273 | 6.30E-09 | 33.74 |
| 36 | rs12144626 | C | T | 0.58182 | -0.01480 | 0.00249 | 2.71E-09 | 35.38 |
| 37 | rs12213070 | A | G | 0.34721 | -0.01624 | 0.00257 | 2.45E-10 | 40.07 |
| 38 | rs12330631 | T | C | 0.36711 | -0.01511 | 0.00254 | 2.76E-09 | 35.35 |
| 39 | rs12339822 | G | A | 0.54320 | 0.01556 | 0.00246 | 2.72E-10 | 39.87 |
| 40 | rs12367809 | T | C | 0.36828 | 0.02894 | 0.00255 | 5.90E-30 | 129.30 |
| 41 | rs12459368 | G | A | 0.26707 | -0.01774 | 0.00276 | 1.25E-10 | 41.38 |
| 42 | rs12475388 | A | G | 0.48675 | -0.01674 | 0.00246 | 9.78E-12 | 46.38 |
| 43 | rs12578952 | G | A | 0.25170 | -0.01584 | 0.00282 | 1.89E-08 | 31.61 |
| 44 | rs12616638 | G | A | 0.41609 | -0.01533 | 0.00250 | 8.43E-10 | 37.66 |
| 45 | rs12619178 | T | C | 0.40292 | -0.01617 | 0.00249 | 8.68E-11 | 42.10 |
| 46 | rs12679106 | T | G | 0.71134 | -0.02466 | 0.00271 | 9.87E-20 | 82.64 |
| 47 | rs12724928 | C | T | 0.20538 | -0.02087 | 0.00302 | 5.17E-12 | 47.63 |
| 48 | rs12877270 | A | G | 0.43845 | 0.01419 | 0.00248 | 1.09E-08 | 32.67 |
| 49 | rs12901071 | G | A | 0.33474 | 0.01734 | 0.00261 | 3.29E-11 | 44.00 |
| 50 | rs12926311 | C | G | 0.35543 | -0.01596 | 0.00256 | 4.68E-10 | 38.81 |
| 51 | rs12974458 | T | C | 0.54388 | 0.01356 | 0.00246 | 3.60E-08 | 30.35 |
| 52 | rs12992672 | A | G | 0.82864 | 0.04138 | 0.00324 | 2.34E-37 | 163.17 |
| 53 | rs13067187 | A | G | 0.14206 | -0.02259 | 0.00356 | 2.17E-10 | 40.31 |
| 54 | rs13135092 | G | A | 0.08323 | 0.04292 | 0.00445 | 5.62E-22 | 92.87 |
| 55 | rs13191298 | G | A | 0.12061 | 0.02053 | 0.00376 | 4.67E-08 | 29.85 |
| 56 | rs13333747 | C | T | 0.18196 | -0.02453 | 0.00318 | 1.31E-14 | 59.37 |
| 57 | rs13340461 | T | C | 0.27285 | 0.01819 | 0.00275 | 3.73E-11 | 43.75 |
| 58 | rs1336486 | G | T | 0.32880 | 0.01558 | 0.00260 | 2.17E-09 | 35.82 |
| 59 | rs13416004 | T | C | 0.40960 | -0.01645 | 0.00248 | 3.52E-11 | 43.87 |
| 60 | rs1427325 | A | C | 0.69353 | 0.01744 | 0.00265 | 4.78E-11 | 43.27 |
| 61 | rs143384 | G | A | 0.40280 | 0.01899 | 0.00249 | 2.42E-14 | 58.16 |
| 62 | rs1441264 | A | G | 0.59187 | 0.01717 | 0.00254 | 1.39E-11 | 45.68 |
| 63 | rs1446585 | G | A | 0.22801 | -0.01802 | 0.00293 | 7.52E-10 | 37.88 |
| 64 | rs1470050 | T | A | 0.31228 | 0.01459 | 0.00264 | 3.35E-08 | 30.50 |
| 65 | rs147451284 | G | C | 0.05201 | -0.03075 | 0.00552 | 2.58E-08 | 31.00 |
| 66 | rs147730268 | T | G | 0.09053 | -0.04762 | 0.00441 | 3.16E-27 | 116.83 |
| 67 | rs1491905 | C | T | 0.50632 | -0.01342 | 0.00245 | 4.08E-08 | 30.11 |
| 68 | rs1503527 | T | C | 0.47972 | 0.01570 | 0.00245 | 1.39E-10 | 41.18 |
| 69 | rs154001 | T | C | 0.67639 | 0.01502 | 0.00261 | 8.97E-09 | 33.05 |
| 70 | rs1568489 | G | A | 0.57440 | 0.01672 | 0.00248 | 1.57E-11 | 45.45 |
| 71 | rs1627536 | T | A | 0.27634 | 0.01515 | 0.00274 | 3.22E-08 | 30.57 |
| 72 | rs1653892 | C | T | 0.69557 | -0.01668 | 0.00268 | 4.62E-10 | 38.83 |
| 73 | rs16880854 | A | G | 0.16844 | 0.03409 | 0.00327 | 1.88E-25 | 108.73 |
| 74 | rs16934748 | C | T | 0.15159 | 0.01893 | 0.00341 | 2.82E-08 | 30.83 |
| 75 | rs16951304 | C | T | 0.20669 | -0.02696 | 0.00302 | 3.79E-19 | 79.98 |
| 76 | rs16967461 | T | G | 0.09795 | -0.02299 | 0.00412 | 2.37E-08 | 31.16 |
| 77 | rs17024393 | C | T | 0.02580 | 0.06412 | 0.00774 | 1.19E-16 | 68.63 |
| 78 | rs17055384 | T | C | 0.18366 | -0.01752 | 0.00317 | 3.22E-08 | 30.57 |
| 79 | rs17218879 | G | C | 0.33689 | 0.01418 | 0.00260 | 4.72E-08 | 29.83 |
| 80 | rs1724551 | A | G | 0.51213 | -0.01561 | 0.00247 | 2.51E-10 | 40.02 |
| 81 | rs17522122 | T | G | 0.47032 | 0.01644 | 0.00246 | 2.27E-11 | 44.72 |
| 82 | rs17698176 | G | T | 0.22909 | 0.01779 | 0.00294 | 1.35E-09 | 36.73 |
| 83 | rs17770336 | T | C | 0.32382 | 0.02191 | 0.00261 | 4.54E-17 | 70.53 |
| 84 | rs1782508 | G | C | 0.65633 | -0.01586 | 0.00257 | 6.91E-10 | 38.05 |
| 85 | rs1927626 | T | C | 0.93631 | 0.02938 | 0.00502 | 4.68E-09 | 34.32 |
| 86 | rs1964599 | T | C | 0.34371 | -0.02105 | 0.00263 | 1.20E-15 | 64.07 |
| 87 | rs2012027 | T | C | 0.71442 | -0.01919 | 0.00273 | 1.92E-12 | 49.57 |
| 88 | rs2016469 | A | G | 0.37370 | 0.01460 | 0.00255 | 9.95E-09 | 32.85 |
| 89 | rs2102278 | G | A | 0.32208 | 0.01715 | 0.00262 | 6.19E-11 | 42.76 |
| 90 | rs2172131 | C | T | 0.58064 | -0.01608 | 0.00248 | 8.66E-11 | 42.11 |
| 91 | rs2181375 | G | A | 0.59863 | 0.02015 | 0.00249 | 6.25E-16 | 65.36 |
| 92 | rs2216931 | A | C | 0.66168 | 0.01765 | 0.00259 | 9.17E-12 | 46.50 |
| 93 | rs2243928 | G | C | 0.64708 | -0.01668 | 0.00259 | 1.18E-10 | 41.50 |
| 94 | rs2289379 | T | C | 0.39497 | -0.01591 | 0.00251 | 2.31E-10 | 40.19 |
| 95 | rs2306593 | T | C | 0.48852 | -0.01592 | 0.00245 | 8.09E-11 | 42.24 |
| 96 | rs2307111 | C | T | 0.39318 | -0.02600 | 0.00251 | 3.24E-25 | 107.65 |
| 97 | rs2370982 | T | C | 0.21534 | 0.02819 | 0.00300 | 5.33E-21 | 88.42 |
| 98 | rs2371767 | C | G | 0.27311 | 0.01660 | 0.00275 | 1.54E-09 | 36.48 |
| 99 | rs2374947 | G | A | 0.76098 | 0.01595 | 0.00288 | 3.03E-08 | 30.69 |
| 100 | rs241460 | G | A | 0.68187 | -0.02118 | 0.00262 | 6.51E-16 | 65.28 |
| 101 | rs2439823 | G | A | 0.54771 | 0.01895 | 0.00246 | 1.42E-14 | 59.21 |
| 102 | rs245775 | G | A | 0.72883 | 0.02057 | 0.00275 | 7.95E-14 | 55.82 |
| 103 | rs2482704 | T | G | 0.42342 | -0.01426 | 0.00247 | 8.33E-09 | 33.20 |
| 104 | rs2494196 | A | C | 0.28766 | 0.02618 | 0.00270 | 3.24E-22 | 93.96 |
| 105 | rs2500406 | C | A | 0.66889 | -0.01804 | 0.00260 | 3.71E-12 | 48.28 |
| 106 | rs252749 | A | G | 0.24657 | -0.02174 | 0.00283 | 1.59E-14 | 58.98 |
| 107 | rs2678204 | G | T | 0.34181 | 0.02290 | 0.00258 | 7.11E-19 | 78.74 |
| 108 | rs2744965 | T | C | 0.13933 | 0.05080 | 0.00353 | 4.94E-47 | 207.52 |
| 109 | rs274677 | C | T | 0.67142 | -0.01450 | 0.00260 | 2.40E-08 | 31.14 |
| 110 | rs28366156 | C | T | 0.13136 | -0.02899 | 0.00362 | 1.15E-15 | 64.17 |
| 111 | rs2855818 | A | G | 0.23653 | 0.02249 | 0.00289 | 7.82E-15 | 60.39 |
| 112 | rs28636067 | A | G | 0.12992 | 0.02155 | 0.00364 | 3.30E-09 | 35.00 |
| 113 | rs2869950 | T | C | 0.37787 | -0.01634 | 0.00260 | 3.05E-10 | 39.64 |
| 114 | rs287834 | C | T | 0.52248 | -0.01412 | 0.00246 | 9.80E-09 | 32.88 |
| 115 | rs2903738 | T | A | 0.22048 | -0.01732 | 0.00295 | 4.41E-09 | 34.43 |
| 116 | rs2914231 | C | G | 0.23428 | -0.01900 | 0.00291 | 6.47E-11 | 42.67 |
| 117 | rs2954021 | G | A | 0.50596 | 0.01771 | 0.00245 | 4.49E-13 | 52.42 |
| 118 | rs2979655 | G | T | 0.15864 | 0.01881 | 0.00335 | 1.97E-08 | 31.53 |
| 119 | rs33503 | A | G | 0.80601 | -0.01788 | 0.00309 | 7.38E-09 | 33.43 |
| 120 | rs33836 | T | C | 0.46480 | -0.01970 | 0.00248 | 2.00E-15 | 63.07 |
| 121 | rs34045288 | T | C | 0.33590 | 0.02219 | 0.00259 | 1.09E-17 | 73.34 |
| 122 | rs34517439 | A | C | 0.12577 | 0.04203 | 0.00375 | 3.79E-29 | 125.61 |
| 123 | rs35060985 | A | G | 0.31683 | 0.02300 | 0.00263 | 2.47E-18 | 76.28 |
| 124 | rs35344761 | A | C | 0.12176 | -0.02521 | 0.00377 | 2.19E-11 | 44.79 |
| 125 | rs35537311 | T | C | 0.38988 | -0.01381 | 0.00251 | 3.76E-08 | 30.27 |
| 126 | rs3730071 | A | C | 0.03031 | -0.04304 | 0.00713 | 1.58E-09 | 36.44 |
| 127 | rs3803286 | G | A | 0.66638 | -0.01890 | 0.00259 | 2.99E-13 | 53.22 |
| 128 | rs3806251 | T | A | 0.13272 | 0.02354 | 0.00362 | 8.45E-11 | 42.15 |
| 129 | rs3817428 | G | C | 0.26723 | -0.02441 | 0.00276 | 9.60E-19 | 78.15 |
| 130 | rs3853252 | A | G | 0.45557 | 0.01583 | 0.00246 | 1.34E-10 | 41.25 |
| 131 | rs40071 | C | T | 0.17929 | -0.01798 | 0.00319 | 1.80E-08 | 31.70 |
| 132 | rs41271299 | T | C | 0.05171 | 0.04000 | 0.00552 | 4.50E-13 | 52.41 |
| 133 | rs4253755 | A | G | 0.12869 | 0.02099 | 0.00367 | 1.10E-08 | 32.66 |
| 134 | rs4482463 | A | C | 0.92418 | -0.03043 | 0.00463 | 4.85E-11 | 43.24 |
| 135 | rs4549080 | T | C | 0.34101 | 0.01541 | 0.00258 | 2.34E-09 | 35.67 |
| 136 | rs4657796 | C | T | 0.37656 | -0.01474 | 0.00253 | 5.64E-09 | 33.96 |
| 137 | rs4673617 | T | C | 0.27164 | 0.01562 | 0.00276 | 1.49E-08 | 32.07 |
| 138 | rs4709745 | C | T | 0.30544 | 0.01754 | 0.00265 | 3.86E-11 | 43.69 |
| 139 | rs4718964 | T | G | 0.41219 | 0.01850 | 0.00249 | 1.08E-13 | 55.22 |
| 140 | rs4722398 | T | C | 0.13566 | 0.02168 | 0.00356 | 1.12E-09 | 37.10 |
| 141 | rs4757144 | A | G | 0.59305 | 0.01413 | 0.00249 | 1.37E-08 | 32.23 |
| 142 | rs477895 | T | C | 0.83698 | 0.01843 | 0.00330 | 2.46E-08 | 31.09 |
| 143 | rs4804312 | C | T | 0.44384 | -0.01447 | 0.00247 | 4.47E-09 | 34.41 |
| 144 | rs4876611 | G | A | 0.71581 | 0.02291 | 0.00271 | 3.08E-17 | 71.30 |
| 145 | rs4958702 | C | T | 0.57368 | -0.01570 | 0.00247 | 2.08E-10 | 40.40 |
| 146 | rs4981693 | A | G | 0.77273 | 0.02172 | 0.00292 | 1.05E-13 | 55.28 |
| 147 | rs543874 | G | A | 0.20781 | 0.04139 | 0.00301 | 5.47E-43 | 188.98 |
| 148 | rs55637757 | T | C | 0.13344 | -0.02678 | 0.00365 | 2.38E-13 | 53.67 |
| 149 | rs55672437 | C | G | 0.20993 | 0.01875 | 0.00301 | 4.59E-10 | 38.84 |
| 150 | rs55714539 | C | A | 0.34269 | 0.02386 | 0.00259 | 3.54E-20 | 84.67 |
| 151 | rs55726687 | A | G | 0.21114 | 0.02078 | 0.00299 | 3.98E-12 | 48.14 |
| 152 | rs56239516 | C | A | 0.29041 | 0.01492 | 0.00271 | 3.46E-08 | 30.43 |
| 153 | rs56361700 | C | T | 0.05868 | 0.03380 | 0.00523 | 1.02E-10 | 41.79 |
| 154 | rs57636386 | C | T | 0.08342 | -0.03927 | 0.00443 | 7.58E-19 | 78.61 |
| 155 | rs58862095 | T | C | 0.41976 | -0.01809 | 0.00248 | 3.21E-13 | 53.08 |
| 156 | rs59066241 | G | T | 0.11827 | 0.02318 | 0.00380 | 1.10E-09 | 37.14 |
| 157 | rs59815219 | T | C | 0.49604 | 0.01347 | 0.00245 | 3.63E-08 | 30.34 |
| 158 | rs6029180 | G | A | 0.32723 | 0.01503 | 0.00263 | 1.14E-08 | 32.59 |
| 159 | rs60898782 | A | G | 0.20240 | 0.01858 | 0.00306 | 1.28E-09 | 36.84 |
| 160 | rs6135562 | C | T | 0.07951 | -0.02490 | 0.00452 | 3.54E-08 | 30.39 |
| 161 | rs61813293 | T | G | 0.14295 | 0.01995 | 0.00349 | 1.07E-08 | 32.71 |
| 162 | rs61871615 | T | C | 0.09052 | -0.02706 | 0.00445 | 1.21E-09 | 36.96 |
| 163 | rs62037365 | G | C | 0.40213 | 0.03384 | 0.00249 | 6.15E-42 | 184.16 |
| 164 | rs62104483 | A | G | 0.32951 | 0.02356 | 0.00260 | 1.46E-19 | 81.87 |
| 165 | rs62106258 | C | T | 0.04847 | -0.07820 | 0.00569 | 6.61E-43 | 188.60 |
| 166 | rs62246314 | A | G | 0.10128 | 0.02491 | 0.00405 | 7.93E-10 | 37.78 |
| 167 | rs62261725 | G | A | 0.32744 | -0.02040 | 0.00261 | 5.46E-15 | 61.09 |
| 168 | rs62396185 | C | G | 0.25646 | -0.04313 | 0.00280 | 2.20E-53 | 236.66 |
| 169 | rs62407569 | G | A | 0.27621 | 0.01672 | 0.00274 | 1.03E-09 | 37.26 |
| 170 | rs6486057 | A | T | 0.41436 | 0.01370 | 0.00249 | 3.57E-08 | 30.37 |
| 171 | rs6549970 | T | C | 0.54394 | 0.01503 | 0.00245 | 9.08E-10 | 37.51 |
| 172 | rs6567160 | C | T | 0.23406 | 0.04452 | 0.00289 | 1.09E-53 | 238.05 |
| 173 | rs6598540 | G | A | 0.27654 | -0.01870 | 0.00273 | 7.90E-12 | 46.79 |
| 174 | rs66679256 | T | C | 0.44614 | 0.02014 | 0.00246 | 3.05E-16 | 66.78 |
| 175 | rs6696828 | C | G | 0.30611 | 0.01510 | 0.00265 | 1.21E-08 | 32.47 |
| 176 | rs6699744 | T | A | 0.61402 | 0.02069 | 0.00252 | 2.33E-16 | 67.31 |
| 177 | rs6717858 | C | T | 0.40340 | 0.02200 | 0.00250 | 1.18E-18 | 77.74 |
| 178 | rs67536595 | T | C | 0.18548 | -0.01757 | 0.00314 | 2.26E-08 | 31.26 |
| 179 | rs6840236 | C | T | 0.46158 | 0.01584 | 0.00246 | 1.16E-10 | 41.54 |
| 180 | rs6861649 | C | T | 0.60749 | 0.01625 | 0.00251 | 1.00E-10 | 41.82 |
| 181 | rs6948959 | A | G | 0.74393 | -0.01808 | 0.00282 | 1.36E-10 | 41.23 |
| 182 | rs6973656 | G | A | 0.39658 | 0.01581 | 0.00250 | 2.39E-10 | 40.12 |
| 183 | rs704061 | C | T | 0.45270 | 0.01979 | 0.00246 | 8.08E-16 | 64.86 |
| 184 | rs7080838 | T | C | 0.56885 | 0.01536 | 0.00248 | 5.51E-10 | 38.49 |
| 185 | rs7094644 | A | G | 0.67418 | 0.01602 | 0.00266 | 1.63E-09 | 36.37 |
| 186 | rs7124681 | A | C | 0.40806 | 0.02546 | 0.00248 | 1.15E-24 | 105.14 |
| 187 | rs713586 | C | T | 0.48951 | 0.02431 | 0.00244 | 2.43E-23 | 99.09 |
| 188 | rs7167767 | A | G | 0.65102 | 0.01405 | 0.00256 | 4.10E-08 | 30.10 |
| 189 | rs7171864 | A | G | 0.66093 | 0.01925 | 0.00260 | 1.19E-13 | 55.03 |
| 190 | rs7193783 | C | A | 0.64917 | -0.01825 | 0.00258 | 1.38E-12 | 50.21 |
| 191 | rs7238896 | G | A | 0.14072 | 0.02056 | 0.00354 | 6.04E-09 | 33.82 |
| 192 | rs724016 | G | A | 0.44721 | 0.02900 | 0.00246 | 4.13E-32 | 139.15 |
| 193 | rs7259070 | C | T | 0.59901 | 0.01703 | 0.00252 | 1.34E-11 | 45.76 |
| 194 | rs72718281 | T | C | 0.37144 | 0.01951 | 0.00253 | 1.24E-14 | 59.47 |
| 195 | rs72820274 | A | G | 0.41876 | 0.01402 | 0.00248 | 1.62E-08 | 31.91 |
| 196 | rs72917533 | C | T | 0.18627 | -0.01830 | 0.00315 | 6.17E-09 | 33.78 |
| 197 | rs72976986 | A | G | 0.19119 | -0.01923 | 0.00314 | 9.35E-10 | 37.46 |
| 198 | rs72995085 | C | T | 0.17734 | -0.01952 | 0.00321 | 1.23E-09 | 36.93 |
| 199 | rs73041988 | G | T | 0.16616 | -0.02317 | 0.00329 | 1.80E-12 | 49.69 |
| 200 | rs7305790 | C | A | 0.27789 | 0.01601 | 0.00273 | 4.62E-09 | 34.35 |
| 201 | rs7313748 | T | C | 0.28978 | -0.01652 | 0.00269 | 7.98E-10 | 37.77 |
| 202 | rs73142879 | T | C | 0.19217 | -0.02380 | 0.00311 | 2.06E-14 | 58.47 |
| 203 | rs73175572 | G | A | 0.11211 | 0.02177 | 0.00392 | 2.83E-08 | 30.82 |
| 204 | rs73213484 | T | A | 0.13906 | -0.02190 | 0.00354 | 5.81E-10 | 38.38 |
| 205 | rs7442885 | G | C | 0.21010 | -0.02153 | 0.00301 | 8.45E-13 | 51.18 |
| 206 | rs7460093 | A | G | 0.53551 | 0.01389 | 0.00247 | 1.95E-08 | 31.54 |
| 207 | rs750090 | C | T | 0.35615 | -0.01477 | 0.00258 | 1.04E-08 | 32.76 |
| 208 | rs75412871 | T | C | 0.05279 | -0.03149 | 0.00547 | 8.86E-09 | 33.08 |
| 209 | rs75641275 | C | A | 0.14395 | 0.02014 | 0.00348 | 7.41E-09 | 33.43 |
| 210 | rs756717 | A | G | 0.39886 | -0.01477 | 0.00253 | 4.98E-09 | 34.20 |
| 211 | rs7600835 | A | G | 0.34689 | -0.01555 | 0.00261 | 2.44E-09 | 35.59 |
| 212 | rs76040172 | A | G | 0.05444 | -0.03885 | 0.00540 | 6.62E-13 | 51.66 |
| 213 | rs7613261 | T | A | 0.20456 | 0.02311 | 0.00304 | 2.76E-14 | 57.91 |
| 214 | rs76345589 | G | C | 0.06722 | -0.03166 | 0.00490 | 1.05E-10 | 41.73 |
| 215 | rs7649970 | T | C | 0.12087 | 0.03398 | 0.00374 | 1.10E-19 | 82.44 |
| 216 | rs76895963 | G | T | 0.01928 | 0.08744 | 0.00949 | 3.09E-20 | 84.94 |
| 217 | rs7696324 | A | G | 0.31780 | 0.01740 | 0.00263 | 3.40E-11 | 43.93 |
| 218 | rs7697556 | C | T | 0.52566 | -0.01447 | 0.00245 | 3.41E-09 | 34.94 |
| 219 | rs7707394 | A | G | 0.35432 | -0.01838 | 0.00255 | 5.91E-13 | 51.88 |
| 220 | rs7933085 | G | A | 0.50920 | 0.01524 | 0.00246 | 5.69E-10 | 38.43 |
| 221 | rs79535757 | T | A | 0.00820 | -0.07892 | 0.01384 | 1.20E-08 | 32.49 |
| 222 | rs7959830 | T | G | 0.41060 | -0.01768 | 0.00249 | 1.17E-12 | 50.54 |
| 223 | rs7987928 | A | G | 0.79963 | -0.02058 | 0.00306 | 1.63E-11 | 45.37 |
| 224 | rs8087074 | T | G | 0.26112 | 0.01714 | 0.00280 | 8.84E-10 | 37.57 |
| 225 | rs815163 | C | T | 0.56160 | -0.01463 | 0.00246 | 2.89E-09 | 35.26 |
| 226 | rs8178882 | T | C | 0.08749 | 0.02380 | 0.00435 | 4.37E-08 | 29.98 |
| 227 | rs8192675 | C | T | 0.28738 | 0.01653 | 0.00270 | 9.02E-10 | 37.53 |
| 228 | rs846545 | C | T | 0.42032 | -0.01364 | 0.00248 | 3.91E-08 | 30.20 |
| 229 | rs854917 | T | C | 0.73801 | -0.01642 | 0.00279 | 4.22E-09 | 34.52 |
| 230 | rs862320 | T | C | 0.41015 | -0.01853 | 0.00249 | 9.95E-14 | 55.38 |
| 231 | rs879620 | T | C | 0.61528 | 0.02687 | 0.00252 | 1.70E-26 | 113.49 |
| 232 | rs882378 | C | A | 0.30937 | 0.01493 | 0.00265 | 1.83E-08 | 31.67 |
| 233 | rs9320823 | C | T | 0.60190 | 0.02006 | 0.00250 | 1.07E-15 | 64.30 |
| 234 | rs9375702 | T | C | 0.69033 | -0.01732 | 0.00264 | 5.72E-11 | 42.92 |
| 235 | rs952227 | G | A | 0.67316 | -0.01603 | 0.00261 | 7.86E-10 | 37.80 |
| 236 | rs9584855 | G | T | 0.29010 | -0.01601 | 0.00269 | 2.69E-09 | 35.40 |
| 237 | rs962554 | C | T | 0.28437 | -0.01803 | 0.00273 | 3.76E-11 | 43.74 |
| 238 | rs9814633 | A | G | 0.34395 | 0.01608 | 0.00258 | 4.34E-10 | 38.96 |
| 239 | rs9816948 | T | A | 0.26588 | -0.01788 | 0.00278 | 1.26E-10 | 41.37 |
| 240 | rs9861443 | C | A | 0.71366 | 0.01596 | 0.00272 | 4.53E-09 | 34.38 |

SNPs, single nucleotide polymorphisms; EAF, effect allele frequency; SE, standard error.

**Supplementary Table 7: Characteristics of 234 SNPs used as instrumental variables for the effect of Total fat mass on asthma.**

|  | SNP | effect_allele.exposure | other_allele.exposure | EAF.exposure | effect | SE | *P*-value | F-Statistic |
| --- | --- | --- | --- | --- | --- | --- | --- | --- |
| 1 | rs10100245 | A | G | 0.56644 | 0.01915 | 0.00239 | 1.12E-15 | 64.21 |
| 2 | rs10128597 | A | G | 0.27402 | -0.01923 | 0.00267 | 6.27E-13 | 51.76 |
| 3 | rs10141106 | G | A | 0.70214 | 0.01579 | 0.00260 | 1.18E-09 | 37.00 |
| 4 | rs10172196 | A | G | 0.30484 | 0.01864 | 0.00258 | 5.24E-13 | 52.12 |
| 5 | rs10237306 | T | G | 0.38022 | 0.01608 | 0.00244 | 4.36E-11 | 43.45 |
| 6 | rs10269774 | A | G | 0.32354 | 0.01820 | 0.00253 | 6.89E-13 | 51.58 |
| 7 | rs10423928 | A | T | 0.19349 | -0.03071 | 0.00299 | 9.55E-25 | 105.50 |
| 8 | rs10490530 | G | A | 0.13916 | 0.01904 | 0.00343 | 2.82E-08 | 30.83 |
| 9 | rs10499014 | G | C | 0.26787 | -0.01598 | 0.00269 | 3.01E-09 | 35.18 |
| 10 | rs10507483 | C | T | 0.16534 | 0.01735 | 0.00318 | 4.92E-08 | 29.75 |
| 11 | rs10748028 | A | C | 0.44044 | -0.01508 | 0.00242 | 4.91E-10 | 38.71 |
| 12 | rs10756714 | G | A | 0.44479 | -0.02269 | 0.00239 | 2.08E-21 | 90.28 |
| 13 | rs10756798 | T | C | 0.64503 | -0.01782 | 0.00248 | 6.49E-13 | 51.70 |
| 14 | rs10760724 | C | T | 0.47394 | 0.01484 | 0.00237 | 3.90E-10 | 39.16 |
| 15 | rs10787738 | T | C | 0.25330 | 0.01986 | 0.00276 | 6.54E-13 | 51.68 |
| 16 | rs10820852 | A | C | 0.27434 | -0.01603 | 0.00266 | 1.68E-09 | 36.31 |
| 17 | rs10827289 | T | C | 0.58303 | -0.01393 | 0.00241 | 7.43E-09 | 33.42 |
| 18 | rs10915840 | A | G | 0.27215 | -0.01551 | 0.00268 | 6.86E-09 | 33.57 |
| 19 | rs10938397 | G | A | 0.43408 | 0.02666 | 0.00240 | 9.20E-29 | 123.85 |
| 20 | rs10947793 | G | A | 0.37360 | -0.01604 | 0.00246 | 7.36E-11 | 42.42 |
| 21 | rs10954772 | C | T | 0.68571 | -0.01748 | 0.00257 | 9.95E-12 | 46.34 |
| 22 | rs10999460 | T | C | 0.26595 | 0.01573 | 0.00269 | 5.15E-09 | 34.13 |
| 23 | rs11030119 | A | G | 0.30881 | 0.03105 | 0.00257 | 1.05E-33 | 146.46 |
| 24 | rs11150745 | G | A | 0.31949 | -0.01934 | 0.00254 | 2.97E-14 | 57.76 |
| 25 | rs111640872 | C | G | 0.33108 | 0.02285 | 0.00252 | 1.43E-19 | 81.91 |
| 26 | rs11205303 | C | T | 0.40913 | 0.02222 | 0.00241 | 3.07E-20 | 84.95 |
| 27 | rs11205617 | A | G | 0.31713 | 0.02102 | 0.00254 | 1.26E-16 | 68.52 |
| 28 | rs113866544 | C | T | 0.06768 | 0.03614 | 0.00472 | 1.95E-14 | 58.59 |
| 29 | rs11474838 | G | T | 0.43273 | 0.01698 | 0.00244 | 3.32E-12 | 48.50 |
| 30 | rs11538 | G | A | 0.17213 | 0.01838 | 0.00314 | 4.62E-09 | 34.34 |
| 31 | rs11580836 | T | C | 0.54353 | 0.01366 | 0.00238 | 9.80E-09 | 32.88 |
| 32 | rs11603783 | C | T | 0.24665 | 0.01672 | 0.00277 | 1.51E-09 | 36.52 |
| 33 | rs11642015 | T | C | 0.40236 | 0.06090 | 0.00241 | 9.53E-121 | 636.20 |
| 34 | rs11656758 | G | A | 0.33962 | 0.01613 | 0.00250 | 1.20E-10 | 41.48 |
| 35 | rs11695700 | T | G | 0.41767 | 0.01328 | 0.00241 | 3.64E-08 | 30.33 |
| 36 | rs11725731 | T | C | 0.26633 | 0.01497 | 0.00268 | 2.43E-08 | 31.12 |
| 37 | rs11764337 | T | C | 0.18325 | -0.01780 | 0.00307 | 6.89E-09 | 33.57 |
| 38 | rs11782341 | G | A | 0.18851 | 0.01826 | 0.00305 | 2.17E-09 | 35.81 |
| 39 | rs11786089 | G | A | 0.45685 | 0.01404 | 0.00238 | 3.87E-09 | 34.69 |
| 40 | rs1182199 | A | C | 0.30507 | -0.02331 | 0.00257 | 1.36E-19 | 82.02 |
| 41 | rs11824092 | C | T | 0.63745 | 0.01396 | 0.00248 | 1.78E-08 | 31.72 |
| 42 | rs11857221 | A | C | 0.39689 | 0.01322 | 0.00242 | 4.79E-08 | 29.80 |
| 43 | rs11958027 | G | A | 0.57644 | 0.01546 | 0.00240 | 1.24E-10 | 41.41 |
| 44 | rs12031634 | A | G | 0.29869 | -0.01601 | 0.00261 | 8.03E-10 | 37.75 |
| 45 | rs12128526 | A | G | 0.45998 | 0.01330 | 0.00238 | 2.32E-08 | 31.21 |
| 46 | rs12140153 | T | G | 0.09676 | -0.03342 | 0.00411 | 4.27E-16 | 66.11 |
| 47 | rs12144626 | C | T | 0.58182 | -0.01504 | 0.00241 | 4.40E-10 | 38.93 |
| 48 | rs12254441 | T | C | 0.37284 | -0.01551 | 0.00254 | 9.47E-10 | 37.43 |
| 49 | rs1229984 | C | T | 0.97755 | 0.04542 | 0.00800 | 1.40E-08 | 32.19 |
| 50 | rs12330631 | T | C | 0.36711 | -0.01464 | 0.00246 | 2.76E-09 | 35.35 |
| 51 | rs12339822 | G | A | 0.54320 | 0.01622 | 0.00239 | 1.13E-11 | 46.09 |
| 52 | rs12367809 | T | C | 0.36828 | 0.02795 | 0.00247 | 9.36E-30 | 128.38 |
| 53 | rs12459368 | G | A | 0.26707 | -0.01751 | 0.00267 | 5.70E-11 | 42.92 |
| 54 | rs12475388 | A | G | 0.48675 | -0.01503 | 0.00238 | 2.80E-10 | 39.81 |
| 55 | rs12477385 | T | G | 0.22730 | -0.01586 | 0.00284 | 2.27E-08 | 31.25 |
| 56 | rs12578952 | G | A | 0.25170 | -0.01739 | 0.00273 | 1.90E-10 | 40.57 |
| 57 | rs12610925 | G | A | 0.35177 | 0.01831 | 0.00249 | 1.78E-13 | 54.23 |
| 58 | rs12616638 | G | A | 0.41609 | -0.01431 | 0.00242 | 3.41E-09 | 34.94 |
| 59 | rs12619178 | T | C | 0.40292 | -0.01673 | 0.00242 | 4.30E-12 | 47.99 |
| 60 | rs12877270 | A | G | 0.43845 | 0.01415 | 0.00241 | 4.04E-09 | 34.61 |
| 61 | rs12887636 | G | T | 0.34740 | -0.01447 | 0.00250 | 7.59E-09 | 33.38 |
| 62 | rs12890931 | G | T | 0.36346 | 0.01426 | 0.00248 | 8.76E-09 | 33.10 |
| 63 | rs12987931 | T | C | 0.61490 | 0.01388 | 0.00245 | 1.42E-08 | 32.16 |
| 64 | rs12992672 | A | G | 0.82864 | 0.04325 | 0.00314 | 3.50E-43 | 189.86 |
| 65 | rs13062093 | G | T | 0.36644 | 0.01682 | 0.00246 | 7.67E-12 | 46.85 |
| 66 | rs13067187 | A | G | 0.14206 | -0.02049 | 0.00345 | 2.79E-09 | 35.33 |
| 67 | rs13135092 | G | A | 0.08323 | 0.04173 | 0.00432 | 4.13E-22 | 93.48 |
| 68 | rs13174863 | G | A | 0.14821 | 0.02441 | 0.00335 | 3.48E-13 | 52.92 |
| 69 | rs13333747 | C | T | 0.18196 | -0.02368 | 0.00309 | 1.65E-14 | 58.92 |
| 70 | rs1335055 | A | G | 0.65578 | -0.01601 | 0.00252 | 2.07E-10 | 40.40 |
| 71 | rs13427822 | G | A | 0.27205 | -0.01567 | 0.00269 | 5.79E-09 | 33.91 |
| 72 | rs1383723 | T | A | 0.78249 | -0.01732 | 0.00288 | 1.87E-09 | 36.11 |
| 73 | rs1441264 | A | G | 0.59187 | 0.01749 | 0.00246 | 1.21E-12 | 50.48 |
| 74 | rs1446585 | G | A | 0.22801 | -0.01744 | 0.00284 | 7.92E-10 | 37.78 |
| 75 | rs1470749 | T | G | 0.51060 | -0.01385 | 0.00237 | 5.24E-09 | 34.10 |
| 76 | rs147730268 | T | G | 0.09053 | -0.04446 | 0.00427 | 2.15E-25 | 108.46 |
| 77 | rs1491905 | C | T | 0.50632 | -0.01355 | 0.00237 | 1.07E-08 | 32.71 |
| 78 | rs1503527 | T | C | 0.47972 | 0.01516 | 0.00237 | 1.61E-10 | 40.90 |
| 79 | rs1568489 | G | A | 0.57440 | 0.01628 | 0.00240 | 1.25E-11 | 45.89 |
| 80 | rs1653892 | C | T | 0.69557 | -0.01695 | 0.00259 | 6.30E-11 | 42.73 |
| 81 | rs17014332 | C | T | 0.21061 | 0.01705 | 0.00290 | 4.31E-09 | 34.48 |
| 82 | rs17024393 | C | T | 0.02580 | 0.06436 | 0.00750 | 9.12E-18 | 73.70 |
| 83 | rs17218879 | G | C | 0.33689 | 0.01417 | 0.00252 | 1.77E-08 | 31.74 |
| 84 | rs1724551 | A | G | 0.51213 | -0.01601 | 0.00239 | 2.16E-11 | 44.82 |
| 85 | rs17770336 | T | C | 0.32382 | 0.02213 | 0.00253 | 2.08E-18 | 76.62 |
| 86 | rs1782508 | G | C | 0.65633 | -0.01521 | 0.00249 | 1.01E-09 | 37.30 |
| 87 | rs1840126 | C | A | 0.56206 | -0.01311 | 0.00239 | 4.04E-08 | 30.13 |
| 88 | rs1879529 | T | G | 0.26510 | -0.01639 | 0.00270 | 1.35E-09 | 36.74 |
| 89 | rs1881505 | C | T | 0.94210 | -0.02813 | 0.00512 | 3.98E-08 | 30.16 |
| 90 | rs1927626 | T | C | 0.93631 | 0.02710 | 0.00486 | 2.47E-08 | 31.08 |
| 91 | rs1928496 | T | C | 0.74239 | 0.01656 | 0.00271 | 9.41E-10 | 37.45 |
| 92 | rs1964599 | T | C | 0.34371 | -0.01860 | 0.00255 | 2.91E-13 | 53.27 |
| 93 | rs2016469 | A | G | 0.37370 | 0.01433 | 0.00247 | 6.42E-09 | 33.70 |
| 94 | rs2102278 | G | A | 0.32208 | 0.01613 | 0.00254 | 2.21E-10 | 40.27 |
| 95 | rs2172131 | C | T | 0.58064 | -0.01671 | 0.00240 | 3.46E-12 | 48.41 |
| 96 | rs2181375 | G | A | 0.59863 | 0.01904 | 0.00242 | 3.23E-15 | 62.13 |
| 97 | rs2192527 | G | A | 0.46495 | 0.01873 | 0.00238 | 3.59E-15 | 61.92 |
| 98 | rs2239647 | C | A | 0.55179 | -0.01817 | 0.00238 | 2.49E-14 | 58.11 |
| 99 | rs2289379 | T | C | 0.39497 | -0.01669 | 0.00243 | 6.76E-12 | 47.10 |
| 100 | rs2292238 | C | A | 0.40867 | -0.01339 | 0.00242 | 2.98E-08 | 30.72 |
| 101 | rs2307111 | C | T | 0.39318 | -0.02694 | 0.00243 | 1.34E-28 | 123.09 |
| 102 | rs2370982 | T | C | 0.21534 | 0.02731 | 0.00290 | 5.41E-21 | 88.39 |
| 103 | rs2374947 | G | A | 0.76098 | 0.01603 | 0.00279 | 9.21E-09 | 33.00 |
| 104 | rs2425024 | C | A | 0.32374 | 0.01615 | 0.00253 | 1.73E-10 | 40.75 |
| 105 | rs2439823 | G | A | 0.54771 | 0.01962 | 0.00239 | 2.03E-16 | 67.58 |
| 106 | rs245775 | G | A | 0.72883 | 0.01959 | 0.00267 | 2.08E-13 | 53.93 |
| 107 | rs2479958 | G | A | 0.51515 | -0.01664 | 0.00240 | 3.77E-12 | 48.24 |
| 108 | rs2482398 | A | C | 0.52250 | -0.01452 | 0.00239 | 1.14E-09 | 37.07 |
| 109 | rs2494196 | A | C | 0.28766 | 0.02202 | 0.00262 | 4.00E-17 | 70.78 |
| 110 | rs2499468 | A | C | 0.65274 | 0.01471 | 0.00249 | 3.58E-09 | 34.84 |
| 111 | rs252749 | A | G | 0.24657 | -0.02007 | 0.00274 | 2.58E-13 | 53.51 |
| 112 | rs2606228 | C | A | 0.64277 | -0.01406 | 0.00250 | 1.93E-08 | 31.57 |
| 113 | rs2678204 | G | T | 0.34181 | 0.02387 | 0.00250 | 1.35E-21 | 91.13 |
| 114 | rs28366156 | C | T | 0.13136 | -0.02812 | 0.00351 | 1.07E-15 | 64.30 |
| 115 | rs28457808 | G | C | 0.16319 | -0.01868 | 0.00321 | 6.09E-09 | 33.81 |
| 116 | rs2861685 | C | T | 0.41137 | -0.01653 | 0.00240 | 6.31E-12 | 47.23 |
| 117 | rs28636067 | A | G | 0.12992 | 0.01950 | 0.00353 | 3.32E-08 | 30.51 |
| 118 | rs2869950 | T | C | 0.37787 | -0.01480 | 0.00251 | 3.98E-09 | 34.63 |
| 119 | rs2917705 | A | G | 0.13939 | -0.02009 | 0.00343 | 4.56E-09 | 34.37 |
| 120 | rs2954021 | G | A | 0.50596 | 0.01646 | 0.00237 | 3.79E-12 | 48.24 |
| 121 | rs34045288 | T | C | 0.33590 | 0.02233 | 0.00251 | 5.85E-19 | 79.13 |
| 122 | rs34292685 | T | C | 0.16209 | -0.01778 | 0.00321 | 3.05E-08 | 30.68 |
| 123 | rs34517439 | A | C | 0.12577 | 0.04039 | 0.00363 | 1.08E-28 | 123.53 |
| 124 | rs34769775 | T | C | 0.29786 | -0.01847 | 0.00259 | 9.46E-13 | 50.96 |
| 125 | rs34966008 | T | C | 0.40815 | -0.01759 | 0.00241 | 2.94E-13 | 53.25 |
| 126 | rs35589149 | C | G | 0.13248 | 0.02175 | 0.00350 | 5.20E-10 | 38.60 |
| 127 | rs35851183 | G | A | 0.35778 | 0.01605 | 0.00247 | 8.87E-11 | 42.06 |
| 128 | rs3730071 | A | C | 0.03031 | -0.04069 | 0.00691 | 3.88E-09 | 34.68 |
| 129 | rs3737992 | A | G | 0.16848 | -0.01914 | 0.00317 | 1.51E-09 | 36.52 |
| 130 | rs3766823 | A | G | 0.17164 | 0.02180 | 0.00314 | 3.99E-12 | 48.13 |
| 131 | rs3784699 | C | T | 0.60302 | 0.02276 | 0.00242 | 6.22E-21 | 88.11 |
| 132 | rs3803286 | G | A | 0.66638 | -0.01946 | 0.00251 | 8.97E-15 | 60.12 |
| 133 | rs3826408 | T | C | 0.45721 | 0.01330 | 0.00238 | 2.20E-08 | 31.31 |
| 134 | rs40071 | C | T | 0.17929 | -0.02062 | 0.00310 | 2.70E-11 | 44.39 |
| 135 | rs4471907 | A | G | 0.52876 | 0.01340 | 0.00238 | 1.68E-08 | 31.84 |
| 136 | rs4482463 | A | C | 0.92418 | -0.03096 | 0.00448 | 5.06E-12 | 47.67 |
| 137 | rs4502882 | T | C | 0.65689 | -0.01616 | 0.00249 | 9.19E-11 | 41.99 |
| 138 | rs4503172 | T | C | 0.60782 | -0.01395 | 0.00243 | 9.26E-09 | 32.99 |
| 139 | rs4549080 | T | C | 0.34101 | 0.01602 | 0.00250 | 1.47E-10 | 41.07 |
| 140 | rs4657796 | C | T | 0.37656 | -0.01494 | 0.00245 | 1.10E-09 | 37.14 |
| 141 | rs4673617 | T | C | 0.27164 | 0.01637 | 0.00267 | 9.11E-10 | 37.51 |
| 142 | rs4718964 | T | G | 0.41219 | 0.01729 | 0.00241 | 7.69E-13 | 51.36 |
| 143 | rs4794222 | G | A | 0.74429 | -0.01497 | 0.00272 | 3.87E-08 | 30.21 |
| 144 | rs4864201 | C | T | 0.65330 | -0.01525 | 0.00249 | 8.69E-10 | 37.60 |
| 145 | rs4876611 | G | A | 0.71581 | 0.02175 | 0.00263 | 1.33E-16 | 68.42 |
| 146 | rs4958702 | C | T | 0.57368 | -0.01561 | 0.00239 | 7.01E-11 | 42.52 |
| 147 | rs543874 | G | A | 0.20781 | 0.04237 | 0.00292 | 9.06E-48 | 210.89 |
| 148 | rs55637757 | T | C | 0.13344 | -0.02359 | 0.00354 | 2.75E-11 | 44.35 |
| 149 | rs55714539 | C | A | 0.34269 | 0.02228 | 0.00251 | 7.36E-19 | 78.67 |
| 150 | rs55726687 | A | G | 0.21114 | 0.02215 | 0.00290 | 2.28E-14 | 58.28 |
| 151 | rs56361700 | C | T | 0.05868 | 0.02788 | 0.00507 | 3.72E-08 | 30.29 |
| 152 | rs57636386 | C | T | 0.08342 | -0.03980 | 0.00429 | 1.84E-20 | 85.97 |
| 153 | rs58862095 | T | C | 0.41976 | -0.01807 | 0.00241 | 6.09E-14 | 56.35 |
| 154 | rs59066241 | G | T | 0.11827 | 0.02261 | 0.00369 | 8.58E-10 | 37.62 |
| 155 | rs59104534 | T | C | 0.30111 | 0.01495 | 0.00259 | 8.18E-09 | 33.23 |
| 156 | rs59227842 | G | A | 0.31243 | 0.02395 | 0.00258 | 1.75E-20 | 86.07 |
| 157 | rs6029180 | G | A | 0.32723 | 0.01409 | 0.00255 | 3.35E-08 | 30.49 |
| 158 | rs60898782 | A | G | 0.20240 | 0.01750 | 0.00297 | 3.70E-09 | 34.78 |
| 159 | rs61649432 | T | C | 0.30959 | 0.01427 | 0.00256 | 2.58E-08 | 31.00 |
| 160 | rs61813293 | T | G | 0.14295 | 0.02023 | 0.00338 | 2.16E-09 | 35.83 |
| 161 | rs61871615 | T | C | 0.09052 | -0.02602 | 0.00431 | 1.62E-09 | 36.38 |
| 162 | rs61903695 | G | A | 0.25600 | 0.01556 | 0.00271 | 9.93E-09 | 32.86 |
| 163 | rs62037365 | G | C | 0.40213 | 0.03122 | 0.00242 | 3.62E-38 | 166.89 |
| 164 | rs62106258 | C | T | 0.04847 | -0.08007 | 0.00552 | 1.04E-47 | 210.62 |
| 165 | rs62178054 | C | T | 0.37189 | -0.01742 | 0.00246 | 1.57E-12 | 49.96 |
| 166 | rs62246314 | A | G | 0.10128 | 0.02457 | 0.00393 | 3.93E-10 | 39.15 |
| 167 | rs62261725 | G | A | 0.32744 | -0.02058 | 0.00253 | 4.01E-16 | 66.24 |
| 168 | rs62343137 | C | G | 0.37069 | 0.01798 | 0.00245 | 2.37E-13 | 53.67 |
| 169 | rs62407569 | G | A | 0.27621 | 0.01703 | 0.00265 | 1.40E-10 | 41.17 |
| 170 | rs62499696 | C | G | 0.29293 | 0.01456 | 0.00264 | 3.37E-08 | 30.48 |
| 171 | rs6561937 | A | T | 0.75176 | -0.01501 | 0.00275 | 4.82E-08 | 29.79 |
| 172 | rs6567160 | C | T | 0.23406 | 0.04592 | 0.00280 | 1.34E-60 | 269.77 |
| 173 | rs6598540 | G | A | 0.27654 | -0.01831 | 0.00265 | 4.73E-12 | 47.80 |
| 174 | rs6601527 | A | C | 0.58782 | -0.01521 | 0.00241 | 2.91E-10 | 39.74 |
| 175 | rs6699744 | T | A | 0.61402 | 0.02192 | 0.00244 | 2.97E-19 | 80.47 |
| 176 | rs6717858 | C | T | 0.40340 | 0.01921 | 0.00242 | 1.97E-15 | 63.10 |
| 177 | rs67536595 | T | C | 0.18548 | -0.01733 | 0.00305 | 1.28E-08 | 32.37 |
| 178 | rs6840236 | C | T | 0.46158 | 0.01417 | 0.00238 | 2.73E-09 | 35.37 |
| 179 | rs6861649 | C | T | 0.60749 | 0.01616 | 0.00244 | 3.24E-11 | 44.03 |
| 180 | rs6875585 | C | A | 0.67000 | 0.01548 | 0.00253 | 8.89E-10 | 37.56 |
| 181 | rs6898357 | G | A | 0.68048 | -0.01399 | 0.00254 | 3.53E-08 | 30.39 |
| 182 | rs6973656 | G | A | 0.39658 | 0.01511 | 0.00242 | 4.19E-10 | 39.02 |
| 183 | rs704061 | C | T | 0.45270 | 0.01853 | 0.00238 | 7.06E-15 | 60.59 |
| 184 | rs7094644 | A | G | 0.67418 | 0.01614 | 0.00257 | 3.66E-10 | 39.29 |
| 185 | rs7124681 | A | C | 0.40806 | 0.02457 | 0.00241 | 1.84E-24 | 104.21 |
| 186 | rs713586 | C | T | 0.48951 | 0.02609 | 0.00237 | 2.99E-28 | 121.51 |
| 187 | rs7171864 | A | G | 0.66093 | 0.01791 | 0.00251 | 1.05E-12 | 50.74 |
| 188 | rs7193783 | C | A | 0.64917 | -0.01721 | 0.00250 | 5.26E-12 | 47.59 |
| 189 | rs7238896 | G | A | 0.14072 | 0.02116 | 0.00343 | 6.57E-10 | 38.15 |
| 190 | rs724016 | G | A | 0.44721 | 0.02360 | 0.00238 | 3.99E-23 | 98.11 |
| 191 | rs7259070 | C | T | 0.59901 | 0.01764 | 0.00244 | 4.77E-13 | 52.30 |
| 192 | rs72820274 | A | G | 0.41876 | 0.01386 | 0.00241 | 8.27E-09 | 33.21 |
| 193 | rs72892910 | T | G | 0.17044 | 0.03536 | 0.00316 | 4.68E-29 | 125.19 |
| 194 | rs72906282 | G | T | 0.01526 | 0.05474 | 0.00969 | 1.62E-08 | 31.91 |
| 195 | rs72917533 | C | T | 0.18627 | -0.01768 | 0.00305 | 6.94E-09 | 33.55 |
| 196 | rs72976986 | A | G | 0.19119 | -0.01970 | 0.00305 | 1.00E-10 | 41.82 |
| 197 | rs73041988 | G | T | 0.16616 | -0.02342 | 0.00319 | 1.96E-13 | 54.05 |
| 198 | rs7313748 | T | C | 0.28978 | -0.01602 | 0.00261 | 7.81E-10 | 37.81 |
| 199 | rs73142879 | T | C | 0.19217 | -0.02391 | 0.00302 | 2.27E-15 | 62.82 |
| 200 | rs73213484 | T | A | 0.13906 | -0.02219 | 0.00343 | 9.34E-11 | 41.96 |
| 201 | rs7442885 | G | C | 0.21010 | -0.02307 | 0.00292 | 2.51E-15 | 62.62 |
| 202 | rs7460093 | A | G | 0.53551 | 0.01365 | 0.00240 | 1.23E-08 | 32.45 |
| 203 | rs750090 | C | T | 0.35615 | -0.01490 | 0.00250 | 2.52E-09 | 35.53 |
| 204 | rs75412871 | T | C | 0.05279 | -0.02935 | 0.00531 | 3.16E-08 | 30.61 |
| 205 | rs75557510 | G | A | 0.06150 | -0.03343 | 0.00503 | 3.14E-11 | 44.09 |
| 206 | rs75641275 | C | A | 0.14395 | 0.02075 | 0.00338 | 7.92E-10 | 37.78 |
| 207 | rs759502 | A | G | 0.58782 | -0.01313 | 0.00241 | 4.79E-08 | 29.80 |
| 208 | rs76040172 | A | G | 0.05444 | -0.03973 | 0.00524 | 3.30E-14 | 57.55 |
| 209 | rs7613261 | T | A | 0.20456 | 0.02321 | 0.00294 | 3.05E-15 | 62.24 |
| 210 | rs7649970 | T | C | 0.12087 | 0.02996 | 0.00363 | 1.46E-16 | 68.23 |
| 211 | rs765875 | T | C | 0.48839 | -0.01392 | 0.00237 | 4.17E-09 | 34.55 |
| 212 | rs76895963 | G | T | 0.01928 | 0.07805 | 0.00919 | 2.07E-17 | 72.09 |
| 213 | rs7707394 | A | G | 0.35432 | -0.01881 | 0.00247 | 2.81E-14 | 57.87 |
| 214 | rs7845090 | A | G | 0.71112 | -0.02429 | 0.00263 | 2.40E-20 | 85.44 |
| 215 | rs79113395 | A | G | 0.26545 | -0.01693 | 0.00269 | 3.34E-10 | 39.47 |
| 216 | rs7933085 | G | A | 0.50920 | 0.01551 | 0.00238 | 7.36E-11 | 42.42 |
| 217 | rs7987928 | A | G | 0.79963 | -0.01975 | 0.00296 | 2.57E-11 | 44.48 |
| 218 | rs801738 | G | C | 0.35841 | -0.01959 | 0.00247 | 2.25E-15 | 62.83 |
| 219 | rs8087074 | T | G | 0.26112 | 0.01628 | 0.00271 | 1.89E-09 | 36.08 |
| 220 | rs8118253 | A | T | 0.13190 | 0.01929 | 0.00351 | 3.71E-08 | 30.30 |
| 221 | rs815163 | C | T | 0.56160 | -0.01532 | 0.00239 | 1.37E-10 | 41.21 |
| 222 | rs8192675 | C | T | 0.28738 | 0.01633 | 0.00261 | 4.19E-10 | 39.02 |
| 223 | rs854917 | T | C | 0.73801 | -0.01633 | 0.00271 | 1.66E-09 | 36.34 |
| 224 | rs862320 | T | C | 0.41015 | -0.01813 | 0.00241 | 5.74E-14 | 56.46 |
| 225 | rs869400 | G | T | 0.81583 | 0.02056 | 0.00306 | 1.95E-11 | 45.02 |
| 226 | rs879620 | T | C | 0.61528 | 0.02643 | 0.00244 | 3.01E-27 | 116.93 |
| 227 | rs9320823 | C | T | 0.60190 | 0.02033 | 0.00242 | 5.06E-17 | 70.32 |
| 228 | rs9375702 | T | C | 0.69033 | -0.01719 | 0.00256 | 1.96E-11 | 45.01 |
| 229 | rs9569933 | T | C | 0.18945 | -0.01756 | 0.00303 | 6.99E-09 | 33.54 |
| 230 | rs9584855 | G | T | 0.29010 | -0.01580 | 0.00261 | 1.37E-09 | 36.72 |
| 231 | rs9610311 | C | T | 0.30652 | 0.01444 | 0.00264 | 4.44E-08 | 29.95 |
| 232 | rs9843007 | T | A | 0.37540 | -0.01434 | 0.00245 | 4.52E-09 | 34.39 |
| 233 | rs9861443 | C | A | 0.71366 | 0.01596 | 0.00264 | 1.44E-09 | 36.62 |
| 234 | rs9968060 | T | C | 0.64661 | 0.01506 | 0.00252 | 2.34E-09 | 35.67 |

SNPs, single nucleotide polymorphisms; EAF, effect allele frequency; SE, standard error.

**Supplementary Table 8: Characteristics of 14 SNPs used as instrumental variables for the effect of asthma on Left arm fat mass.**

|  | SNP | effect_allele.exposure | other_allele.exposure | EAF.exposure | effect | SE | *P*-value | F-Statistic |
| --- | --- | --- | --- | --- | --- | --- | --- | --- |
| 1 | rs11137222 | T | C | 0.0357 | 0.1724 | 0.0314 | 4.01E-08 | 30.14 |
| 2 | rs11667612 | T | G | 0.0528 | 0.1471 | 0.0259 | 1.43E-08 | 32.26 |
| 3 | rs118013485 | A | G | 0.0984 | -0.1108 | 0.0197 | 1.92E-08 | 31.63 |
| 4 | rs17293632 | T | C | 0.2622 | 0.0990 | 0.0131 | 4.66E-14 | 57.11 |
| 5 | rs1837253 | C | T | 0.7550 | 0.1345 | 0.0137 | 1.23E-22 | 96.38 |
| 6 | rs186856025 | T | C | 0.0596 | -0.1571 | 0.0248 | 2.32E-10 | 40.13 |
| 7 | rs2325259 | C | T | 0.2511 | -0.0758 | 0.0134 | 1.69E-08 | 32.00 |
| 8 | rs35656734 | T | C | 0.4264 | -0.1122 | 0.0131 | 1.00E-17 | 73.36 |
| 9 | rs60227565 | A | G | 0.1539 | -0.1189 | 0.0161 | 1.74E-13 | 54.54 |
| 10 | rs62192043 | A | G | 0.2335 | -0.1015 | 0.0140 | 3.51E-13 | 52.56 |
| 11 | rs6894249 | G | A | 0.4652 | 0.0932 | 0.0116 | 9.29E-16 | 64.55 |
| 12 | rs7035413 | G | A | 0.1886 | 0.1302 | 0.0148 | 1.69E-18 | 77.39 |
| 13 | rs74630264 | A | G | 0.0816 | -0.1501 | 0.0214 | 2.49E-12 | 49.20 |
| 14 | rs8074437 | G | T | 0.4464 | 0.0981 | 0.0117 | 4.17E-17 | 70.30 |

SNPs, single nucleotide polymorphisms; EAF, effect allele frequency; SE, standard error.

**Supplementary Table 9: Characteristics of 14 SNPs used as instrumental variables for the effect of asthma on Left leg fat mass.**

|  | SNP | effect_allele.exposure | other_allele.exposure | EAF.exposure | effect | SE | *P*-value | F-Statistic |
| --- | --- | --- | --- | --- | --- | --- | --- | --- |
| 1 | rs11137222 | T | C | 0.0357 | 0.1724 | 0.0314 | 4.01E-08 | 30.14 |
| 2 | rs11667612 | T | G | 0.0528 | 0.1471 | 0.0259 | 1.43E-08 | 32.26 |
| 3 | rs118013485 | A | G | 0.0984 | -0.1108 | 0.0197 | 1.92E-08 | 31.63 |
| 4 | rs17293632 | T | C | 0.2622 | 0.0990 | 0.0131 | 4.66E-14 | 57.11 |
| 5 | rs1837253 | C | T | 0.7550 | 0.1345 | 0.0137 | 1.23E-22 | 96.38 |
| 6 | rs186856025 | T | C | 0.0596 | -0.1571 | 0.0248 | 2.32E-10 | 40.13 |
| 7 | rs2325259 | C | T | 0.2511 | -0.0758 | 0.0134 | 1.69E-08 | 32.00 |
| 8 | rs35656734 | T | C | 0.4264 | -0.1122 | 0.0131 | 1.00E-17 | 73.36 |
| 9 | rs60227565 | A | G | 0.1539 | -0.1189 | 0.0161 | 1.74E-13 | 54.54 |
| 10 | rs62192043 | A | G | 0.2335 | -0.1015 | 0.0140 | 3.51E-13 | 52.56 |
| 11 | rs6894249 | G | A | 0.4652 | 0.0932 | 0.0116 | 9.29E-16 | 64.55 |
| 12 | rs7035413 | G | A | 0.1886 | 0.1302 | 0.0148 | 1.69E-18 | 77.39 |
| 13 | rs74630264 | A | G | 0.0816 | -0.1501 | 0.0214 | 2.49E-12 | 49.20 |
| 14 | rs8074437 | G | T | 0.4464 | 0.0981 | 0.0117 | 4.17E-17 | 70.30 |

SNPs, single nucleotide polymorphisms; EAF, effect allele frequency; SE, standard error.

**Supplementary Table 10: Characteristics of 14 SNPs used as instrumental variables for the effect of asthma on Right arm fat mass.**

|  | SNP | effect_allele.exposure | other_allele.exposure | EAF.exposure | effect | SE | *P*-value | F-Statistic |
| --- | --- | --- | --- | --- | --- | --- | --- | --- |
| 1 | rs11137222 | T | C | 0.0357 | 0.1724 | 0.0314 | 4.01E-08 | 30.14 |
| 2 | rs11667612 | T | G | 0.0528 | 0.1471 | 0.0259 | 1.43E-08 | 32.26 |
| 3 | rs118013485 | A | G | 0.0984 | -0.1108 | 0.0197 | 1.92E-08 | 31.63 |
| 4 | rs17293632 | T | C | 0.2622 | 0.0990 | 0.0131 | 4.66E-14 | 57.11 |
| 5 | rs1837253 | C | T | 0.7550 | 0.1345 | 0.0137 | 1.23E-22 | 96.38 |
| 6 | rs186856025 | T | C | 0.0596 | -0.1571 | 0.0248 | 2.32E-10 | 40.13 |
| 7 | rs2325259 | C | T | 0.2511 | -0.0758 | 0.0134 | 1.69E-08 | 32.00 |
| 8 | rs35656734 | T | C | 0.4264 | -0.1122 | 0.0131 | 1.00E-17 | 73.36 |
| 9 | rs60227565 | A | G | 0.1539 | -0.1189 | 0.0161 | 1.74E-13 | 54.54 |
| 10 | rs62192043 | A | G | 0.2335 | -0.1015 | 0.0140 | 3.51E-13 | 52.56 |
| 11 | rs6894249 | G | A | 0.4652 | 0.0932 | 0.0116 | 9.29E-16 | 64.55 |
| 12 | rs7035413 | G | A | 0.1886 | 0.1302 | 0.0148 | 1.69E-18 | 77.39 |
| 13 | rs74630264 | A | G | 0.0816 | -0.1501 | 0.0214 | 2.49E-12 | 49.20 |
| 14 | rs8074437 | G | T | 0.4464 | 0.0981 | 0.0117 | 4.17E-17 | 70.30 |

SNPs, single nucleotide polymorphisms; EAF, effect allele frequency; SE, standard error.

**Supplementary Table 11: Characteristics of 14 SNPs used as instrumental variables for the effect of asthma on Right leg fat mass.**

|  | SNP | effect_allele.exposure | other_allele.exposure | EAF.exposure | effect | SE | *P*-value | F-Statistic |
| --- | --- | --- | --- | --- | --- | --- | --- | --- |
| 1 | rs11137222 | T | C | 0.0357 | 0.1724 | 0.0314 | 4.01E-08 | 30.14 |
| 2 | rs11667612 | T | G | 0.0528 | 0.1471 | 0.0259 | 1.43E-08 | 32.26 |
| 3 | rs118013485 | A | G | 0.0984 | -0.1108 | 0.0197 | 1.92E-08 | 31.63 |
| 4 | rs17293632 | T | C | 0.2622 | 0.0990 | 0.0131 | 4.66E-14 | 57.11 |
| 5 | rs1837253 | C | T | 0.7550 | 0.1345 | 0.0137 | 1.23E-22 | 96.38 |
| 6 | rs186856025 | T | C | 0.0596 | -0.1571 | 0.0248 | 2.32E-10 | 40.13 |
| 7 | rs2325259 | C | T | 0.2511 | -0.0758 | 0.0134 | 1.69E-08 | 32.00 |
| 8 | rs35656734 | T | C | 0.4264 | -0.1122 | 0.0131 | 1.00E-17 | 73.36 |
| 9 | rs60227565 | A | G | 0.1539 | -0.1189 | 0.0161 | 1.74E-13 | 54.54 |
| 10 | rs62192043 | A | G | 0.2335 | -0.1015 | 0.0140 | 3.51E-13 | 52.56 |
| 11 | rs6894249 | G | A | 0.4652 | 0.0932 | 0.0116 | 9.29E-16 | 64.55 |
| 12 | rs7035413 | G | A | 0.1886 | 0.1302 | 0.0148 | 1.69E-18 | 77.39 |
| 13 | rs74630264 | A | G | 0.0816 | -0.1501 | 0.0214 | 2.49E-12 | 49.20 |
| 14 | rs8074437 | G | T | 0.4464 | 0.0981 | 0.0117 | 4.17E-17 | 70.30 |

SNPs, single nucleotide polymorphisms; EAF, effect allele frequency; SE, standard error.

**Supplementary Table 12: Characteristics of 15 SNPs used as instrumental variables for the effect of asthma on Trunk fat mass.**

|  | SNP | effect_allele.exposure | other_allele.exposure | EAF.exposure | effect | SE | *P*-value | F-Statistic |
| --- | --- | --- | --- | --- | --- | --- | --- | --- |
| 1 | rs11137222 | T | C | 0.0357 | 0.1724 | 0.0314 | 4.01E-08 | 30.14 |
| 2 | rs11667612 | T | G | 0.0528 | 0.1471 | 0.0259 | 1.43E-08 | 32.26 |
| 3 | rs118013485 | A | G | 0.0984 | -0.1108 | 0.0197 | 1.92E-08 | 31.63 |
| 4 | rs12761415 | G | A | 0.1678 | 0.0857 | 0.0155 | 3.17E-08 | 30.57 |
| 5 | rs17293632 | T | C | 0.2622 | 0.0990 | 0.0131 | 4.66E-14 | 57.11 |
| 6 | rs1837253 | C | T | 0.7550 | 0.1345 | 0.0137 | 1.23E-22 | 96.38 |
| 7 | rs186856025 | T | C | 0.0596 | -0.1571 | 0.0248 | 2.32E-10 | 40.13 |
| 8 | rs2325259 | C | T | 0.2511 | -0.0758 | 0.0134 | 1.69E-08 | 32.00 |
| 9 | rs35656734 | T | C | 0.4264 | -0.1122 | 0.0131 | 1.00E-17 | 73.36 |
| 10 | rs60227565 | A | G | 0.1539 | -0.1189 | 0.0161 | 1.74E-13 | 54.54 |
| 11 | rs62192043 | A | G | 0.2335 | -0.1015 | 0.0140 | 3.51E-13 | 52.56 |
| 12 | rs6894249 | G | A | 0.4652 | 0.0932 | 0.0116 | 9.29E-16 | 64.55 |
| 13 | rs7035413 | G | A | 0.1886 | 0.1302 | 0.0148 | 1.69E-18 | 77.39 |
| 14 | rs74630264 | A | G | 0.0816 | -0.1501 | 0.0214 | 2.49E-12 | 49.20 |
| 15 | rs8074437 | G | T | 0.4464 | 0.0981 | 0.0117 | 4.17E-17 | 70.30 |

SNPs, single nucleotide polymorphisms; EAF, effect allele frequency; SE, standard error.

**Supplementary Table 13: Characteristics of 14 SNPs used as instrumental variables for the effect of asthma on Total fat mass.**

|  | SNP | effect_allele.exposure | other_allele.exposure | EAF.exposure | effect | SE | *P*-value | F-Statistic |
| --- | --- | --- | --- | --- | --- | --- | --- | --- |
| 1 | rs11137222 | T | C | 0.0357 | 0.1724 | 0.0314 | 4.01E-08 | 30.14 |
| 2 | rs11667612 | T | G | 0.0528 | 0.1471 | 0.0259 | 1.43E-08 | 32.26 |
| 3 | rs118013485 | A | G | 0.0984 | -0.1108 | 0.0197 | 1.92E-08 | 31.63 |
| 4 | rs17293632 | T | C | 0.2622 | 0.0990 | 0.0131 | 4.66E-14 | 57.11 |
| 5 | rs1837253 | C | T | 0.7550 | 0.1345 | 0.0137 | 1.23E-22 | 96.38 |
| 6 | rs186856025 | T | C | 0.0596 | -0.1571 | 0.0248 | 2.32E-10 | 40.13 |
| 7 | rs2325259 | C | T | 0.2511 | -0.0758 | 0.0134 | 1.69E-08 | 32.00 |
| 8 | rs35656734 | T | C | 0.4264 | -0.1122 | 0.0131 | 1.00E-17 | 73.36 |
| 9 | rs60227565 | A | G | 0.1539 | -0.1189 | 0.0161 | 1.74E-13 | 54.54 |
| 10 | rs62192043 | A | G | 0.2335 | -0.1015 | 0.0140 | 3.51E-13 | 52.56 |
| 11 | rs6894249 | G | A | 0.4652 | 0.0932 | 0.0116 | 9.29E-16 | 64.55 |
| 12 | rs7035413 | G | A | 0.1886 | 0.1302 | 0.0148 | 1.69E-18 | 77.39 |
| 13 | rs74630264 | A | G | 0.0816 | -0.1501 | 0.0214 | 2.49E-12 | 49.20 |
| 14 | rs8074437 | G | T | 0.4464 | 0.0981 | 0.0117 | 4.17E-17 | 70.30 |

SNPs, single nucleotide polymorphisms; EAF, effect allele frequency; SE, standard error.
