# Supplementary material for: Association between body fat distribution and asthma in adults: results from the cross-sectional and bidirectional Mendelian randomization study
Source: Front Nutr. 2024 Jul 22;11:1432973. doi: 10.3389/fnut.2024.1432973 (PMC11299241; doi:10.3389/fnut.2024.1432973)

Supplementary Figure 1. Display of the forest plot for the single SNP analysis of Left arm fat mass increasing on asthma risk. MR, mendelian randomization; SNPs, single nucleotide polymorphisms.

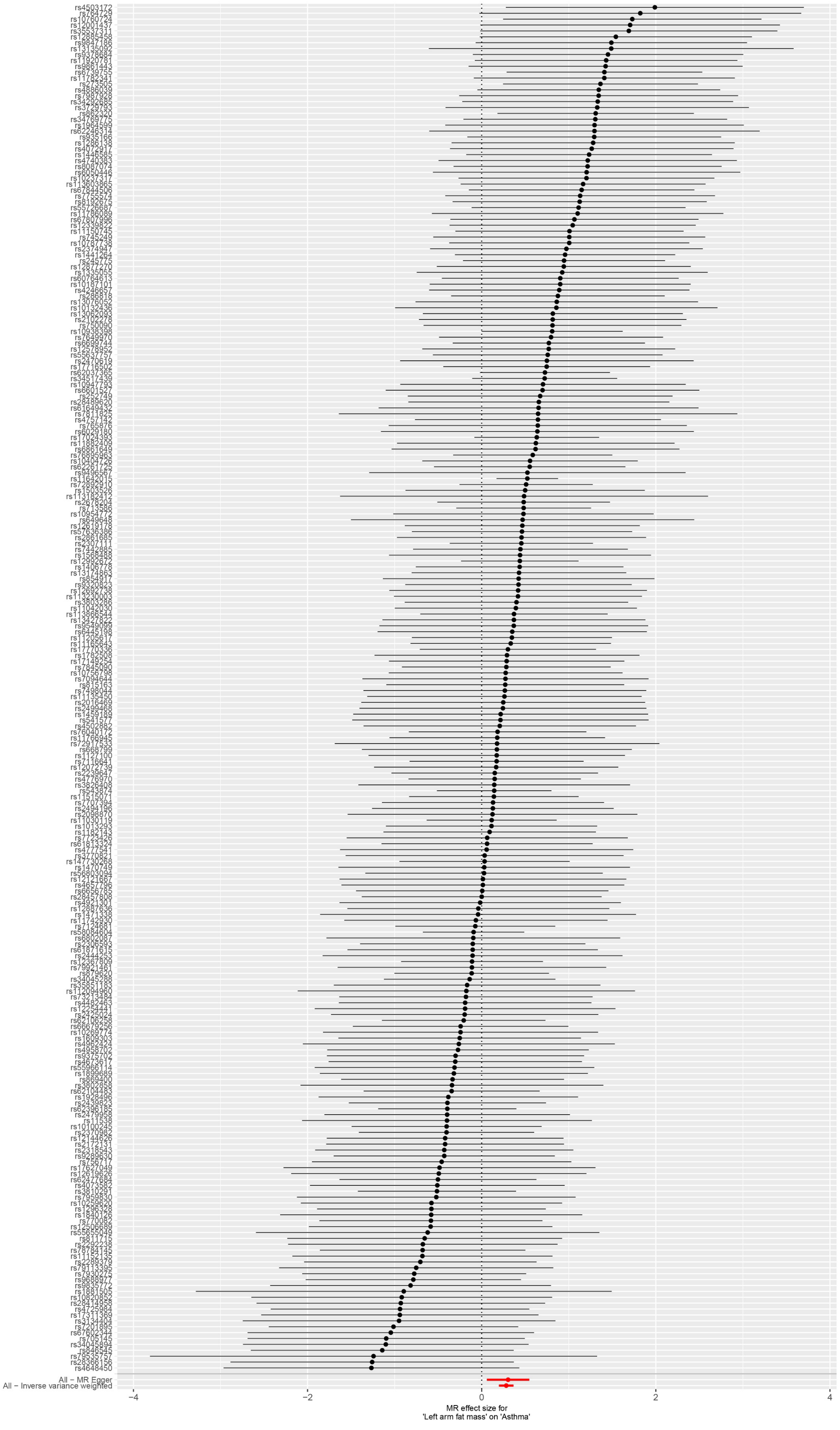

Supplementary Figure 2. Display of the forest plot for the single SNP analysis of Left leg fat mass increasing on asthma risk. MR, mendelian randomization; SNPs, single nucleotide polymorphisms.

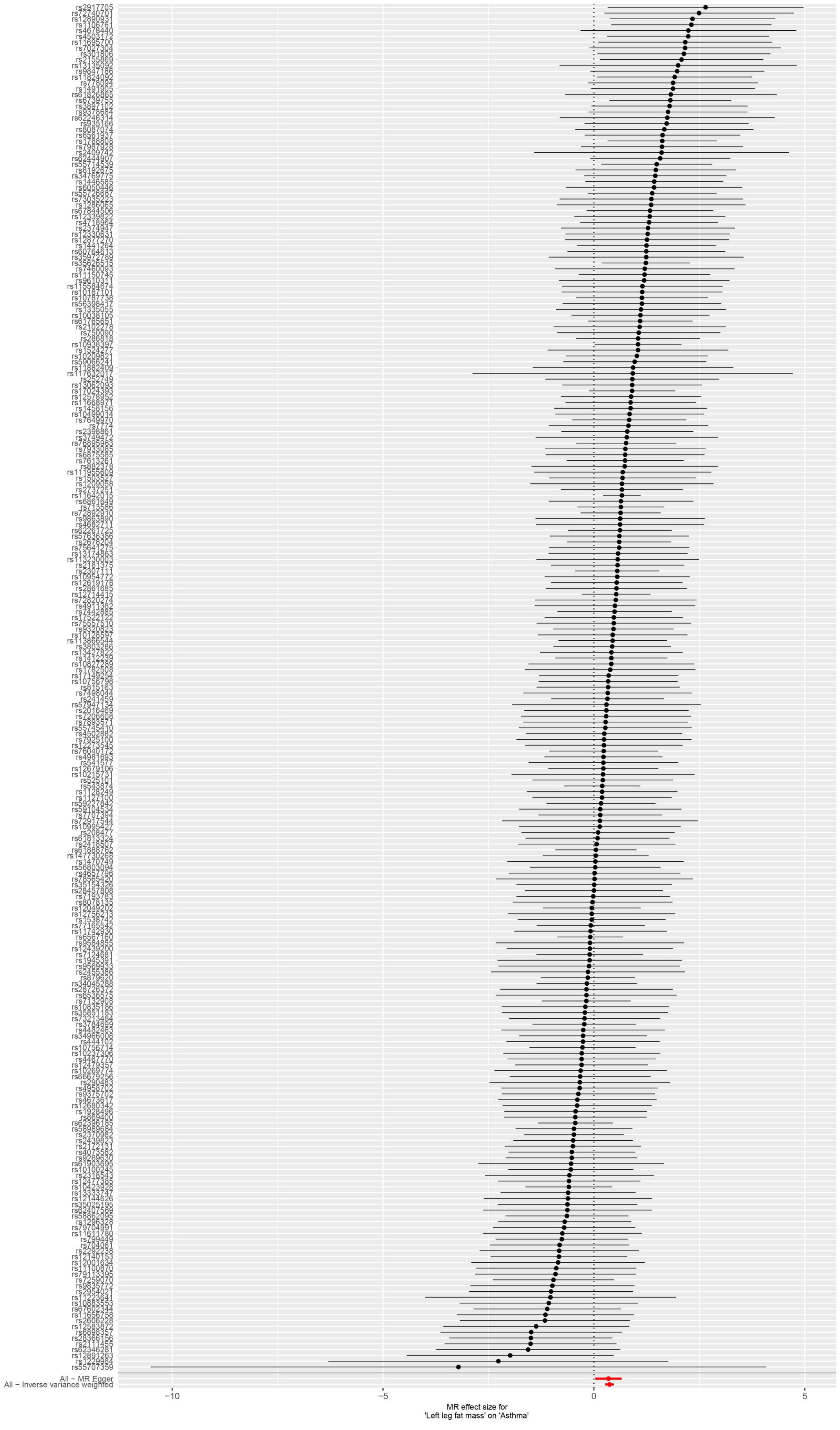

Supplementary Figure 3. Display of the forest plot for the single SNP analysis of Right arm fat mass increasing on asthma risk. MR, mendelian randomization; SNPs, single nucleotide polymorphisms.

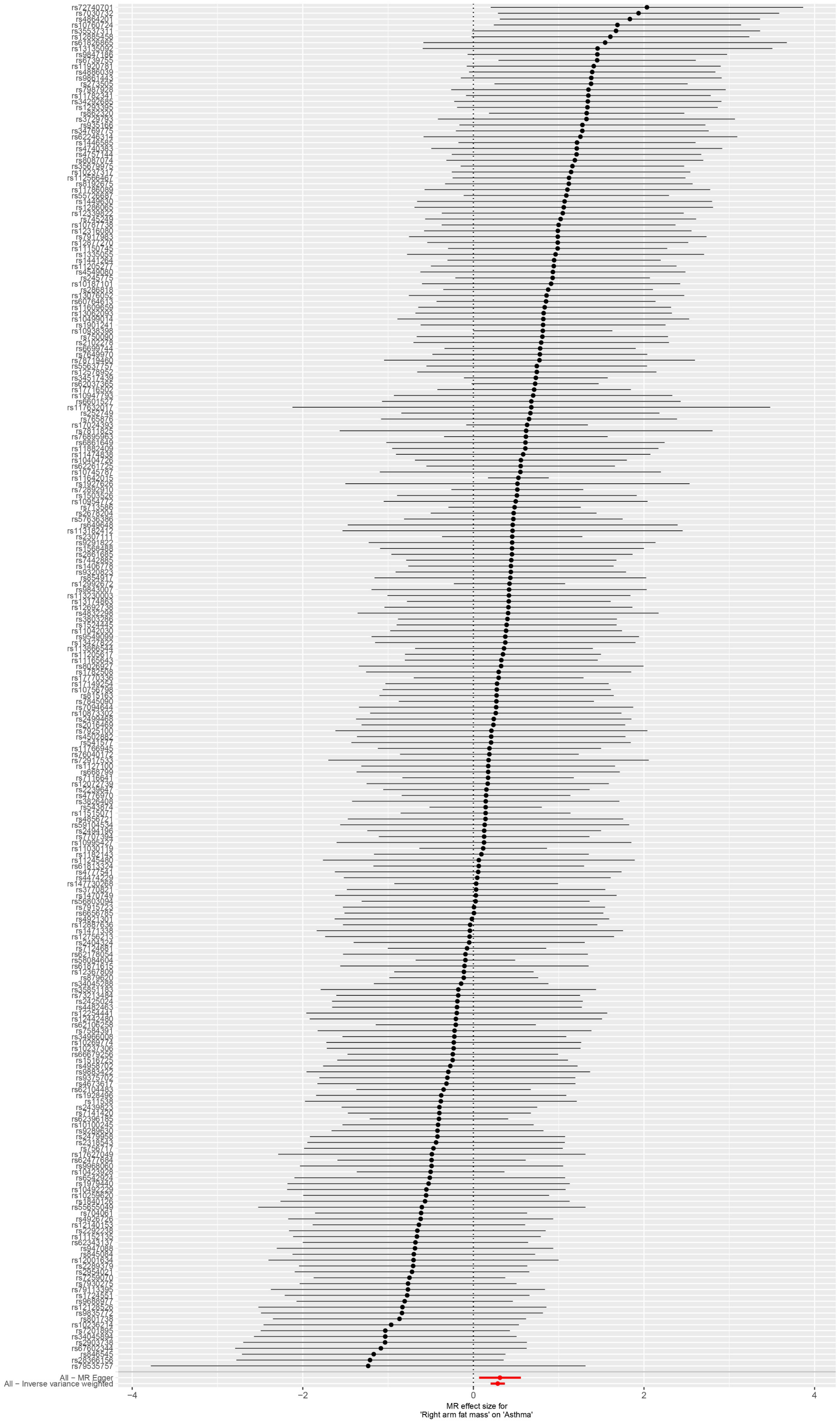

Supplementary Figure 4. Display of the forest plot for the single SNP analysis of Right leg fat mass increasing on asthma risk. MR, mendelian randomization; SNPs, single nucleotide polymorphisms.

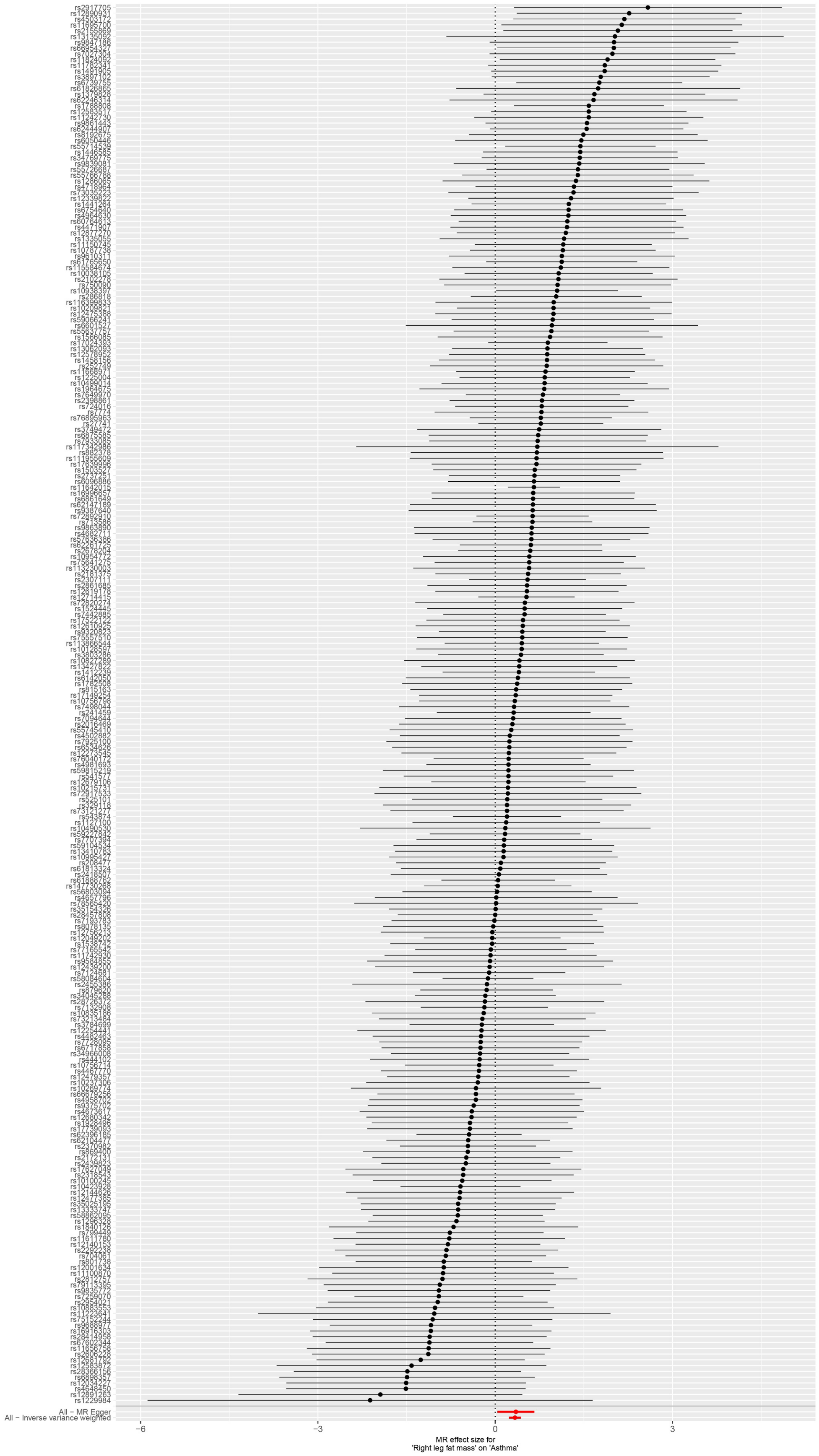

Supplementary Figure 5. Display of the forest plot for the single SNP analysis of Trunk fat mass increasing on asthma risk. MR, mendelian randomization; SNPs, single nucleotide polymorphisms.

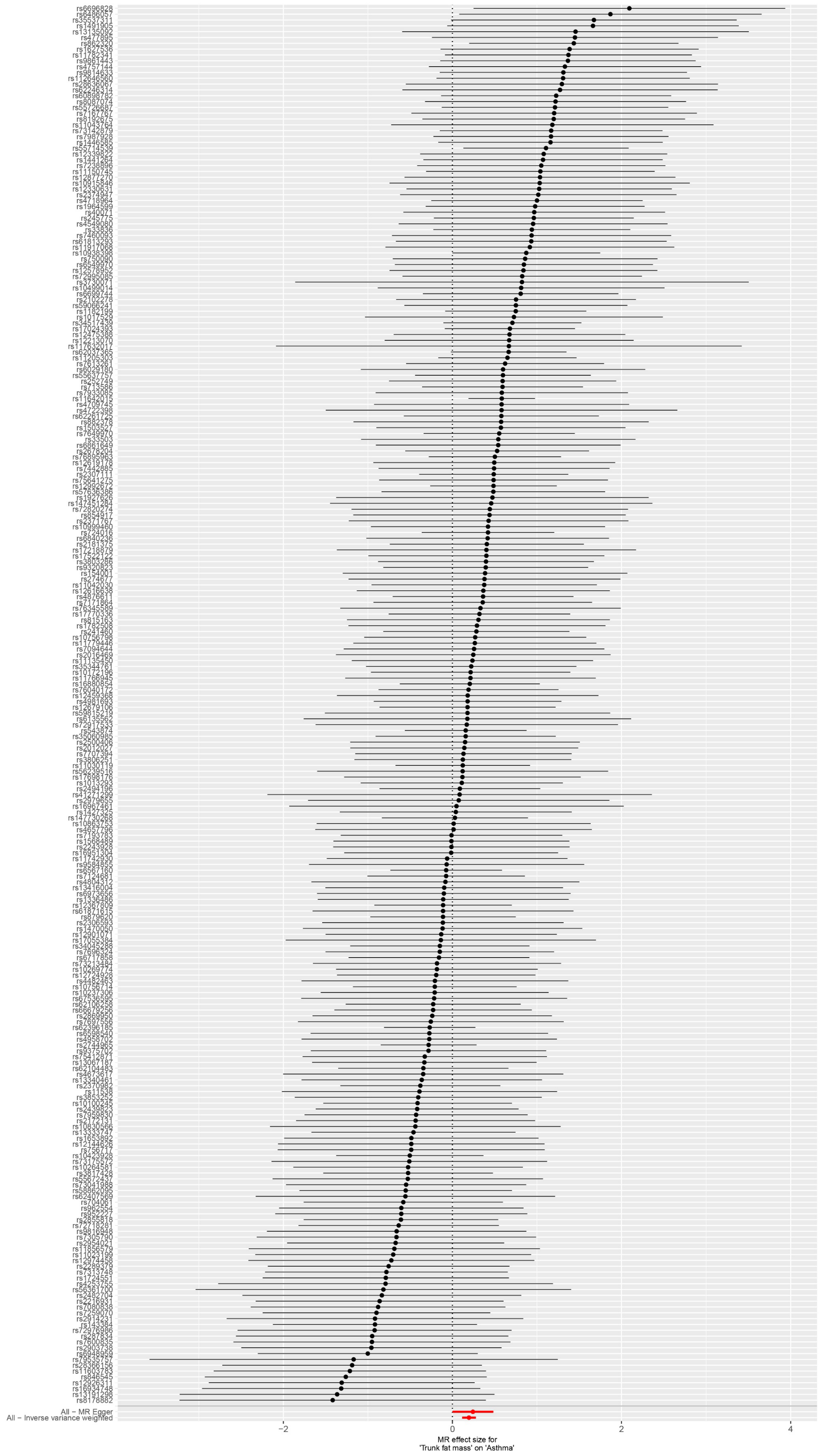

Supplementary Figure 6. Display of the forest plot for the single SNP analysis of Total fat mass increasing on asthma risk. MR, mendelian randomization; SNPs, single nucleotide polymorphisms.

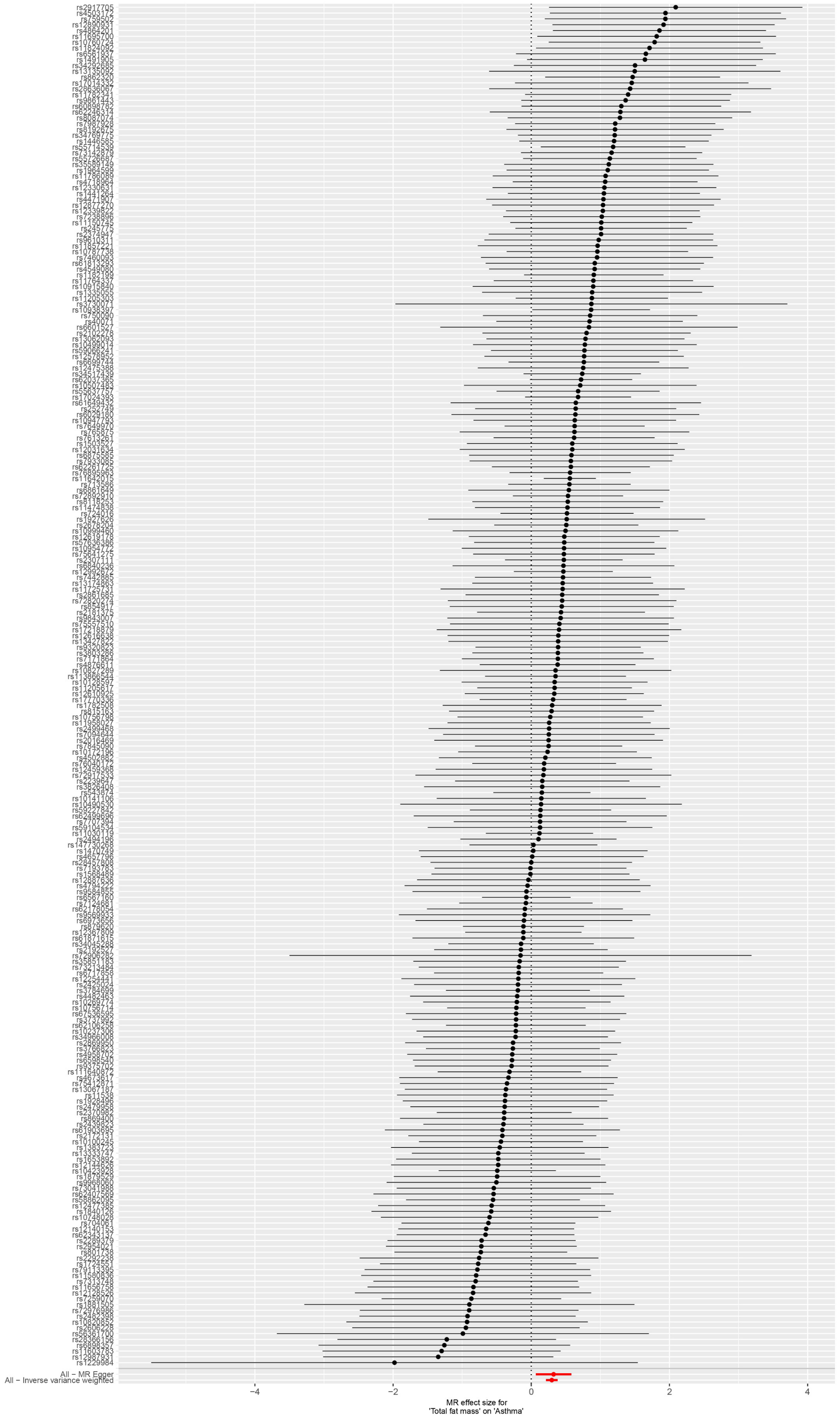

Supplementary Figure 7. Scatter plot of SNPs associated with body fat indexes on asthma risk. A, Left arm fat mass; B, Left leg fat mass; C, Right arm fat mass; D, Right leg fat mass; E, Trunk fat mass; F, Total fat mass. MR, mendelian randomization; SNPs, single nucleotide polymorphisms.

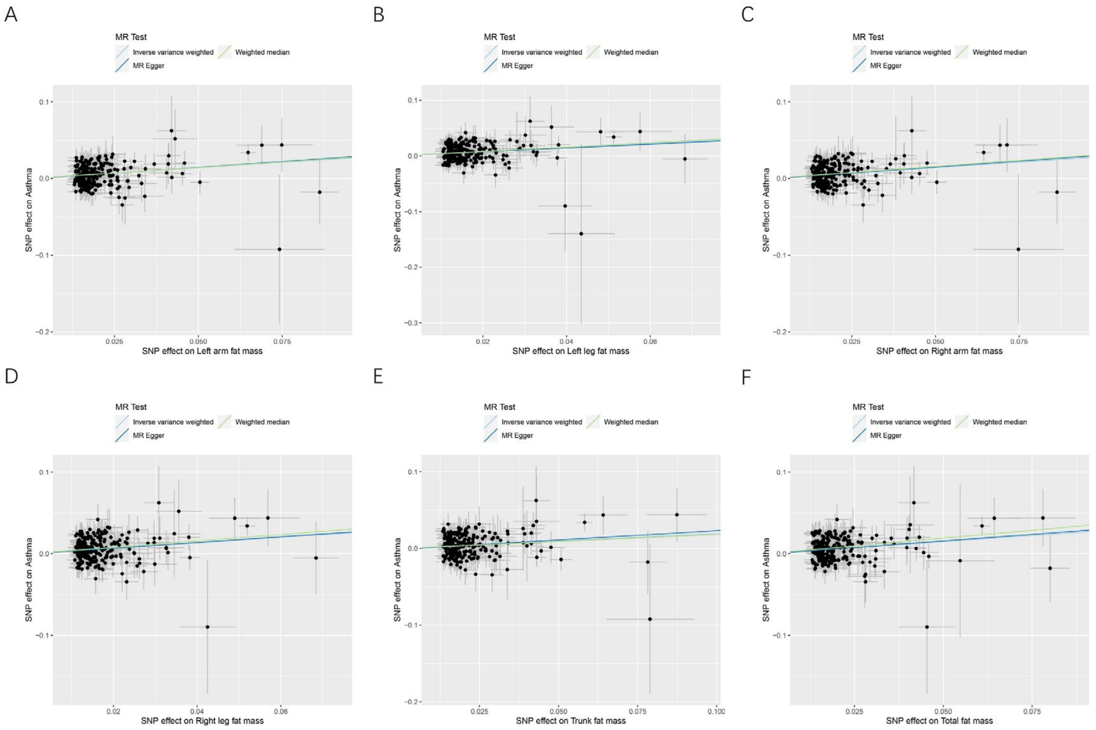

Supplementary Figure 8. Leave-one-out analyses for SNPs associated with Left arm fat mass increasing on asthma risk. MR, mendelian randomization; SNPs, single nucleotide polymorphisms.

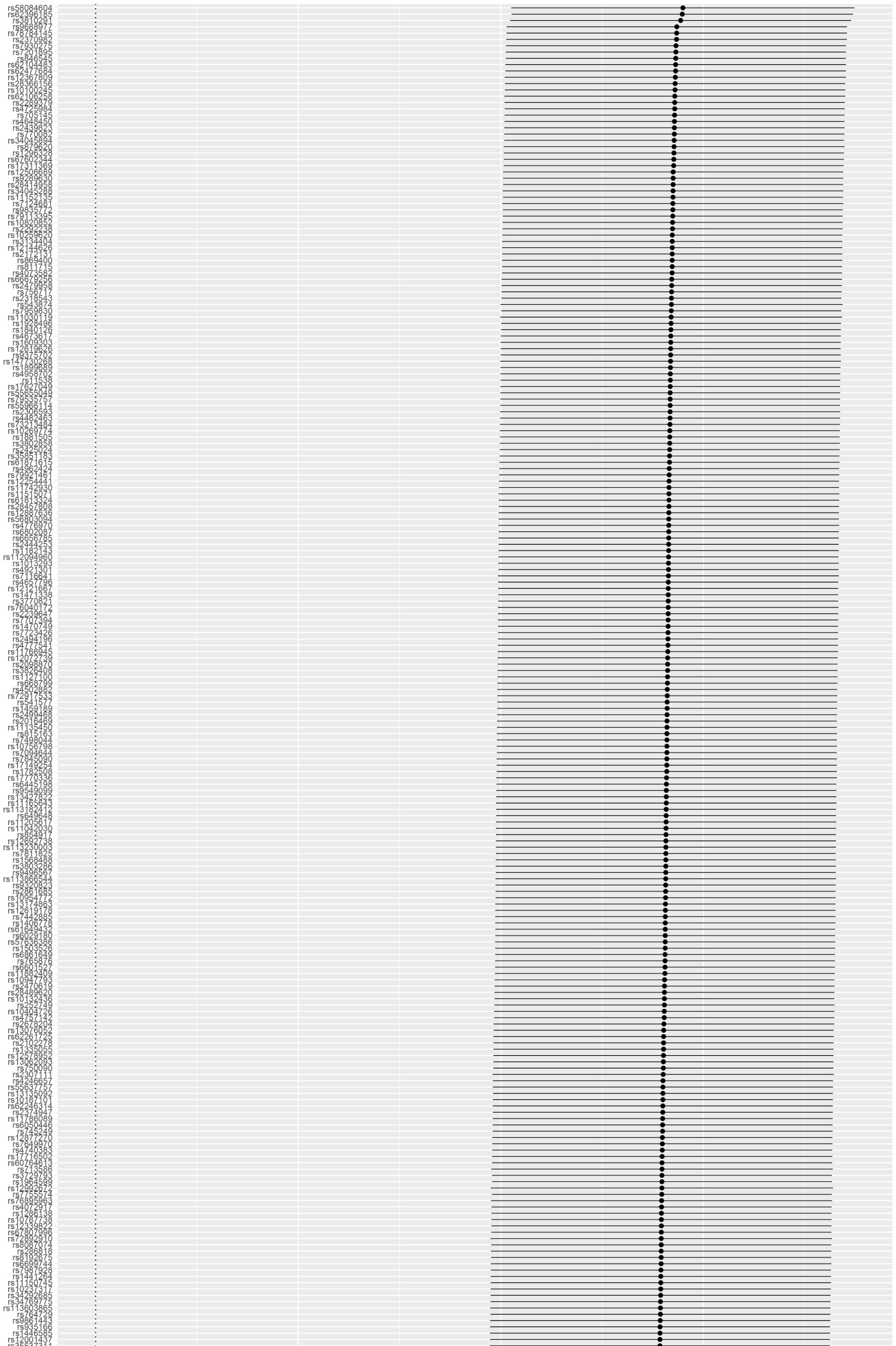

Supplementary Figure 9. Leave-one-out analyses for SNPs associated with Left leg fat mass increasing on asthma risk. MR, mendelian randomization; SNPs, single nucleotide polymorphisms.

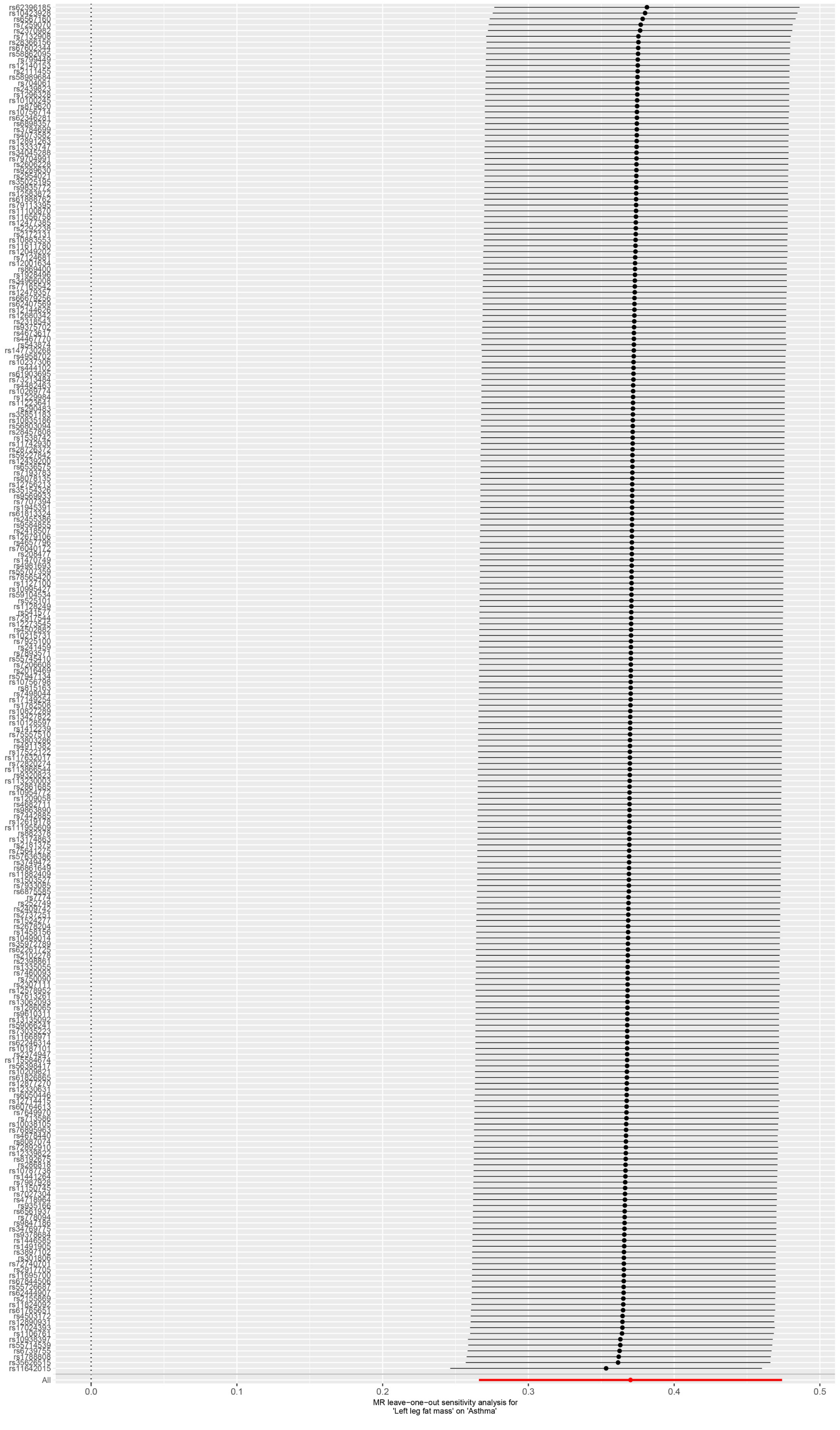

Supplementary Figure 10. Leave-one-out analyses for SNPs associated with Right arm fat mass increasing on asthma risk. MR, mendelian randomization; SNPs, single nucleotide polymorphisms.

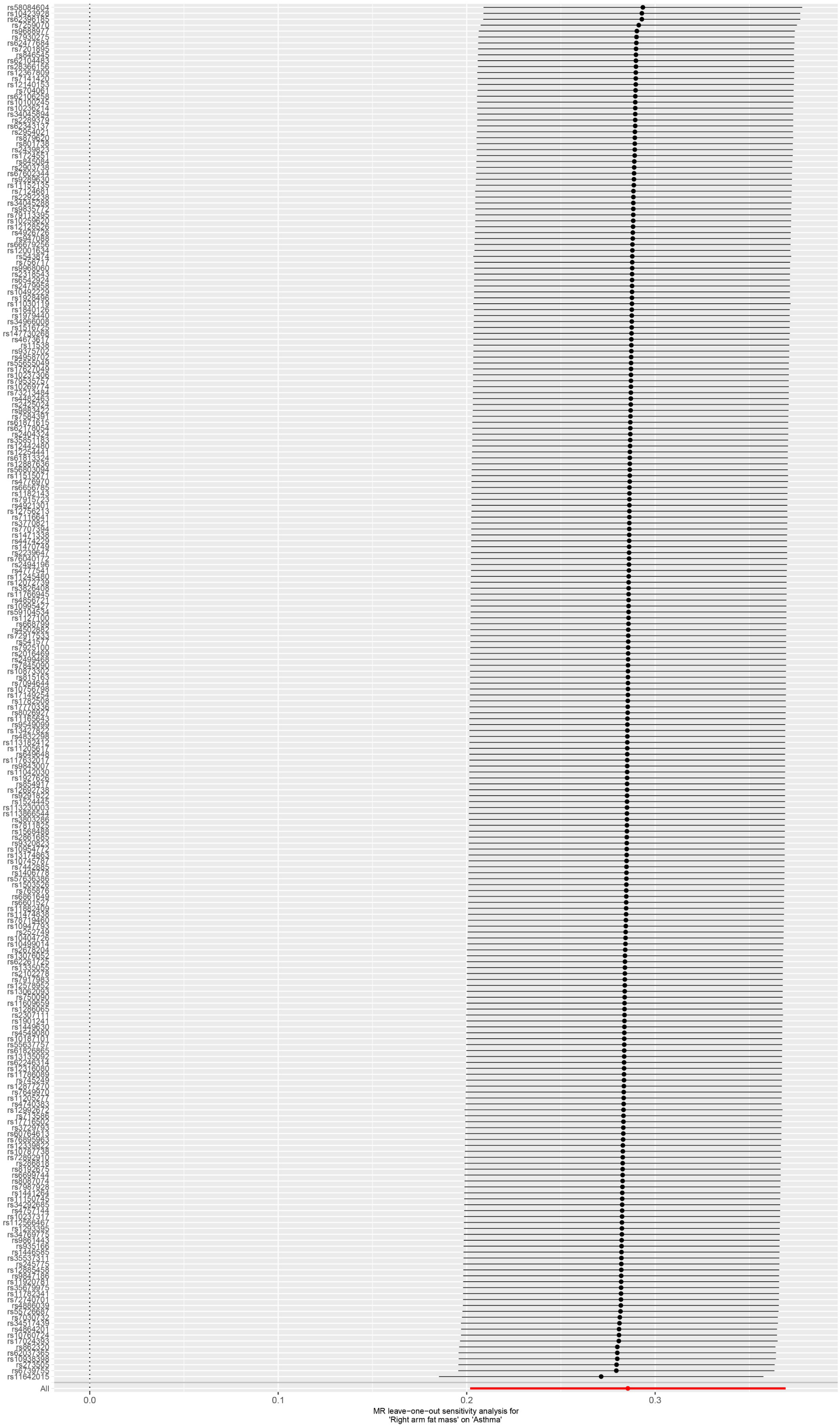

Supplementary Figure 11. Leave-one-out analyses for SNPs associated with Right leg fat mass increasing on asthma risk. MR, mendelian randomization; SNPs, single nucleotide polymorphisms.

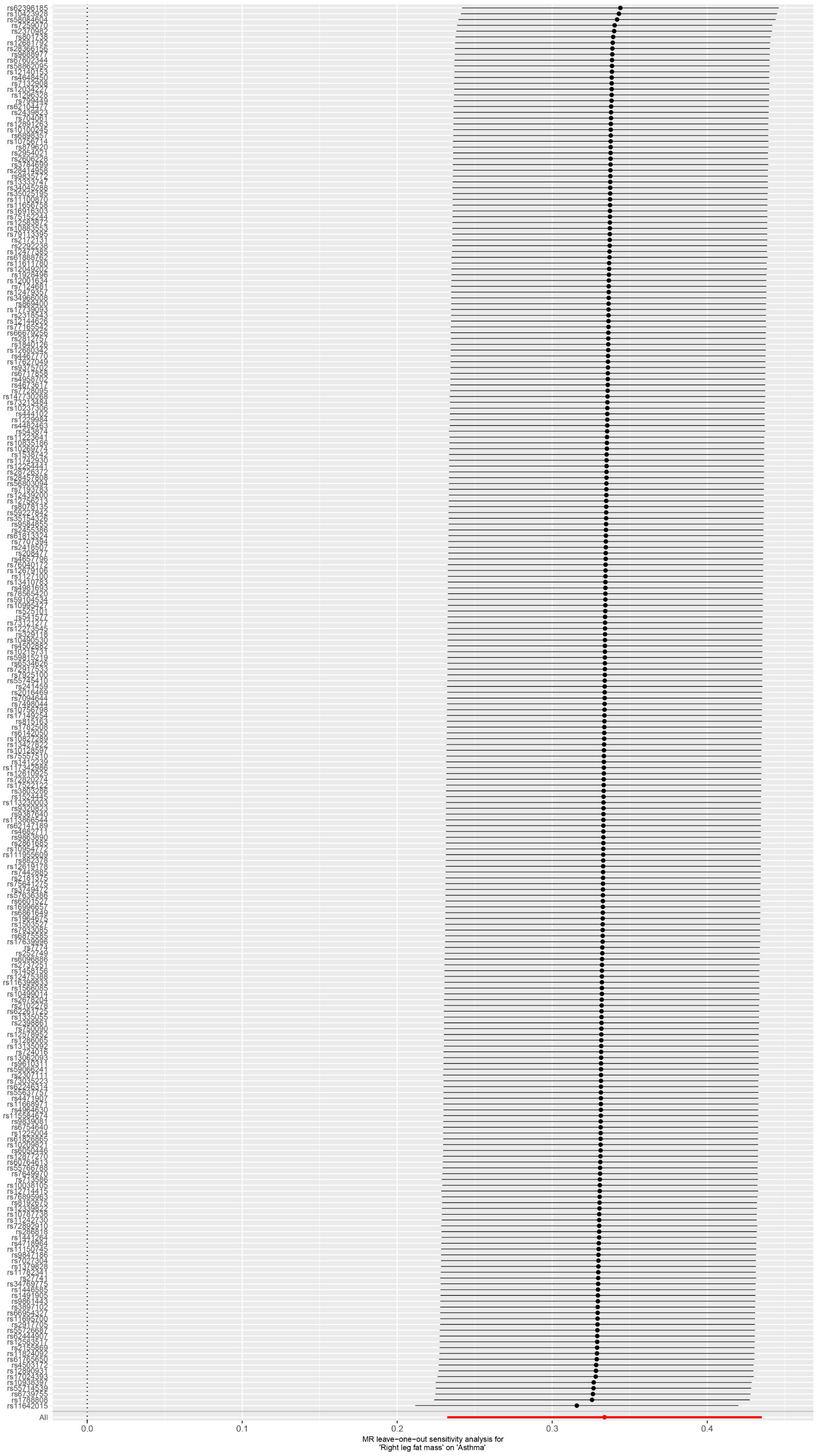

Supplementary Figure 12. Leave-one-out analyses for SNPs associated with Trunk fat mass increasing on asthma risk. MR, mendelian randomization; SNPs, single nucleotide polymorphisms.

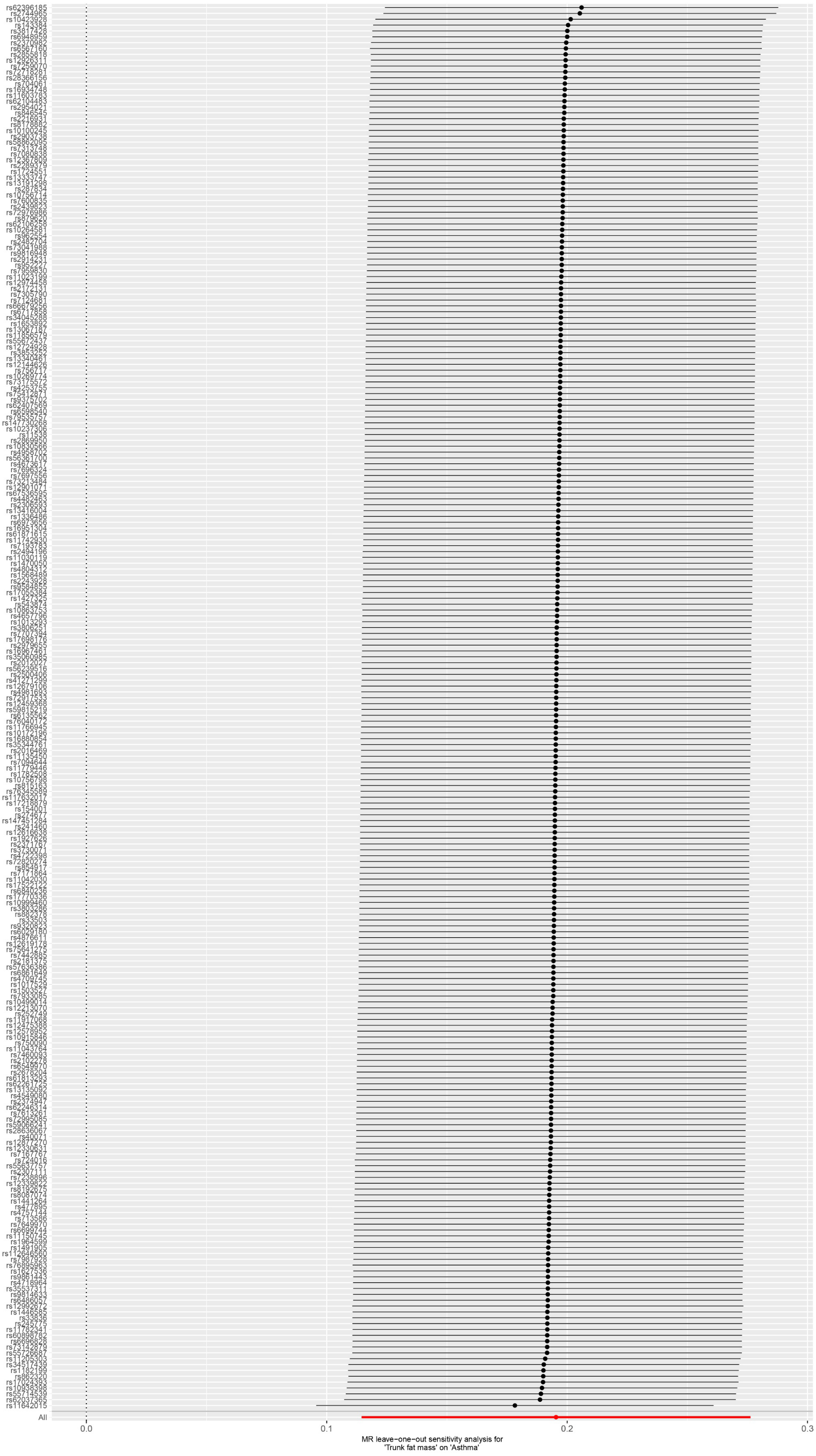

Supplementary Figure 13. Leave-one-out analyses for SNPs associated with Total fat mass increasing on asthma risk. MR, mendelian randomization; SNPs, single nucleotide polymorphisms.

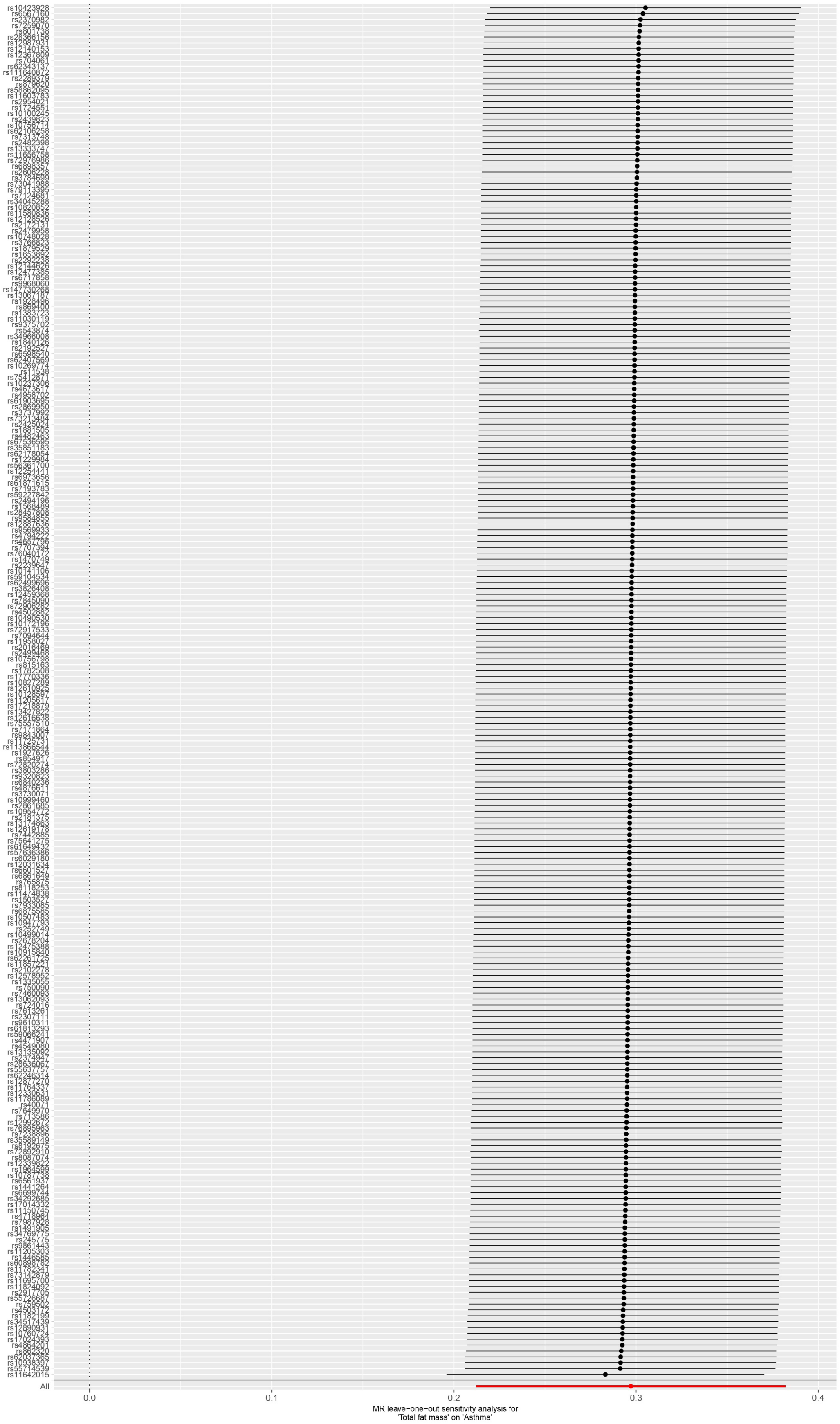

Supplementary Figure 14. Funnel plot of SNPs associated with body fat indexes on asthma risk. A, Left arm fat mass; B, Left leg fat mass; C, Right arm fat mass; D, Right leg fat mass; E, Trunk fat mass; F, Total fat mass. MR, mendelian randomization; SNPs, single nucleotide polymorphisms.

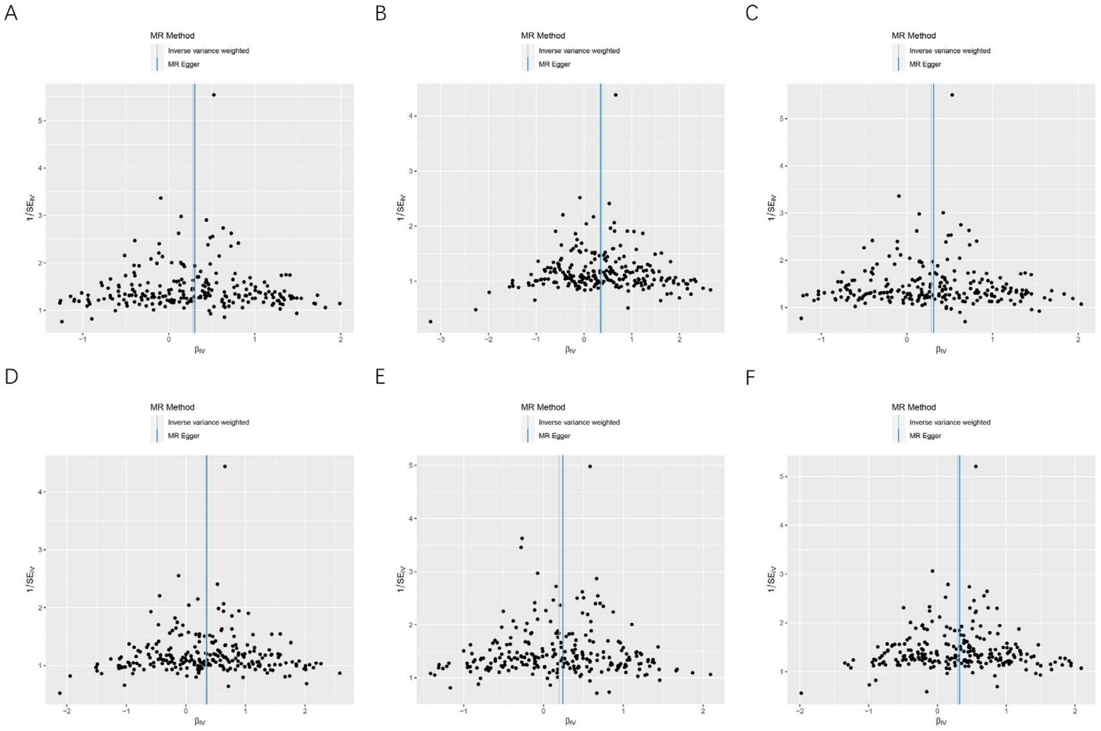

Supplementary Figure 15. Display of the forest plot for the single SNP analysis of asthma on Left arm fat mass. MR, mendelian randomization; SNPs, single nucleotide polymorphisms.

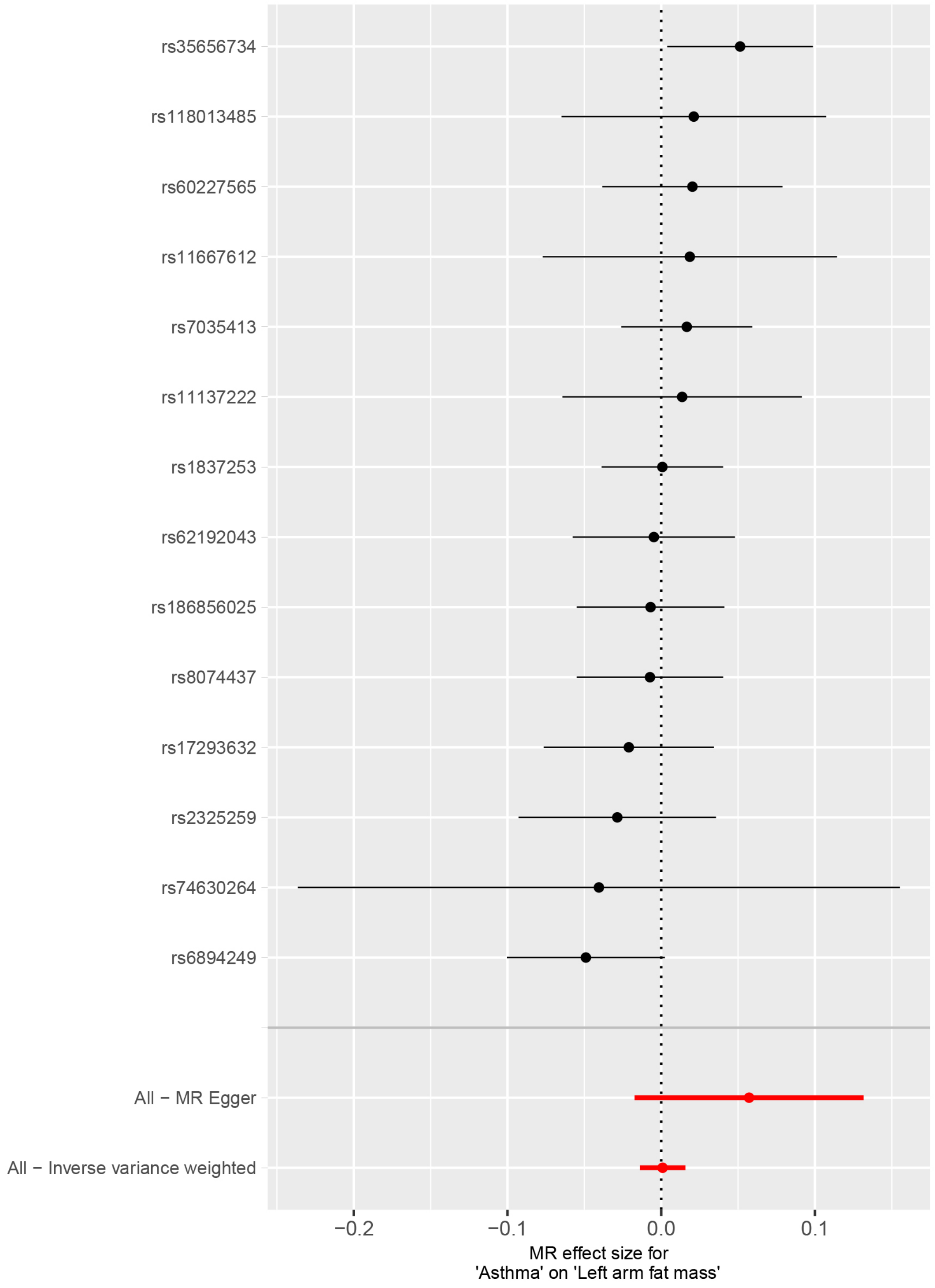

Supplementary Figure 16. Display of the forest plot for the single SNP analysis of asthma on Left leg fat mass. MR, mendelian randomization; SNPs, single nucleotide polymorphisms.

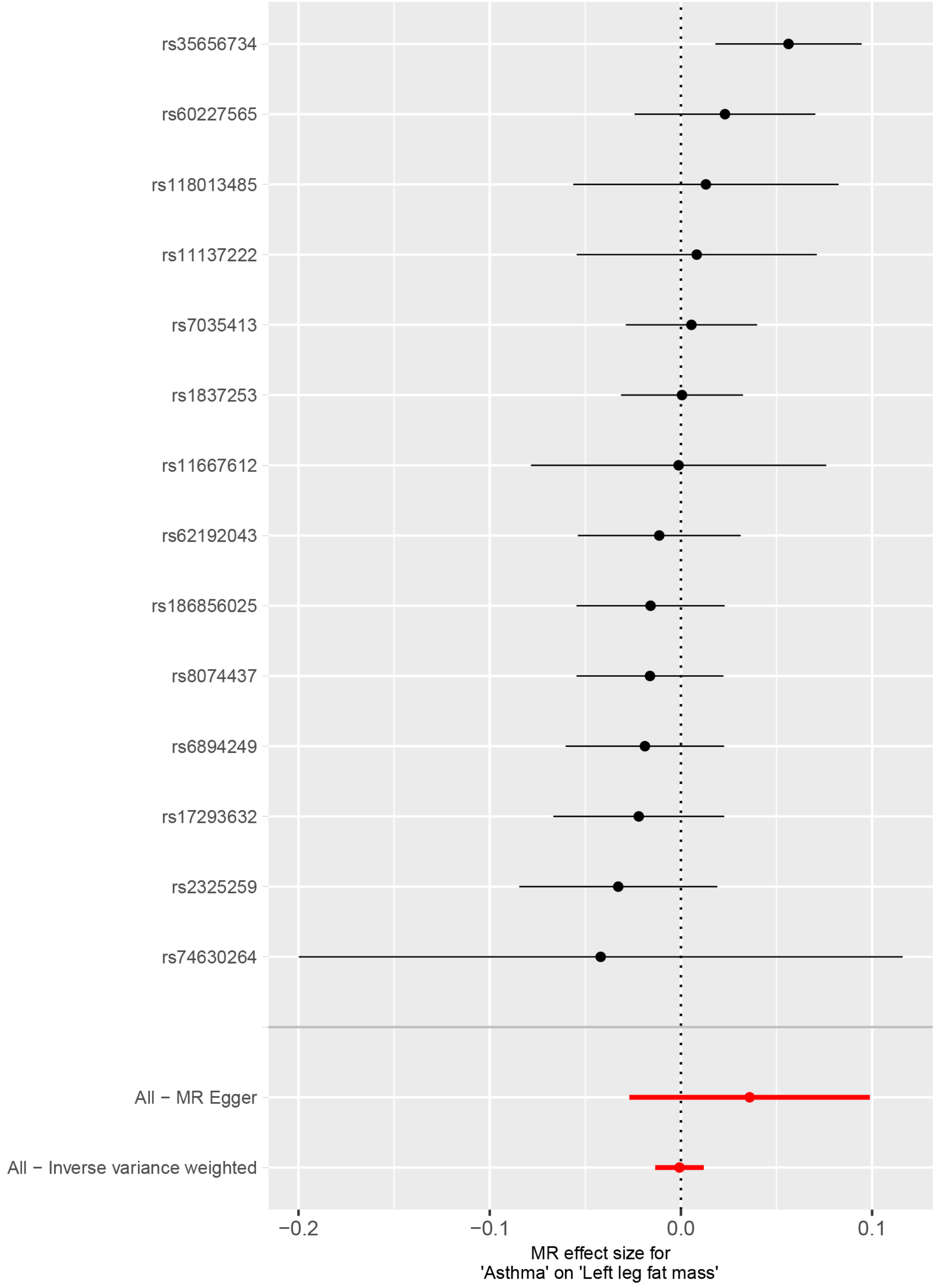

Supplementary Figure 17. Display of the forest plot for the single SNP analysis of asthma on Right arm fat mass. MR, mendelian randomization; SNPs, single nucleotide polymorphisms.

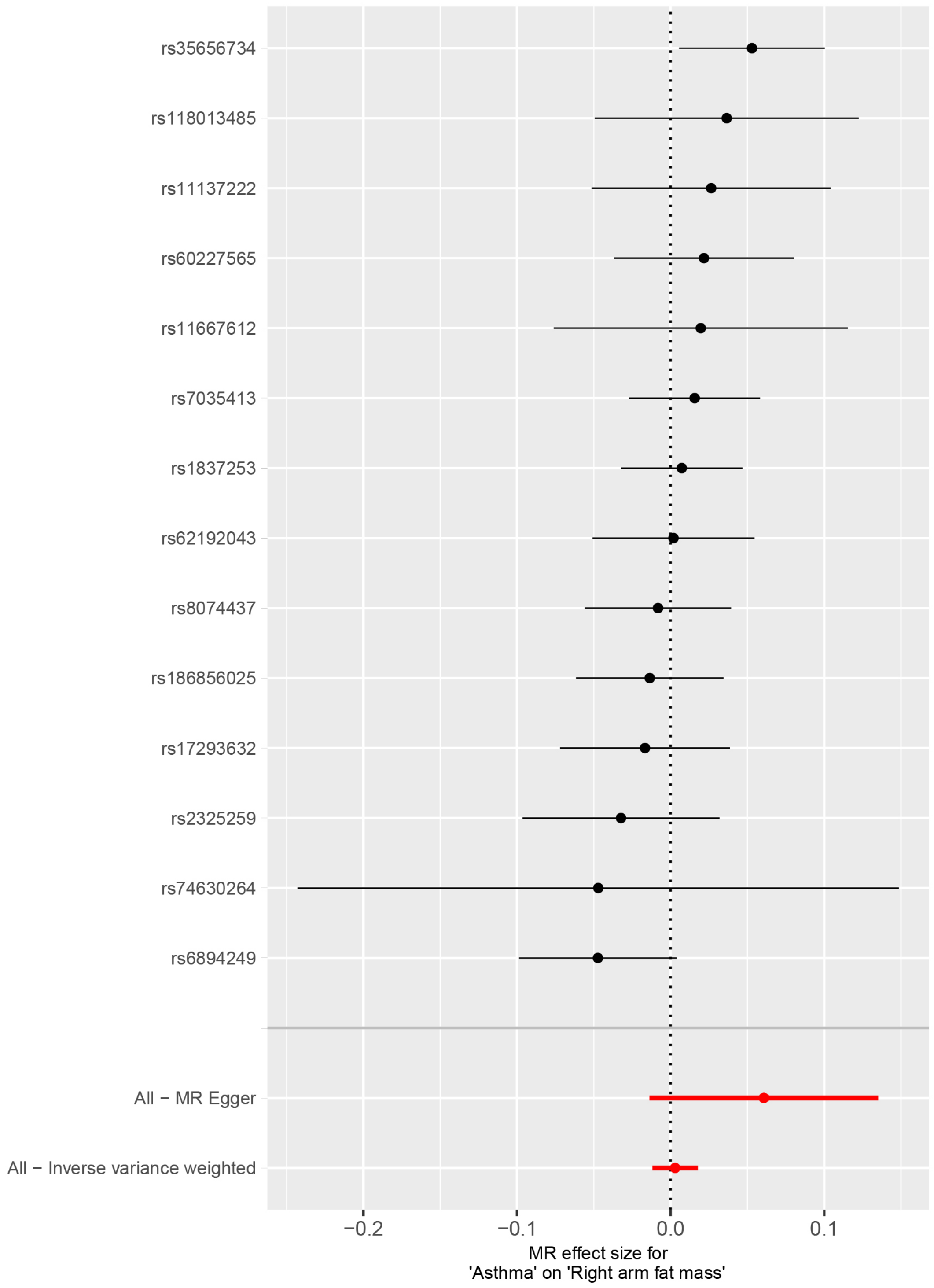

Supplementary Figure 18. Display of the forest plot for the single SNP analysis of asthma on Right leg fat mass. MR, mendelian randomization; SNPs, single nucleotide polymorphisms.

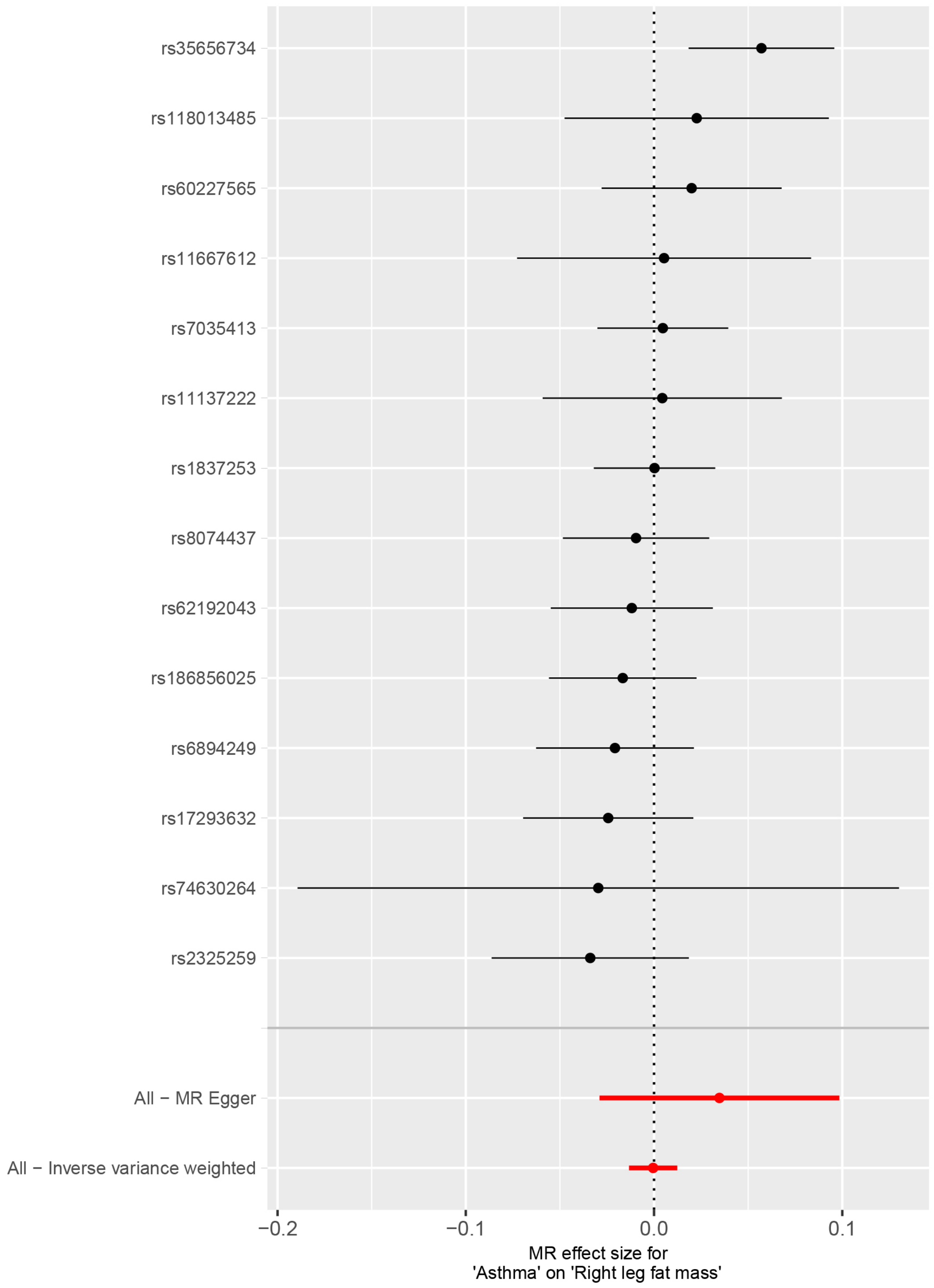

Supplementary Figure 19. Display of the forest plot for the single SNP analysis of asthma on Trunk fat mass. MR, mendelian randomization; SNPs, single nucleotide polymorphisms.

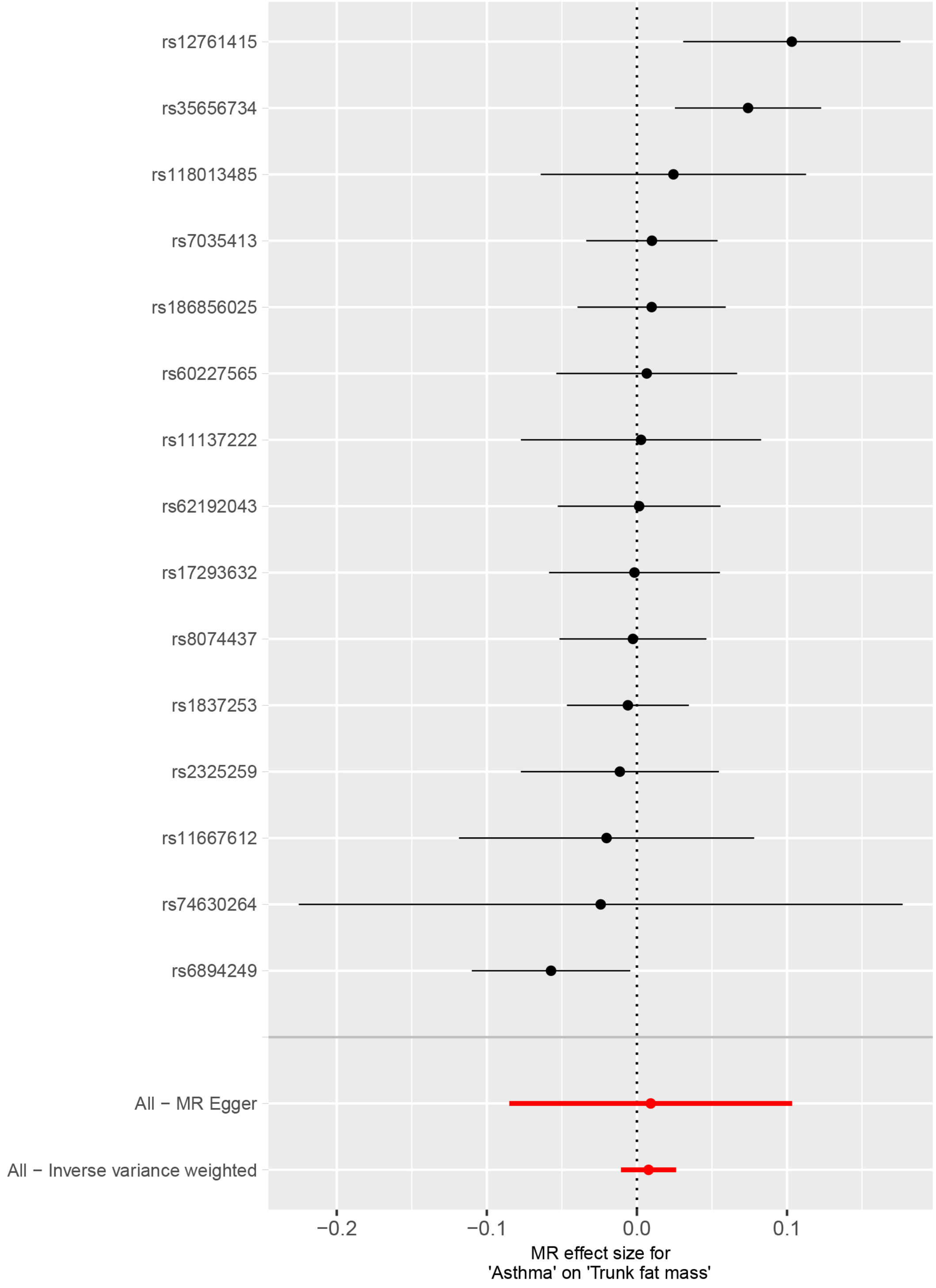

Supplementary Figure 20. Display of the forest plot for the single SNP analysis of asthma on Total fat mass. MR, mendelian randomization; SNPs, single nucleotide polymorphisms.

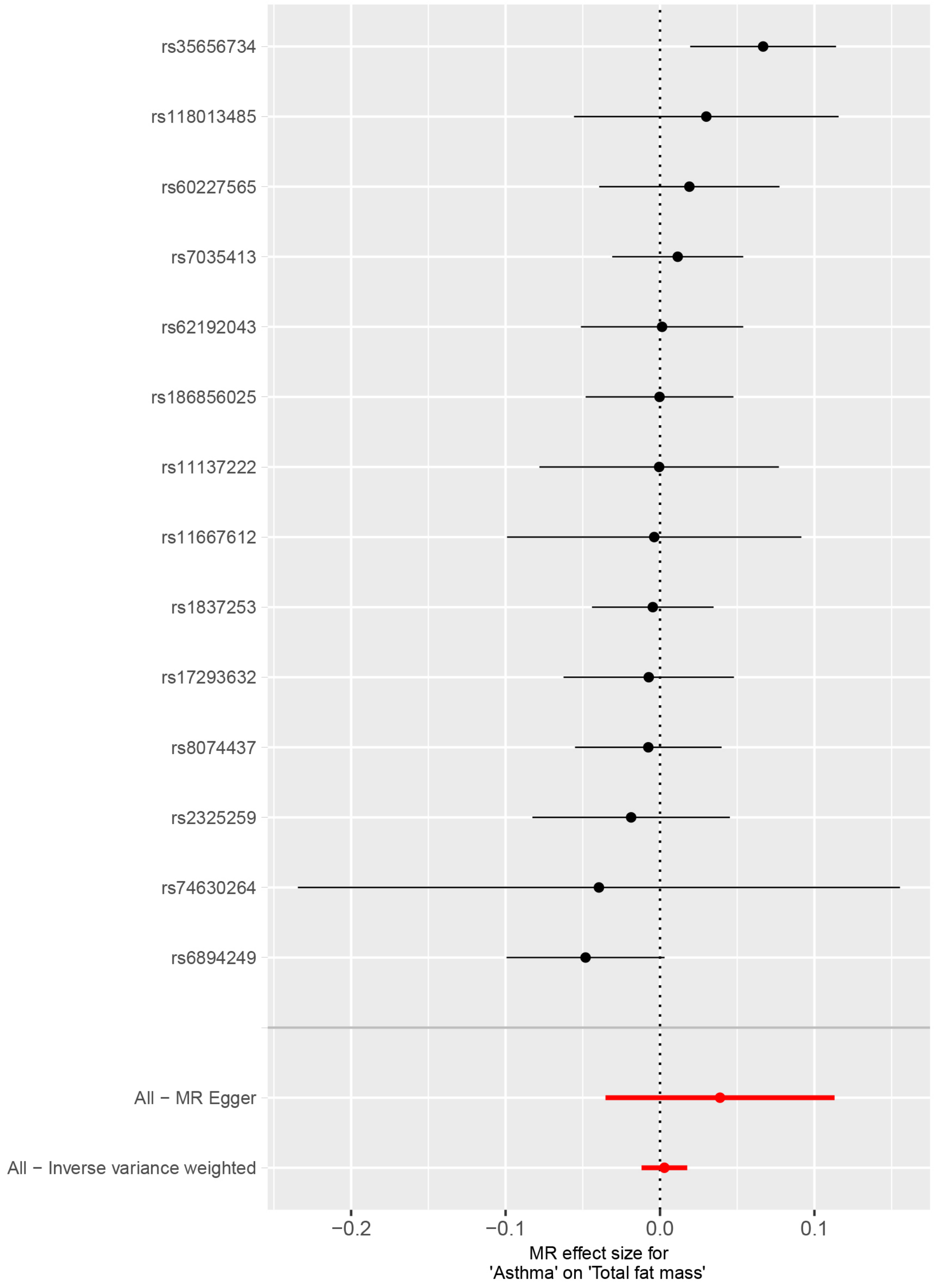

Supplementary Figure 21. Scatter plot of SNPs associated with asthma on body fat indexes. A, Left arm fat mass; B, Left leg fat mass; C, Right arm fat mass; D, Right leg fat mass; E, Trunk fat mass; F, Total fat mass. MR, mendelian randomization; SNPs, single nucleotide polymorphisms.

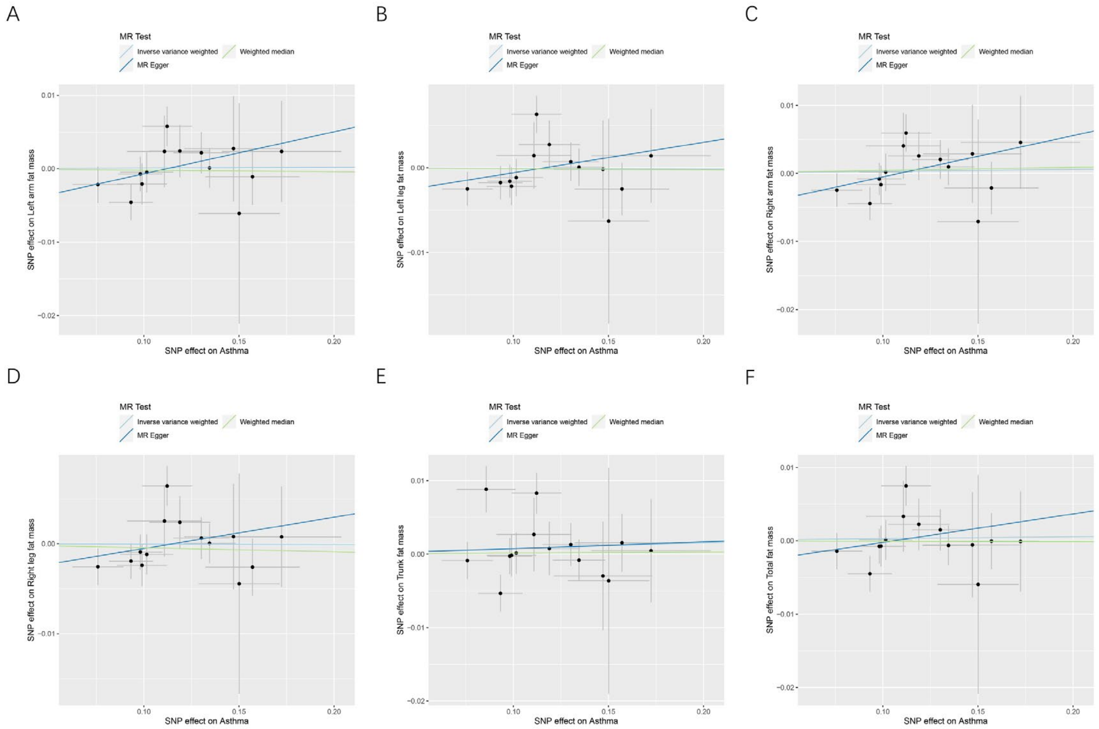

Supplementary Figure 22. Leave-one-out analyses for SNPs associated with asthma on Left arm fat mass. MR, mendelian randomization; SNPs, single nucleotide polymorphisms.

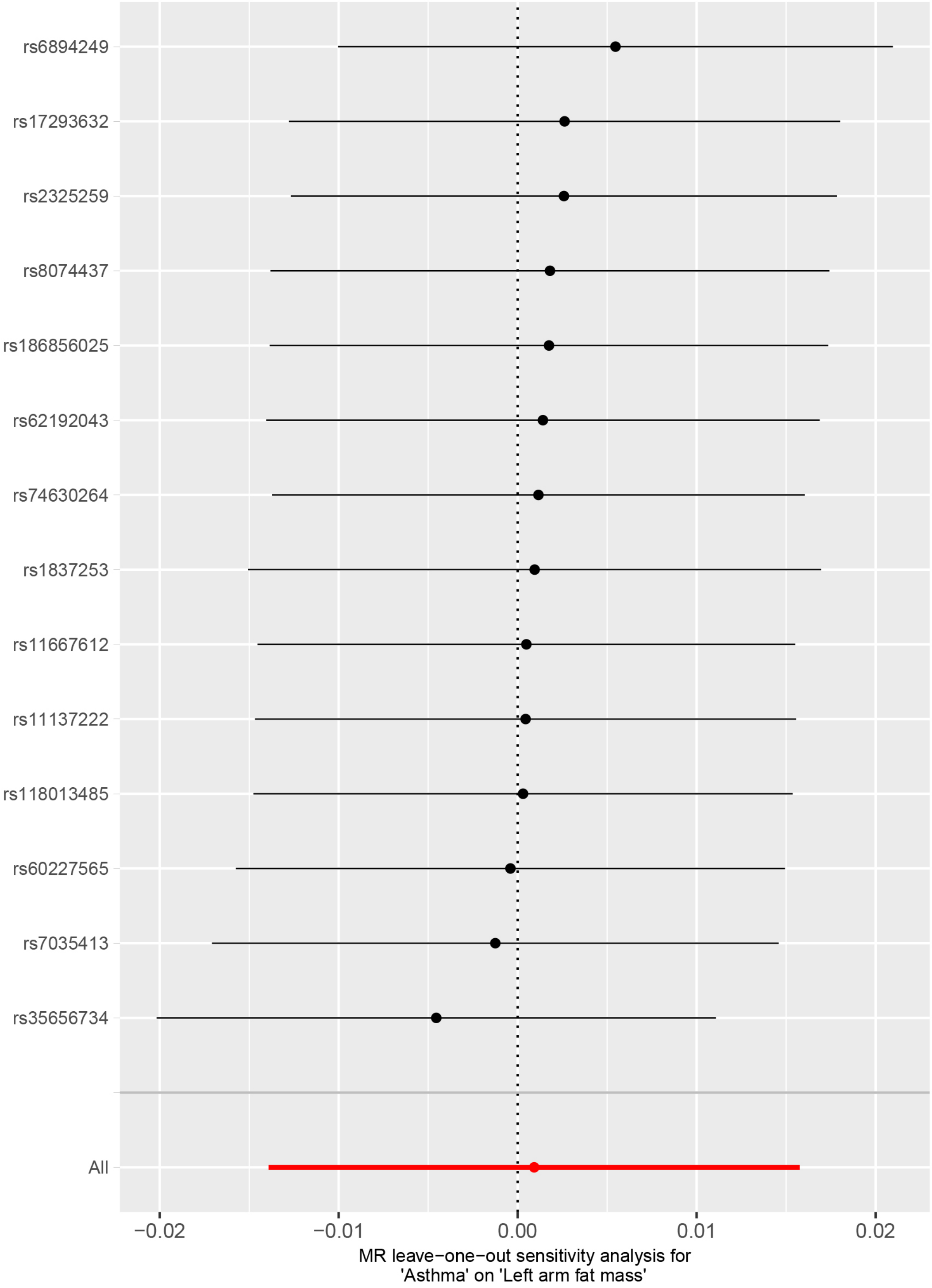

Supplementary Figure 23. Leave-one-out analyses for SNPs associated with asthma on Left leg fat mass. MR, mendelian randomization; SNPs, single nucleotide polymorphisms.

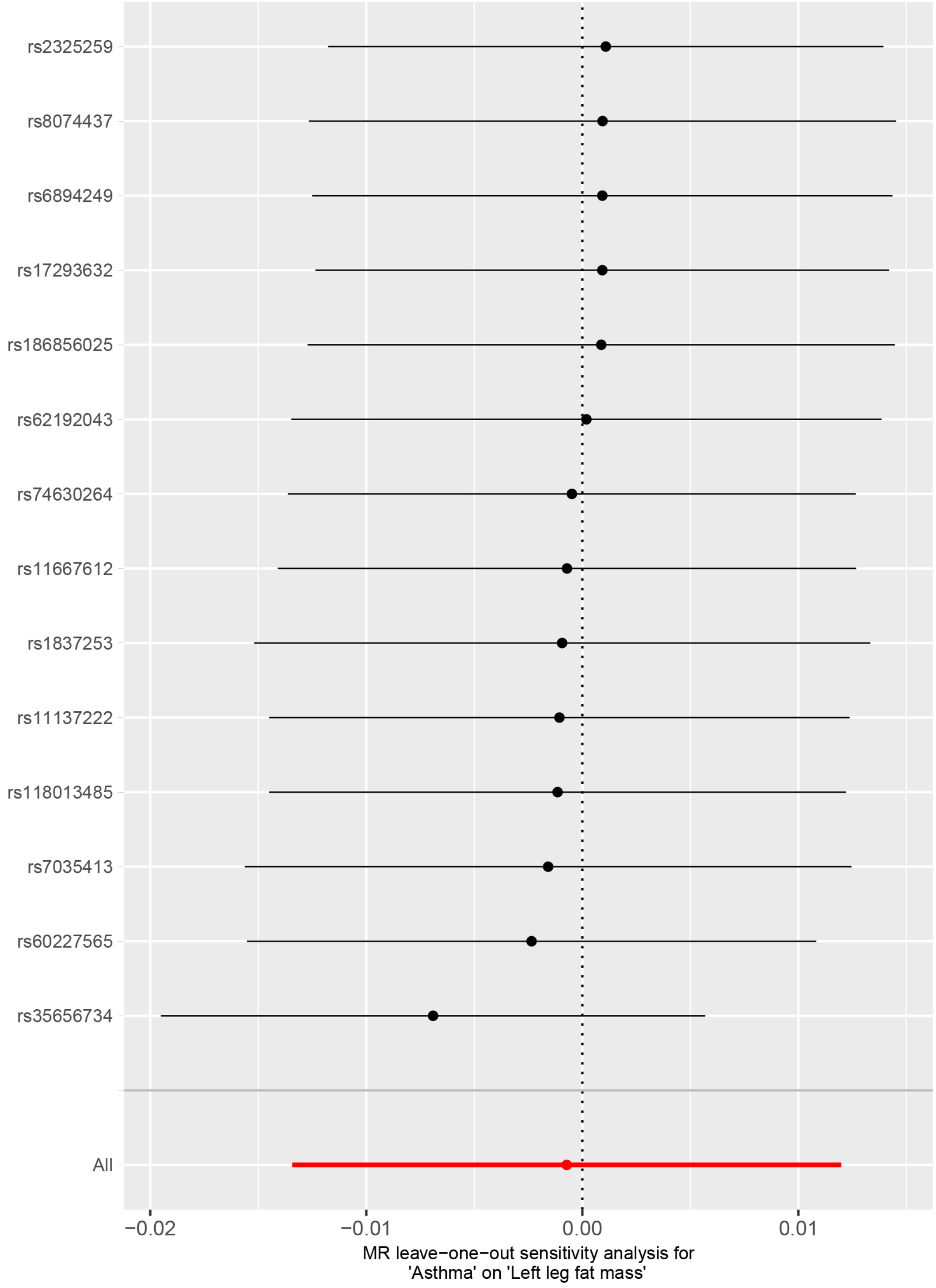

Supplementary Figure 24. Leave-one-out analyses for SNPs associated with asthma on Right arm fat mass. MR, mendelian randomization; SNPs, single nucleotide polymorphisms.

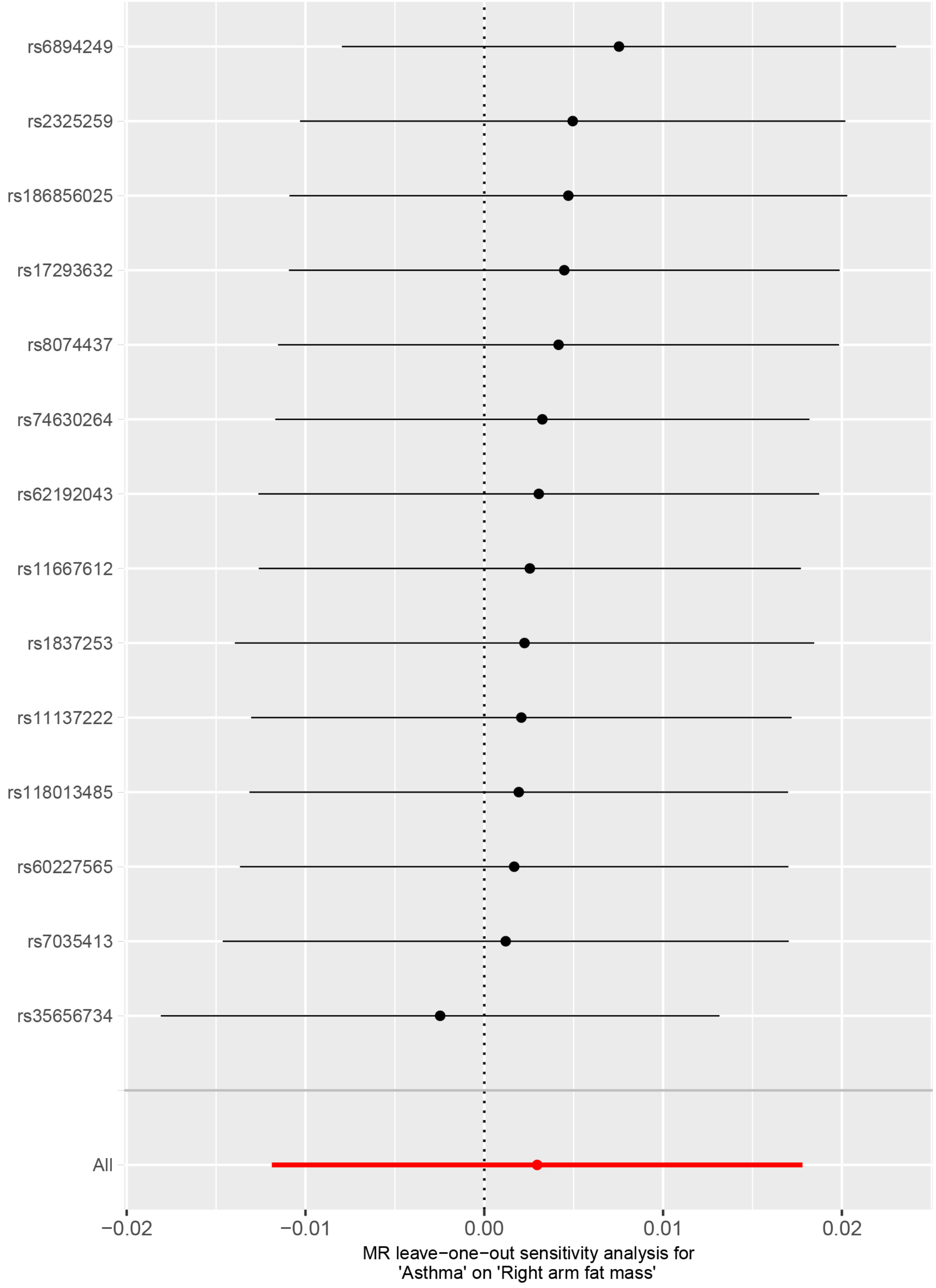

Supplementary Figure 25. Leave-one-out analyses for SNPs associated with asthma on Right leg fat mass. MR, mendelian randomization; SNPs, single nucleotide polymorphisms.

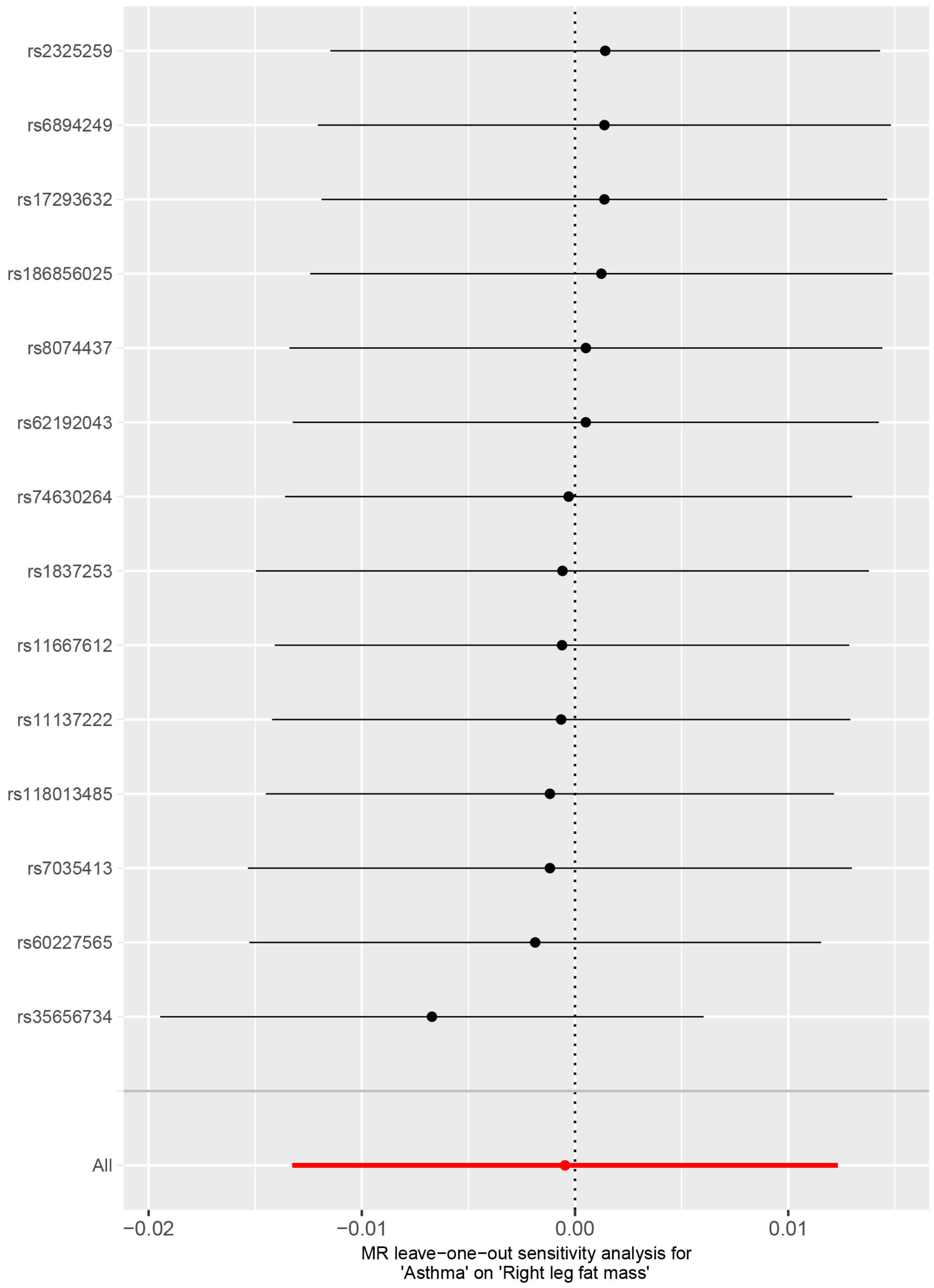

Supplementary Figure 26. Leave-one-out analyses for SNPs associated with asthma on Trunk fat mass. MR, mendelian randomization; SNPs, single nucleotide polymorphisms.

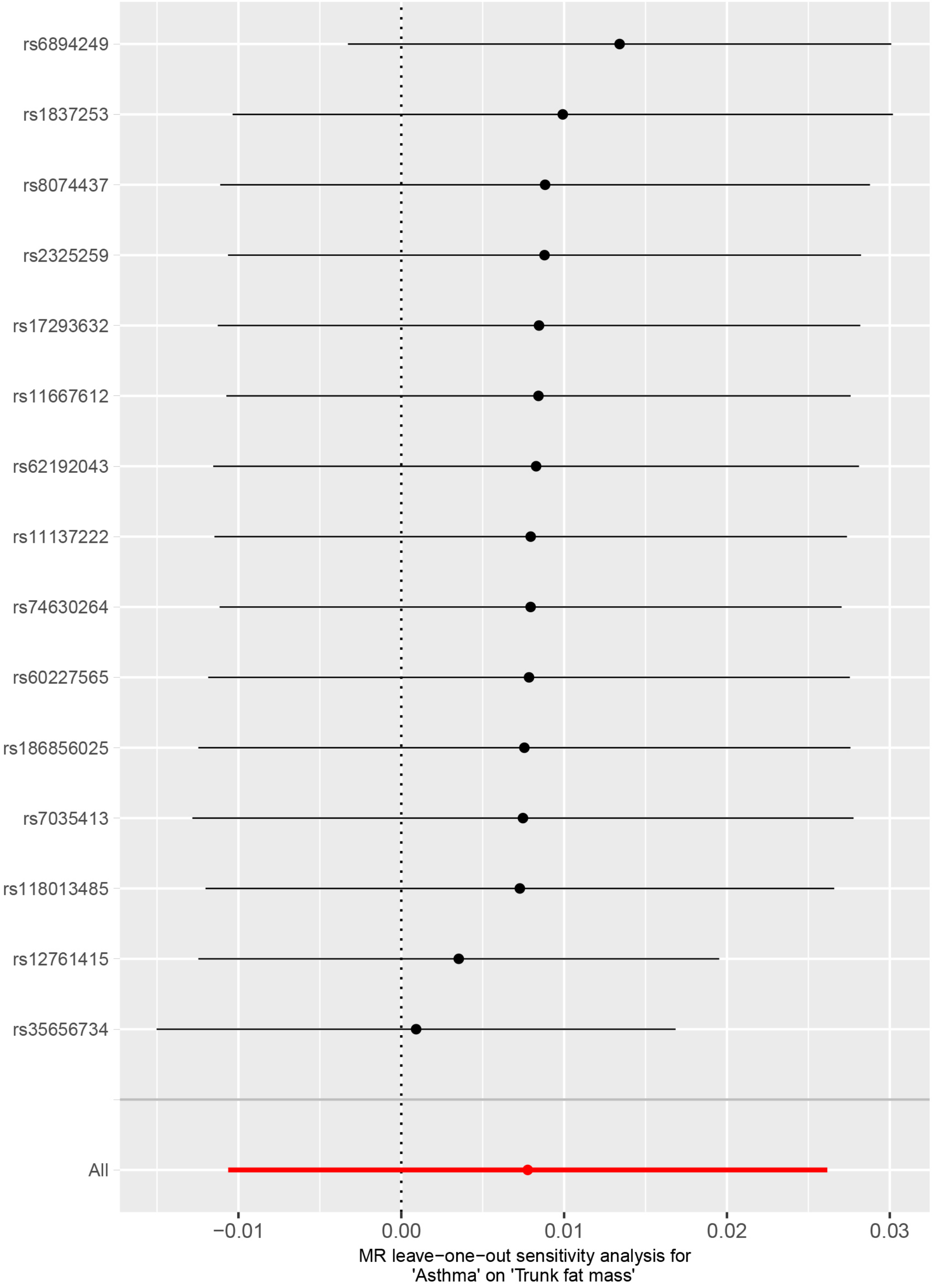

Supplementary Figure 27. Leave-one-out analyses for SNPs associated with asthma on Total fat mass. MR, mendelian randomization; SNPs, single nucleotide polymorphisms.

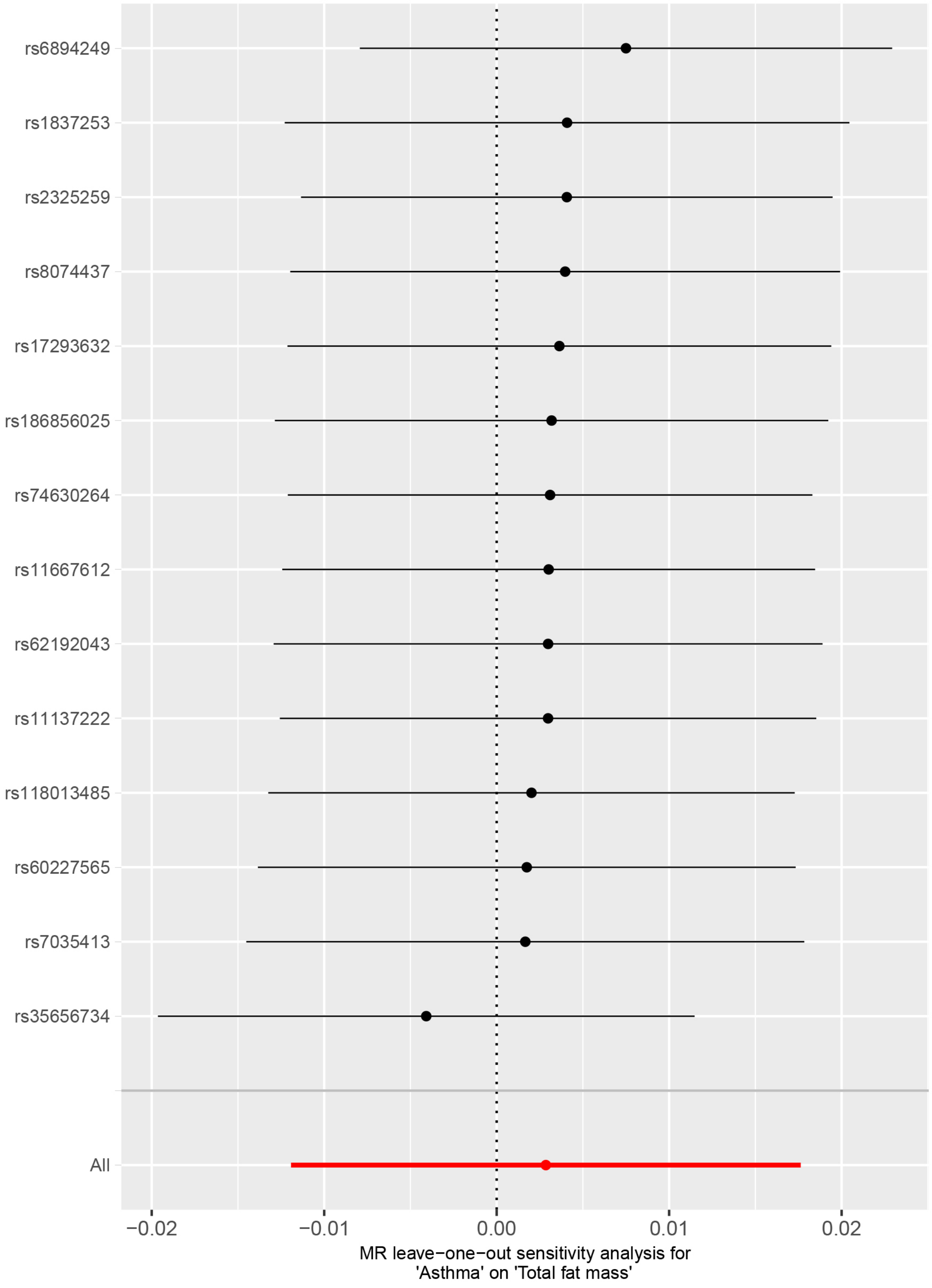

Supplementary Figure 28. Funnel plot of SNPs associated with asthma on body fat indexes. A, Left arm fat mass; B, Left leg fat mass; C, Right arm fat mass; D, Right leg fat mass; E, Trunk fat mass; F, Total fat mass. MR, mendelian randomization; SNPs, single nucleotide polymorphisms.

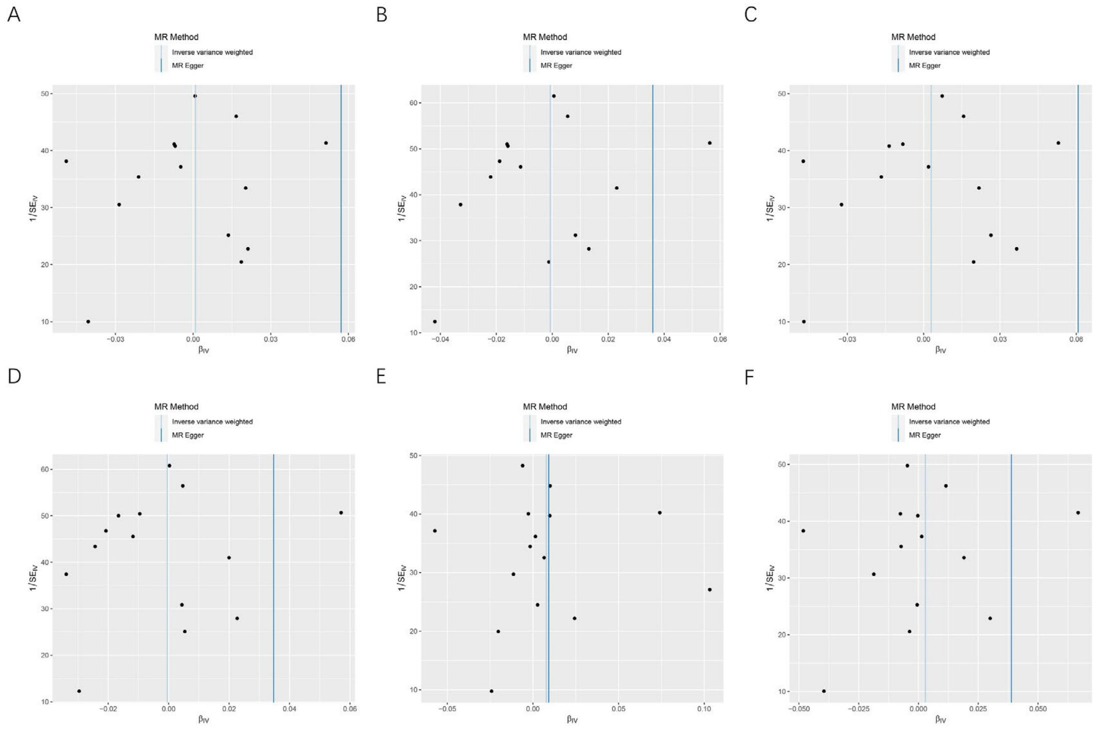

Supplement: Supplementary file 2 [file Image_1.pdf]
